# Artificial intelligence in neurodegenerative disease research: use of IBM Watson to identify additional RNA-binding proteins altered in amyotrophic lateral sclerosis

**DOI:** 10.1007/s00401-017-1785-8

**Published:** 2017-11-13

**Authors:** Nadine Bakkar, Tina Kovalik, Ileana Lorenzini, Scott Spangler, Alix Lacoste, Kyle Sponaugle, Philip Ferrante, Elenee Argentinis, Rita Sattler, Robert Bowser

**Affiliations:** 1 Department of Neurobiology, Barrow Neurological Institute, 350 W Thomas Road, Phoenix, AZ 85013 USA; 2 IBM Research-Almaden, San Jose, CA USA; 3 IBM Watson Health, New York, NY USA

**Keywords:** Amyotrophic lateral sclerosis, RNA-binding protein, Artificial intelligence, Protein aggregation, Motor neuron

## Abstract

Amyotrophic lateral sclerosis (ALS) is a devastating neurodegenerative disease with no effective treatments. Numerous RNA-binding proteins (RBPs) have been shown to be altered in ALS, with mutations in 11 RBPs causing familial forms of the disease, and 6 more RBPs showing abnormal expression/distribution in ALS albeit without any known mutations. RBP dysregulation is widely accepted as a contributing factor in ALS pathobiology. There are at least 1542 RBPs in the human genome; therefore, other unidentified RBPs may also be linked to the pathogenesis of ALS. We used IBM Watson^®^ to sieve through all RBPs in the genome and identify new RBPs linked to ALS (ALS-RBPs). IBM Watson extracted features from published literature to create semantic similarities and identify new connections between entities of interest. IBM Watson analyzed all published abstracts of previously known ALS-RBPs, and applied that text-based knowledge to all RBPs in the genome, ranking them by semantic similarity to the known set. We then validated the Watson top-ten-ranked RBPs at the protein and RNA levels in tissues from ALS and non-neurological disease controls, as well as in patient-derived induced pluripotent stem cells. 5 RBPs previously unlinked to ALS, hnRNPU, Syncrip, RBMS3, Caprin-1 and NUPL2, showed significant alterations in ALS compared to controls. Overall, we successfully used IBM Watson to help identify additional RBPs altered in ALS, highlighting the use of artificial intelligence tools to accelerate scientific discovery in ALS and possibly other complex neurological disorders.

**Electronic supplementary material:**

The online version of this article (10.1007/s00401-017-1785-8) contains supplementary material, which is available to authorized users.

## Introduction

Amyotrophic lateral sclerosis (ALS) is characterized by loss of motor neurons in the brain, brainstem and spinal cord, with concurrent muscle atrophy and is typically fatal within 2–5 years from diagnosis [1, 45]. The worldwide incidence of ALS is 1–3 cases per 100,000 individuals per year. However, considerable heterogeneity is associated with the disease at both the clinical and molecular levels, with variable sites of disease onset, variable rates of clinical disease progression, complex genetics, and a multitude of cell types involved in the disease process. Pathogenic cellular mechanisms are similarly multi-factorial, and include mitochondrial dysfunction, excitotoxicity, oxidative stress and presence of ubiquitinated neuronal and glial intracellular inclusions [52].

Approximately 10% of ALS is familial and genetic alterations in one of over 30 ALS genes have been linked to the disease [5, 7, 45, 52]. These familial ALS genes regulate a multitude of cellular processes, including cytoskeletal dynamics and membrane trafficking (*DCTN1*, *PFN1, VAP*), cellular proteostasis and autophagy (*SQSTM1*, *UBQLN2*, *OPTN*) and RNA metabolism (*TARDBP*, *FUS*, *MATR3*, *hnRNPA1*, *hnRNA2B1*, *TAF15*), while the most common genetic factor is the hexanucleotide repeat expansion in the *C9orf72* gene [20, 56].

Mutations or variants in the genes of 11 RNA-binding proteins (RBPs) or proteins that function in RNA processing are associated with ALS, including *TARDBP*, *FUS*, *hnRNPA1*, *hnRNPA2B1*, *MATR3*, *SETX*, *ELP3*, *ATXN2*, *ANG*, *SMN1*, and *SMN2* [7, 45, 52]. In addition, a number of other RBPs exhibit altered subcellular distribution in neurons and/or glia in ALS patients, but lack any known mutations that cause ALS [10, 37], suggesting that RBPs even without genetic alterations contribute to a disruption of RNA homeostasis in ALS. While mutations in the *TARDBP* gene are associated with only ~ 4% of familial ALS patients, TDP-43 protein mislocalization and inclusions are detected in 97% of all ALS patients [35]. This indicates that cytoplasmic and nuclear inclusions of RBPs are common in ALS, even without associated mutations. The number of RBPs currently associated with ALS represents a small fraction of the total RBPs, as a recent report identified 1542 putative RBPs in the human genome [21]. Given the large number of RBPs in the human genome and the number of RBPs that have already been linked to ALS, we hypothesized that additional RBPs contribute to and/or are mis-localized in ALS, and used IBM Watson to predict new potential candidates. IBM Watson was previously used to identify novel kinases that phosphorylate p53 and has contributed to the oncology field [48]. Since IBM Watson uses text-based information from abstract publications for its computational analysis [8], we were limited to RBPs that have been reported in the literature. 1478 RBPs were mentioned in at least one abstract published before the end of 2015, and these were included in our study.

To test the predictive modeling capability of IBM Watson, we first limited IBM Watson’s knowledge base to publications prior to 2013, and asked Watson to use this available information to predict other RBPs associated with ALS. Watson highly ranked the four RBPs with disease causing mutations identified between 2013 and 2017, demonstrating the validity of our approach. We then used IBM Watson to screen all known RBPs and predict RBPs likely to be associated with ALS based on their similarity to all known RBPs mutated in ALS. We validated Watson’s top-ten predictions by performing immunohistochemistry (IHC), protein and RNA expression analyses in brain and spinal cord tissues from ALS and non-neurologic disease controls, as well as RNA levels in motor neurons derived from induced pluripotent stem cells (iPSC-MNs) from ALS and controls. We also performed similar experiments for three RBPs near the bottom of the list that were predicted to not be altered in ALS as negative controls.

Eight of the top-ten RBPs predicted by Watson to be associated with ALS were altered in ALS by at least two validation methods listed above. During the course of this study, one of the RBPs predicted to be linked to ALS, Caprin-1, was shown to be altered in ALS patients [4]. As anticipated, RBPs ranked near the bottom of the list were not altered in ALS patients. Our results validate the IBM Watson predictions and identified novel RBPs altered in ALS. These findings further highlight the multitude of RBPs that contribute to the disruption of RNA homeostasis during ALS, and the strength of computer-based artificial intelligence approaches to accelerate wet lab scientific discoveries.

## Materials and methods

### Tissue samples

ALS and non-neurologic disease control post-mortem tissue samples were obtained from the University of Pittsburgh ALS Tissue Bank, the Barrow Neurological Institute ALS Tissue Bank, and the Target ALS Human Postmortem Tissue Core. All tissues samples were collected after informed consent from the subjects or by the subjects’ next of kin, complying with all relevant ethical regulations. The protocol and consent process were approved by the University of Pittsburgh Institutional Review Board (IRB) and the Dignity Health Institutional Review Board. Clinical diagnoses were made by board certified neuropathologists according to consensus criteria for ALS. Subject demographics are listed in Suppl. Table 5.

### Immunohistochemistry

Paraffin-embedded post-mortem tissue sections from spinal cords and cerebellum were used for this study. All sections were deparaffinized, rehydrated and antigen retrieval performed using Target Antigen Retrieval Solution, pH 9.0 (DAKO) or a citrate buffer (pH 6) for 20 min in a steamer. After cooling to room temperature, non-specific binding sites were blocked using Super Block (Scytek), supplemented with Avidin (Vector Labs). Primary antibodies used for immunohistochemistry were incubated overnight in Super Block with biotin (antibodies listed in Suppl. Table 3). Slides were then washed and incubated for 1 h in the appropriate biotinylated IgG secondary antibodies (1:200; Vector Labs) in Super Block. Slides were washed in PBS and immunostaining visualized using the Vectastain Elite ABC reagent (Vector Labs) and Vector NovaRED peroxidase substrate kit (Vector Labs). Slides were counterstained with hematoxylin (Sigma Aldrich). Sections were visualized using a Leica AperioScope microscope, and analyzed using the Aperio eSlide manager image analysis.

For color intensity analysis, regions of interest (ROI) were delineated by a blinded user (motor neuron nuclei or Purkinje nuclei), slides were deconvolved for RGB of hematoxylin (blue channel) and antibody staining color (red channel) using the Leica Aperio ImageScope color deconvolution algorithm and the intensity value measured for each pixel within the ROI. These values were used to set intensity scales for each color from 0 to 255 (0 = black and 255 = white) prior to the analysis, and the same intensity thresholds were used across each antibody analysis. For hnRNPU, the negative threshold was set to be for intensities ranging from 210 to 255; weak positive staining intensity ranges were from 145 to 210; medium positive staining from 90 to 145 and strong staining was set to ranges from 0 to 90. All neurons were selected for each spinal cord section (numbers of neurons per section ranged from 20 to 50), and ROIs were defined. For Syncrip, the negative threshold was set to be for intensities ranging from 180 to 255; weak positive staining intensity ranges were from 155 to 180; medium positive staining from 95 to 155 and strong staining was set to ranges from 0 to 95. We selected 50 Purkinje cells from different areas of each section.

### Laser-capture microscopy, RNA extraction and real-time PCR analysis

Lumbar spinal cord and cerebellum total RNA were prepared from frozen tissue from control and ALS cases. Samples were homogenized in Trizol (Invitrogen), and RNA was extracted using the Ambion PureLink™ RNA Mini Kit. RNA quality was determined by RIN (RNA integrity number) using a Tapestation and all samples showed RIN values of > 5. cDNA was synthesized using Superscript VILO (Invitrogen) and real-time RT-PCR was performed using the FastStart Universal SyberGreen master mix (Roche). Primer sequences used are listed in Suppl. Table 7.

For laser-capture microscopy, fresh-frozen cerebellum were sectioned at 20 μm, slides were fixed for 2 min in 70% ethanol (in nuclease-free water), washed and stained for 6 min with the RNA/DNA stain Methyl Green Pyronin (Abcam, ab150676) supplemented with SUPERase In RNAse inhibitor (AM2694, ThermoFisher). Slides were consecutively dipped in nuclease-free water, 100% ethanol, and air-dried for 2 min before capture. We used the Zeiss Axiovert Zoom, fitted with a PALM system to capture at least 120 Purkinje cells per slide. Capture time was limited to 1 h to minimize RNA degradation. Two slides from each sample were used for a total of 250 neurons, and the cells were combined for subsequent processing. RNA was extracted using the RNAqueous micro total RNA isolation kit from Ambion (AM1931), cDNA was synthesized using Superscript VILO and real-time RT-PCR was performed.

### Statistical analysis

Statistical analysis was performed using Student’s *t* test, or one-way ANOVA with Bonferroni’s multiple comparisons testing for comparing multiple groups in GraphPad Prism 5. Fisher exact test and Wilcoxon rank sum test were used for cross-validation studies.

### Data and code availability

All data generated or analyzed during this study are included in the published article and its supplementary information files (Suppl. Tables 2, 3 and 4). The pseudo-code used to generate our analysis by IBM Watson is included in the supplementary information files.

## Results

### IBM Watson analytics and model generation

A detailed description of the analytical methods used by Watson to predict RBPs associated with ALS is provided in the Supplemental Materials and Methods section. Briefly, IBM Watson extracts domain-specific text features from published literature to identify new connections between entities of interest, such as genes, proteins, drugs, and diseases. From these annotated documents, Watson creates a semantic model of the known set of RBPs previously linked to ALS, and then applies that model to a candidate set of all other RBPs, in order to rank all the candidates by similarity to the known set using a graph diffusion algorithm [58]. To test the model generation by Watson, we first performed a leave-one-out (LOO) cross-validation to demonstrate the predictive power of Watson using the 11 known ALS-linked RBPs. To do so, the graph diffusion algorithm was applied 11 times based on the same distance matrix, but each time a different known RBP was taken out of the positive set and placed into the candidate set. If the overall model is accurate, then the positive RBPs placed into the candidate set should rank high based on the model built from the other ten known positive RBPs. Indeed, in our experiment, the LOO cross-validation results were strong, with 5 of the positive RBPs ranking in the top-15 out of 1478 RBPs, and 8 in the top 4.1% of all RBPs (*p* = 3.17 × 10^−7^, one-sided Wilcoxon rank sum test to assess whether the scores of the knowns are greater than those of the candidates, Table 1).

**Table 1:** Leave-one-out (LOO) cross-validation test

Protein	Rank
TARDBP	1
FUS	5
SETX	11
MATR3	12
TAF15	13
ATXN2	21
HNRNPA2B1	60
ARHGEF28	61
HNRNPA1	106
GLE1	107
ANG	713

Fisher exact test *p* value (1469,48,11,7) = 7.62E−09. The rank column corresponds to the rank of the RBP when removed from the known set and placed into the candidate set. The *p* value corresponds to the median *p* value of individual Fisher’s exact tests done at each known RBP rank threshold

To measure accuracy of the model based on the LOO cross-validation, we used the receiver operating characteristic (ROC) curve. The area under the ROC curve (AUC, computed following the trapezoid rule) is a measure of model accuracy, where a value of 0.5 corresponds to a random model and a value of 1 corresponds to a perfectly predictive model. The AUC for our model was 0.935.

An added value of the LOO cross-validation is that it provides a point of reference for where to expect true ALS-related RBPs from the candidate set to be listed within the overall ranking. In the cross-validation performed by Watson, 10 of the 11 positive RBPs (90%) ranked within the top 8% of all RBPs. ANG was ranked number 713 by this analysis, suggesting that this gene is dissimilar to known ALS-associated RBPs. Extrapolating these results to all known and yet-to-be discovered RBPs, we can expect that approximately 90% of all true positive ALS-linked RBPs should fall within the top 8% of the ranked list.

### Retrospective analysis validates Watson’s RBP prediction model

We next used a retrospective study to test IBM Watson’s analytics for predicting RBPs in ALS. We restricted the corpus of data analyzed by Watson to literature published prior to 2013, and used as a positive known set the eight RBPs with ALS disease causing mutations that were published through 2012 (Table 2). We used all 1542 RBPs in the genome listed in Gertsberger et al. [21], as candidate RBPs. 1439 of these RBPs had at least one mention in Medline^®^ abstracts up to the end of 2012 and these were thus chosen to be our candidate set. IBM Watson built a distance matrix relating each RBP with all others and used a graph diffusion algorithm to rank all RBPs based on the known set of eight RBPs [58]. The RBPs found to be mutated in familial ALS and published between 2013 and 2017 were ARHGEF28, Matrin 3, GLE1, and TIA1 [16, 27, 28, 36]. We asked if Watson could predict these RBPs as high-ranking candidates. The results from this retrospective analysis identified Matrin 3 as the top candidate, ARHGEF28 and TIA1 ranked within the top 5%, and GLE1 ranked within the top 11% of all known RBPs (Table 2), thus demonstrating the performance capabilities of the model. Another RBP linked to ALS in 2014 with no known mutations, hnRNPA3, was also ranked within the top ten in this retrospective study.

**Table 2:** IBM Watson retrospective analysis

Known Gene set
TARDBP
FUS
ATXN2
ANG
SETX
hnRNPA2B1
hnRNPA1
TAF15

Candidate Gene set	Score (GD)	Rank
** MATR3**	0.00204078	1
NUPL2	0.00181635	2
SRSF2	0.0017781	3
SYNCRIP	0.00175763	4
hnRNPU	0.00174455	5
RBM6	0.00161879	6
IGHMBP2	0.00154716	7
** hnRNPA3**	0.00154361	8
hnRNPC	0.00153549	9
hnRNPM	0.00151568	10
–		
** RBM45**	7.79E−04	43
** TIA1**	7.76E−04	50
** ARHGEF28**	3.95E−04	89
** GLE1**	3.85E−04	165

All genes are listed using the HGNC database of human gene names and protein coding genes. The known set refers to RBPs shown to be mutated in ALS prior to 2013. The candidate set refers to all RBPs with at least one published abstract, analyzed and ranked by Watson. GD = graph diffusion score assigned by Watson to each gene/protein based on semantic similarity of the candidate to the positive known set. Genes/proteins in bold have been linked to ALS: MATR3, ARHGEF28, TIA1 and GLE1 were shown to be mutated in the past 4 years; RBM45 and hnRNPA3 exhibit alterations in ALS tissues with no described mutations

### IBM Watson ranks RBPs by semantic similarity to RBPs mutated in ALS

After having established that IBM Watson methodology is valid and capable of identifying RBPs involved in ALS, we next analyzed all 1478 RBPs from the Gertsberger et al. [21] list that had at least one mention in Medline^®^ abstracts up to the end of 2015 as our candidate set. The known set included all 11 known RBPs mutated in ALS identified prior to 2016 (TDP-43, FUS, Ataxin-2, hnRNPA1, hnRNPA2B1, Senataxin, Angiogenin, TAF15, GLE1, Matrin-3 and ARHGEF28). We excluded from the known set RBPs such as RBM45, hnRNPA3, or MTHFSD that have been shown to be altered in ALS tissue samples, but without any mutations described to date [6, 10, 37, 40]. IBM Watson rank ordered RBPs by similarity to the known set, and assigned a score from 0 to 1, corresponding to how closely related each RBP is to the overall set of 11 positive RBPs (see “Materials and methods” for more details). Results of the ranked proteins along with their graph diffusion (GD) scores are shown in Table 3 and Supplemental Table 1. Among the top-ten-ranked RBPs was RBM45, which our group previously reported to localize to cytoplasmic inclusions in ALS cases [10]. In addition, MTHFSD, an RBP reported by the Robertson group in 2016 to be a novel component of stress granules and altered in ALS was ranked at number 10 by Watson [37]. Other RBPs previously linked to ALS and ranked in the top 5% included hnRNPA3, SMN2 and EWSR1 [13, 14, 40] (Table 3).

**Table 3:** IBM Watson prospective analysis

Known Gene set
ANG
FUS
TARDBP
ATXN2
HNRNPA1
HNRNPA2B1
SETX
TAF15
GLE1
MATR3
ARHGEF28

Candidate Gene set	Score (GD)	Rank
hnRNPU	0.002914	1
SYNCRIP	0.002747	2
** RBM45**	**0.00268**	**3**
RBMS3	0.002494	4
SRSF2	0.002459	5
hnRNPH2	0.002255	6
NUPL2	0.002152	7
CAPRIN1	0.002109	8
RBM6	0.001915	9
** MTHFSD**	**0.00191**	**10**
–		
** hnRNPA3**	**0.001534**	**18**
–		
** SMN2**	**7.72E−04**	**63**
** EWSR1**	**7.71E−04**	**66**

All genes are listed using the HGNC database of human gene names and protein coding genes. The known set refers to RBPs shown to be mutated in ALS prior to 2016. The candidate set refers to all RBPs with at least one published abstract, analyzed and ranked by Watson. GD = graph diffusion score. Genes/proteins in bold exhibited prior links to ALS, albeit without any mutations described thus far

We focused validation studies on the top-ten-ranked RBPs and asked whether they were altered in ALS. Validation methods included protein subcellular distribution using IHC, measures of protein levels by immunoblot, RNA levels by total tissue extracts and laser-captured microdissection, and RNA analysis of motor neurons generated from patient-derived iPS cells. RBPs had to exhibit statistically significant differences between ALS and controls by at least two methods to be termed validated in our study. Our top-ten proteins included two previously shown to be altered in ALS (RBM45, and MTHFSD), so we did not pursue these further. Unsurprisingly, many of the IBM Watson top-ten-ranked RBPs are involved in RNA processing and export, and four out of the eight proteins we validated were contained within supplemental tables of proteins that potentially interact with TDP-43 and/or FUS in recent proteomic studies (Table 4). It is noteworthy that these interactions were listed in supplemental materials within these publications, not within the published abstracts, and as such, the putative protein interactions were not made available to IBM Watson for its analysis. The Watson top-ranked RBP, hnRNPU, possesses both RNA and DNA binding domains and potentially interacts with TDP-43, FUS, ubiquilin2 and the G4C2 repeat of C9orf72 [4, 19, 23, 24, 34, 49]. In addition, hnRNPU was recently shown to modulate nuclear TDP-43 toxicity in cultured cells [49], but was not directly linked to ALS. The second-ranked protein, Syncrip, is an RBP that resides in the cytoplasm and has been identified as an SMN-interacting protein found in RNA granules [34, 46, 53]. It was identified by mass spectrometry-based proteomics as a potential interacting protein with TDP-43, Ataxin-2, FUS, optineurin and ubiquilin, but again was never studied in ALS [4]. RBMS3 (ranked 4), hnRNPH2 (ranked 6) and RBM6 (ranked 9) all function in RNA metabolism or processing and have no prior links to ALS. NUPL2 (ranked 7), a nucleoporin-like protein interacts with Gle1, functions in CRM1-mediated RNA export and is a risk locus for Parkinson’s disease [15, 30, 41]. SRSF2 (also known as SC-35, ranked 5) is a nuclear speckle component that potentially interacts with TDP-43, FUS and the G4C2 repeat expansion and has been shown to co-localize with 34% of C9 antisense RNA foci in cerebellar Purkinje cells of C9-ALS patients [12]. Caprin-1 (ranked 8) is an RBP involved in neuronal RNA transport that interacts with FMRP and G3BP [17, 47]. Recently, a comprehensive proteomic study investigating common interactors for TDP-43, FUS and Ataxin-2 identified Caprin-1 as interacting with all 3 proteins [4]. In addition, they demonstrated that Caprin-1 co-localized with TDP43 and FUS inclusions in spinal cord motor neurons from 3 patients with TDP-43 inclusions and 2 patients with FUS-R521C mutations, respectively [4]. These results were published after our Watson analysis of the literature (Caprin-1 also does not appear in the abstract) and confirms our IBM Watson prediction that Caprin-1 is altered in ALS.

**Table 4:** Literature summary of top-ten-ranked RBPs

Protein	Full name	Alternative names	Rank	Direct link to ALS prior to 2016	Potential links to ALS	Function and features	References
hnRNPU	Heterogeneous nuclear ribonucleoprotein U	Scaffold attachment factor A (SAF-A)	1	None	Potential binding to TDP-43, FUS, Ataxin, OPTN, Ubqnl2 and G4C2 repeat; modulates TDP-43 nuclear toxicity	mRNA splicing/DNA–RNA scaffold	[4, 19, 23, 24, 34, 49]
Syncrip	Synaptotagmin-binding cytoplasmic RNA interacting protein	hnRNPQ; GRY-RBP	2	None	Potential binding to TDP-43, FUS, Ataxin, OPTN, Ubqnl2—binds SMN	mRNA processing and cytoplasmic transport	[4, 34, 46, 53]
RBM45	RNA-binding motif 45 protein		3	Yes			[10]
RBMS3	RNA-binding motif single-stranded interacting protein 3	–	4	None	None	RNA metabolism in the cytoplasm-tumor suppressor gene for lung squamous cell carcinoma	[33]
SRSF2	Serine- and arginine-rich splicing factor 2	Splicing component, 35 kDa (SC-35); SFRS2	5	Yes: co-localizes with 34% of C9 antisense RNA foci in Purkinje cells from C9-ALS patients	Potential binding to TDP43, Fus, and G4C2 repeat	mRNA splicing and export from the nucleus; found in nuclear speckles	[12, 32, 39, 54]
hnRNPH2	Heterogeneous nuclear ribonucleoprotein H2		6	None	None	RNA processing-mutations associated with neurodevelopmental diseases in females	[3]
NUPL2	Nucleoporin like 2	NLP-1; nucleoporin-like protein 1; hCG1	7	None	Interacts with Gle1	mRNA export from the nucleus (CRM1-mediated)—risk loci for Parkinson’s disease	[15, 30, 41]
CAPRIN1	Cell cycle-associated protein 1	GPI-anchored membrane protein 1; GPI-anchored protein P137; RNA granule protein 105; cytoplasmic activation/proliferation-associated protein-1	8	None	Interacts with TDP-43, Ataxin2, FUS, FMRP, and G3BP	RNA transport in neurons/stress granule	[4, 17, 47]
RBM6	RNA-binding motif protein 6	Lung cancer protooncogene 11; RNA-binding protein DEF-3	9	None	None	RNA processing in splicing speckles	[25]
MTHFSD	Methenyltetrahydrofolate synthetase domain containing		10	Yes—published in 2016^a^			[37]

^a^MTHFSD was published to be altered in ALS tissues in print version January 2016, but was available as a pre-print online in December 2015, and as such was available for our Watson analysis

### Localization of top-ranked IBM Watson RBPs in spinal cord and cerebellum confirms their alterations in ALS

We validated the top-ten-ranked RBPs using immunohistochemistry (IHC) of lumbar spinal cord and cerebellum from SALS, C9-ALS and non-neurologic disease controls. RBM45 and MTHFSD were previously shown to be altered in ALS spinal cord using a similar IHC approach [10, 37]. The recent discovery of G4C2 repeat foci in the cerebellum [2], along with global splicing changes in both SALS and C9-ALS cerebellum [44] prompted us to examine potential RBP changes in the cerebellum. To test the specificity of IBM Watson results, we also performed immunohistochemistry for three RBPs from the bottom of the list (QTRT1, NARS and WARS). Our IHC results are summarized in Table 5.

**Table 5:** Validation studies summary for top-ten IBM Watson protein

Protein	Rank	Direct link to ALS prior to 2016	Immunohistochemistry		Changes at the RNA level in tissues		Changes at the RNA level in neurons		Changes at the protein level in tissues	
			Altered localization—spinal cord	Altered localization—cerebellum	RNA alterations—spinal cord	RNA alterations—cerebellum	RNA alterations—hiPSC-MN	RNA alterations—LCM Purkinje	Protein alterations—spinal cord	Protein alterations—cerebellum
hnRNPU	1	None	Yes	None	None	Yes—decreased	None	Yes—trend^b^	Yes—trend^b^	Yes—trend^b^
SYNCRIP	2	None	Inconclusive ^a^	Yes	None	Yes—decreased	Yes—increased in C9	Yes—trend^b^	None	Yes—increased
RBM45	3	Yes	–	–	–	–	–	–	–	–
RBMS3	4	None	Inconclusive ^a^	Yes	Yes—decreased	None	None in C9—yes in SALS (decreased)	None	Yes—increased	Yes—increased
SRSF2	5	Yes	Yes	Inconclusive	None	None	None	None	None	None
hnRNPH2	6	None	None	None	None	Yes—decreased	None	None	None	None
NUPL2	7	None	Inconclusive ^a^	Yes	None	Yes—decreased	None	None	Yes—decreased	None
CAPRIN1	8	None	Yes	Yes	None	None	Yes—increased in C9	None	Yes—decreased	None
RBM6	9	None	Yes	None	None	None	None	None	None	None
MTHFSD	10	Yes	–	–	–	–	–	–	–	–

^a^Variability in staining pattern and intensity in control motor neurons even within the same case

^b^Trend towards an increase in ALS; not statistically significant

**Fig. 1:**
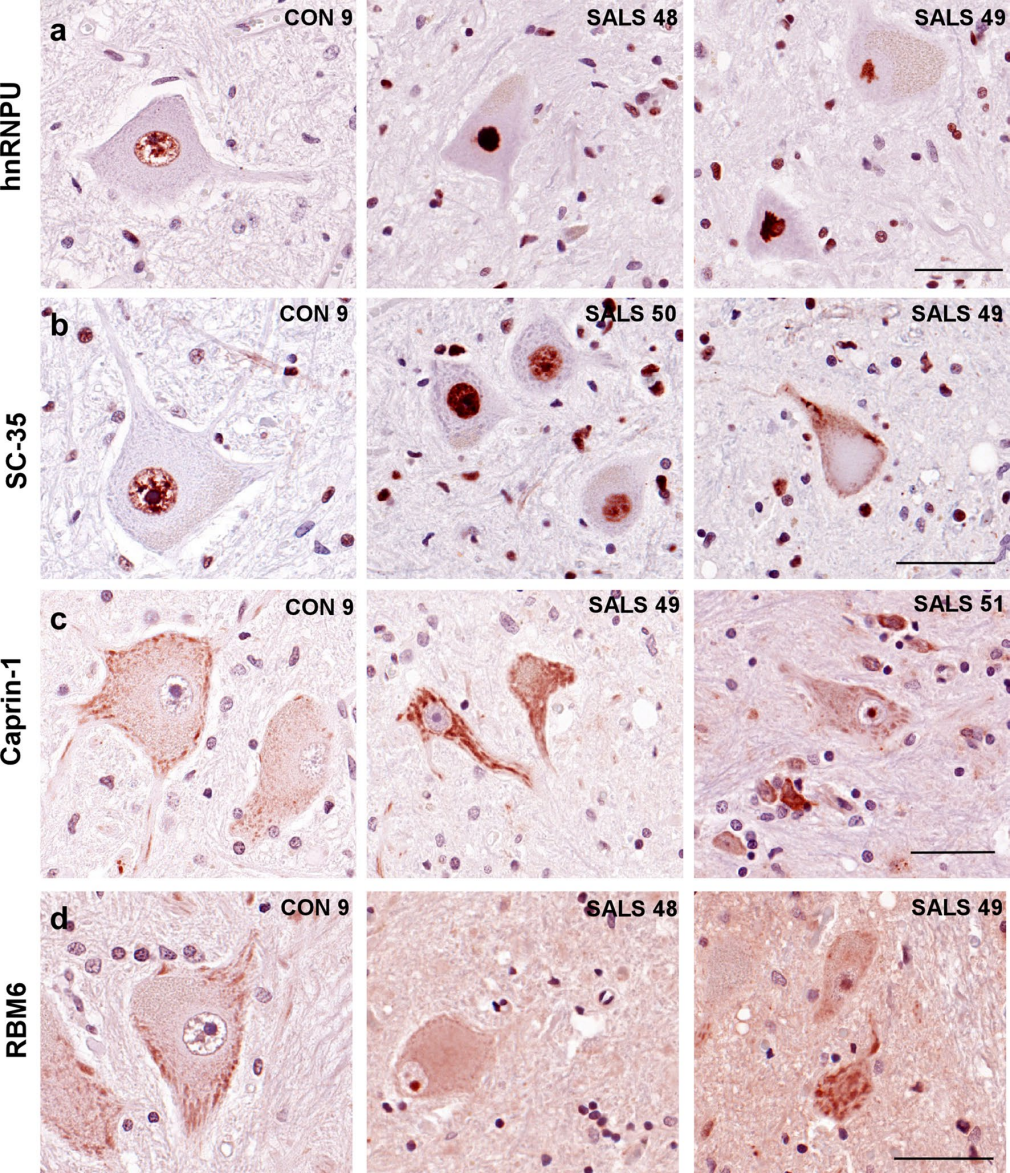
Immunolocalization of IBM Watson top-ranked RBPs in lumbar spinal cord. IHC for hnRNPU, SC-35, Caprin-1 and RBM6 in the lumbar spinal cord of 4 C9-ALS, 4 non-neurological disease controls and 6–14 SALS cases. Representative images of motor neurons are shown, counterstained with hematoxylin. **a** Control motor neurons stained with hnRNPU show weak nuclear staining, while ALS motor neurons exhibit either strong nuclear staining, or cytoplasmic thread-like inclusions. **b** SRSF2/SC-35 labeled nuclear speckles in control motor neurons. ALS neurons exhibit a variety of phenotypes ranging from large dark speckles, to strong nuclear staining, and rare cytoplasmic inclusions and neuropil staining in one ALS case (SALS 49). **c** Caprin-1 labels cytoplasmic granules in control motor neurons, with larger granules and strong immunostaining in ALS neurons. In addition, most SALS cases but no C9-ALS cases exhibit Caprin-1 staining in the nucleolus. **d** RBM6 is negative or weak in control motor neurons, while ALS cases exhibit nucleolar staining. All images were taken at ×40 magnification. Scale bar: 50 μm

IHC for hnRNPU was performed and nuclear staining pixel intensity was quantified as described in the “Materials and methods”, and reported as negative, weak, medium or strong immunoreactivity. hnRNPU exhibited negative or weak immunoreactivity in 70–80% of spinal cord motor neuron nuclei of control cases (Figs. 1a, 3a). Conversely, spinal motor neurons in SALS cases displayed strong hnRNPU nuclear staining in a majority (50–85%) of neurons per case. C9-ALS cases exhibited medium hnRNPU signal intensity that was significantly different from SALS neurons but failed to show significance when compared to controls, likely due to one of the of four C9-ALS cases being negative for hnRNPU. In addition, we observed increased glial staining in ALS compared to controls (Fig. 1a), and multiple cytoplasmic as well as nuclear hnRNPU inclusions in both SALS and C9-ALS motor neurons that co-localized with TDP-43 (Fig. 4a). In the cerebellum, hnRNPU showed weak immunostaining in nuclei of Purkinje and granule cells of control subjects, while three out of five C9-ALS cases displayed medium-to-strong IHC patterns (Suppl. Figure 2c). Purkinje cells in SALS had variable staining intensities, ranging from negative to strong.

**Fig. 2:**
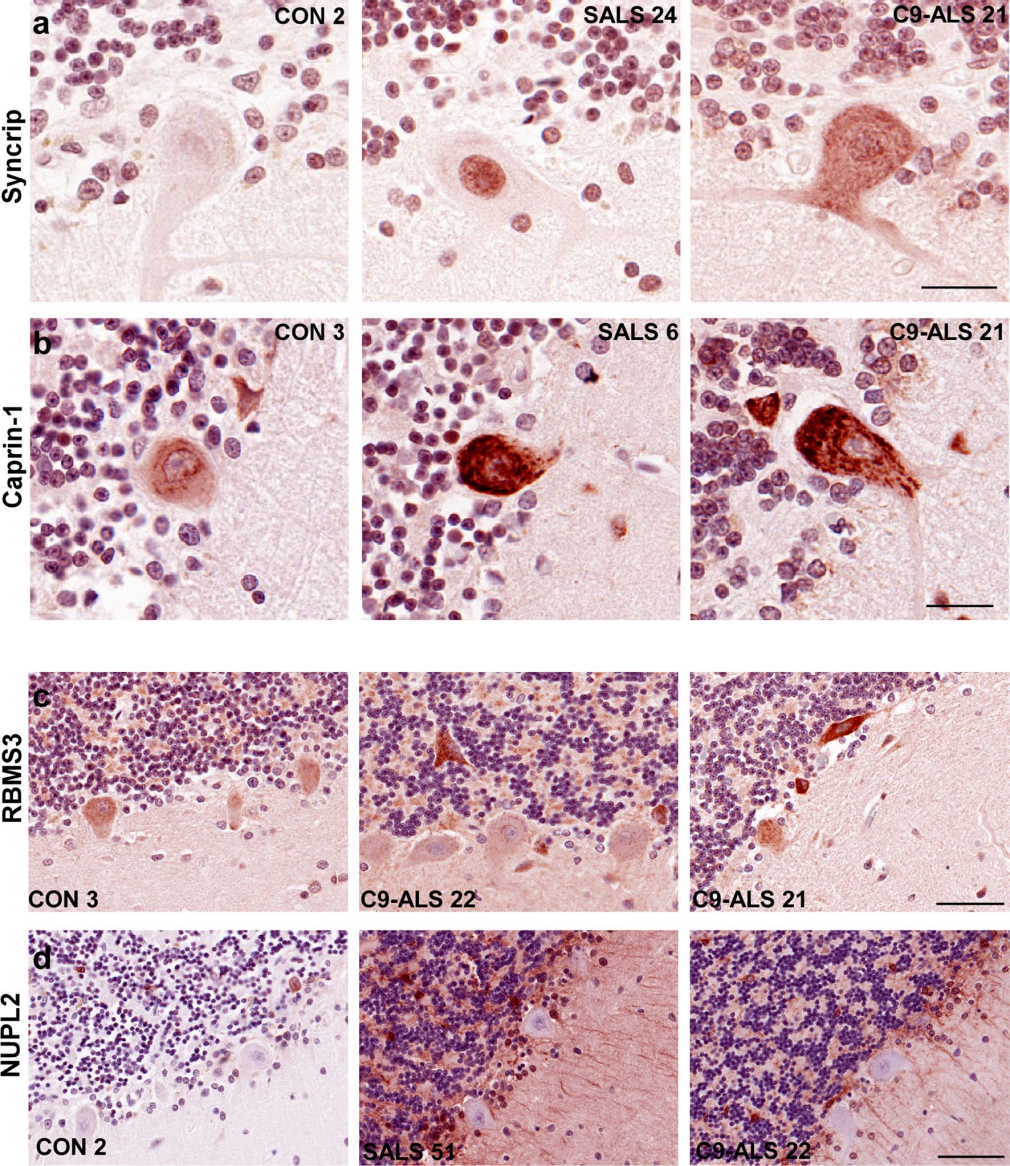
Immunolocalization of IBM Watson top-ranked RBPs in the cerebellum. IHC for Syncrip, Caprin-1, RBMS3 and NUPL2 in 4 C9-ALS, 3–5 non-neurological disease controls and 8 SALS cases. Representative images are shown, counterstained with hematoxylin. **a** Weak Syncrip-labeled nuclei in control Purkinje cells, as well as granule cell nuclei in one out of five control cases. C9-ALS Purkinje cells exhibited medium-to-strong diffuse cytoplasmic Syncrip staining (four out of four), while SALS displayed weak granule cell and strong Purkinje cell nuclear staining. **b** Weak Caprin-1 IHC in four out of five control cerebellum; while Purkinje cells in C9-ALS (four out of four cases) and SALS (four out of seven) exhibited medium-to-strong cytoplasmic staining. **c** Negative-to-weak RBMS3 IHC in control cerebellum with three out of five cases showing no interneuron staining, while three out of five had some granular layer interstitial immunostaining. Interneurons in the Purkinje, molecular and granule cell layers displayed strong RBMS3 IHC in all C9-ALS and SALS cases. In addition, six ALS case showed some strong inter-granule cell staining, while three cases had RBMS3 staining in Purkinje cells. **d** NUPL2 was negative to weak in all controls, but labeled astrocytes in all SALS and one out of four C9-ALS cases examined. Purkinje cells were negative for NUPL2 in all subject groups. All images were taken at ×40 magnification. Scale bar: 25 μm for **a**, **b,** 50 μm for **c** and 70 μm for **d**

**Fig. 3:**
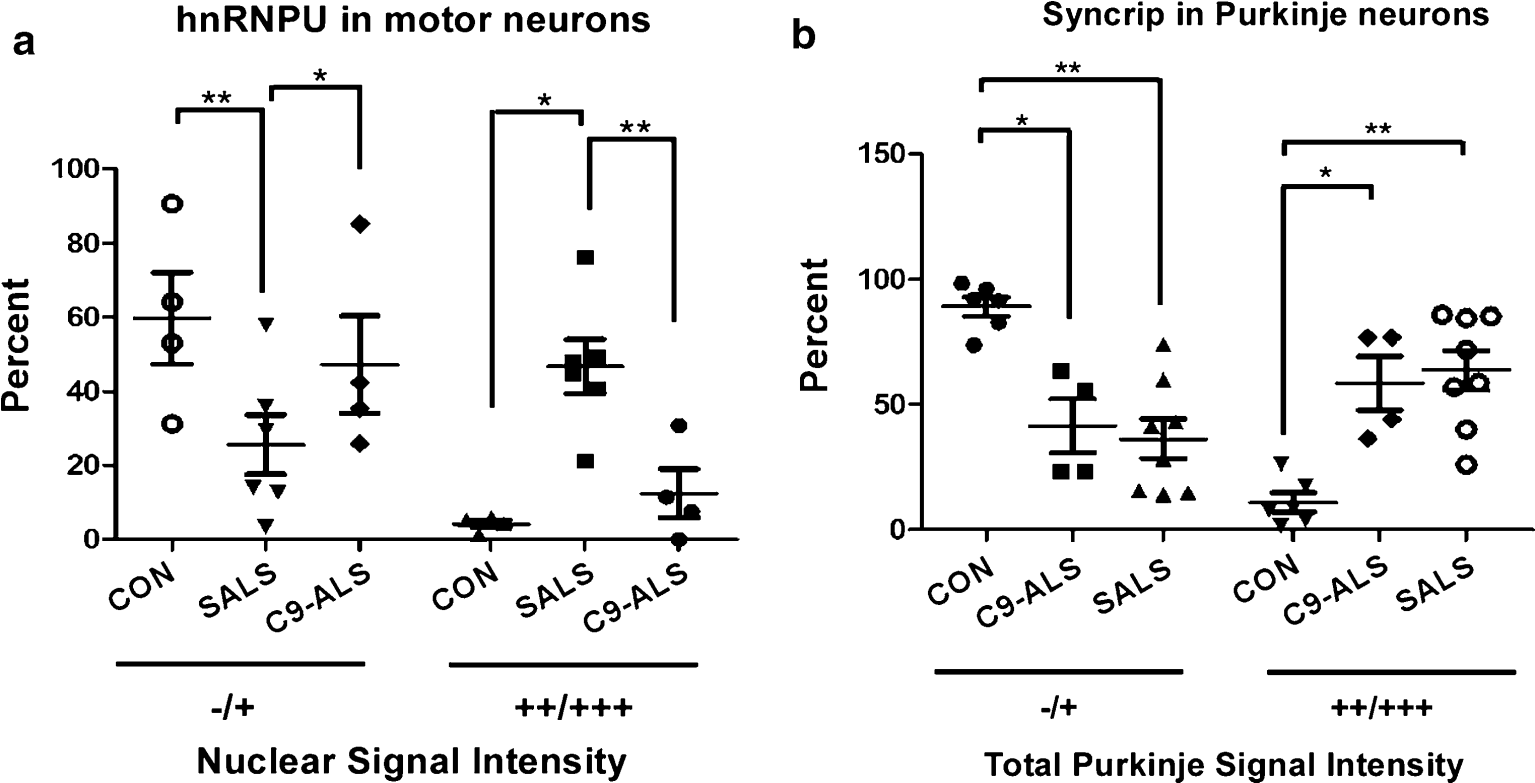
Quantification of hnRNPU and Syncrip staining intensities. **a** hnRNPU motor neuron nuclear staining intensity ranges were measured using the Aperio ImageScope software in lumbar spinal cord sections (see “Materials and methods”). The four intensity ranges (negative, weak, medium and strong) were combined into two categories for ease of viewing, with negative/weak depicted by −/+ and medium/strong depicted by ++/+++ and the results were plotted for controls, C9-ALS and SALS. One-way ANOVA with Bonferroni’s multiple comparison testing demonstrated that for the medium/strong group (++/+++), SALS was significantly different from CON (*p < 0.01), and C9-ALS (**p < 0.01), while C9-ALS and CON were not different from each other. **b** Syncrip staining intensities of Purkinje cells, with categories pooled into negative/weak (−/+) and medium/strong (++/+++). One-way ANOVA shows that for the negative/weak group, CON was significantly different from SALS (*p < 0.01) and from C9-SALS (**p < 0.001); while for the medium/strong group, CON was statistically different from the C9-ALS group (**p < 0.01), and from the SALS group (*p < 0.001). Values depicted are means ± SEM (standard error of the mean)

Syncrip and RBMS3 both showed variable IHC staining patterns in spinal motor neurons of control as well as SALS and C9-ALS, with no significant differences detected between the different subject groups (Suppl. Figure 1a–b). In the cerebellum, Syncrip immunoreactivity was significantly increased in ALS versus controls (Fig. 2a). Purkinje cells displayed weak Syncrip immunostaining in control subjects (89% of neurons displayed negative-to-weak immunoreactivity; Fig. 3b), while neuronal staining was significantly increased in both the C9-ALS and SALS groups (58 and 64% of neurons were associated with medium-to-strong Syncrip staining in each subject group, respectively). Syncrip staining in SALS tend to be nuclear, while many C9-ALS cases showed more diffuse cytoplasmic immunoreactivity.

We detected no differences of RBMS3 staining in cerebellar Purkinje or granule cells in ALS (Fig. 2c). However, there was increased RBMS3 staining in cerebellar interneurons in the molecular layer as well as the granular and Purkinje layers of ALS cases. RBMS3-positive interneurons were found in SALS and C9-ALS cases co-labeled for the interneuron marker calretinin (Fig. 5a). Based on location and cellular morphology of these interneurons, these were identified as Lugaro cells, characterized by dendrites running parallel to the Purkinje cell layer [22], as well as unipolar brush cells, (calretinin-positive parvalbumin-negative, located in the granular layer), Golgi cells (calretinin and parvalbumin-positive cells) and basket or stellate cells (calretinin negative, parvalbumin positive; Fig. 5a; [18]).

**Fig. 4:**
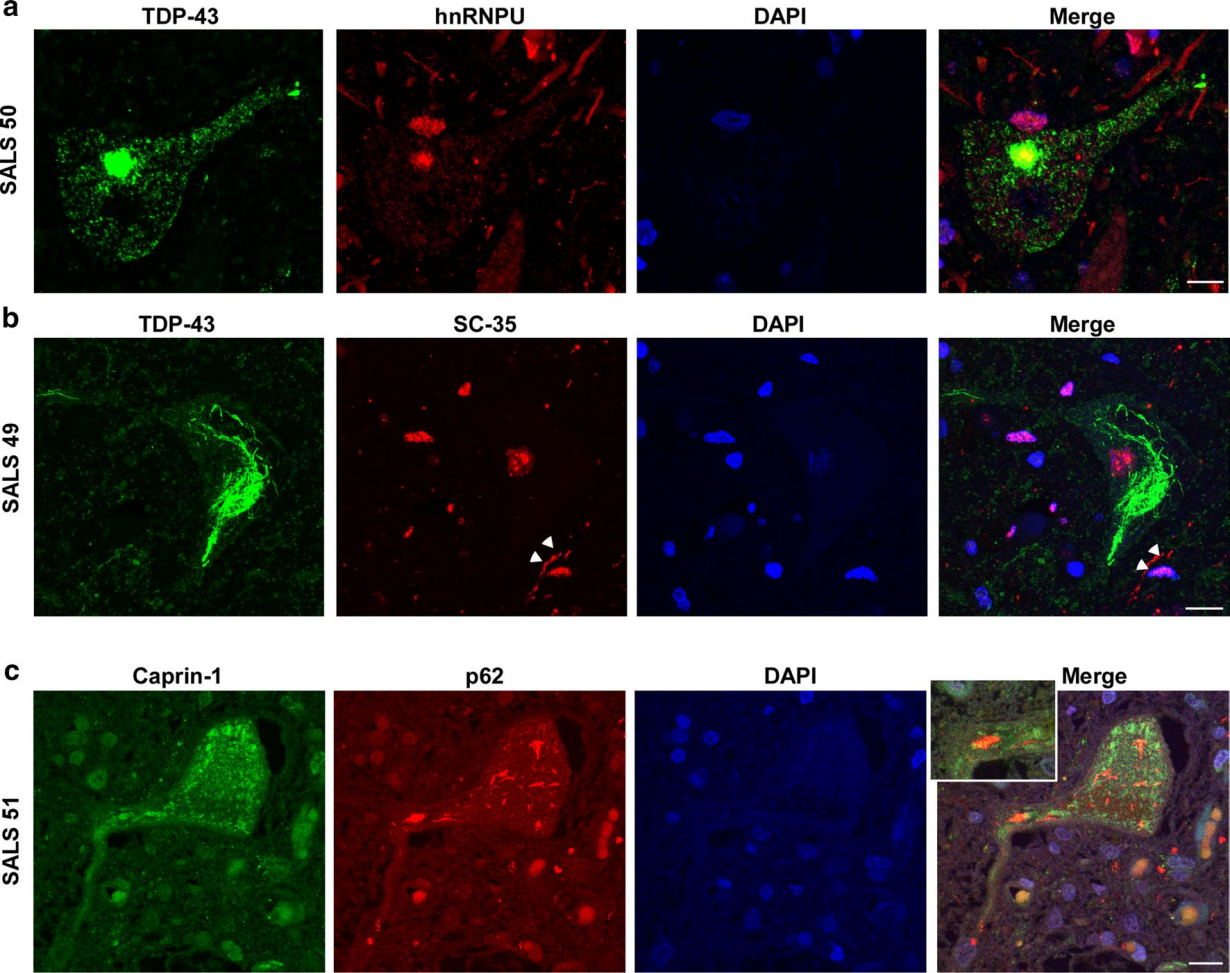
Co-localization of top-ranked RBPs with TDP-43 or p62. Lumbar spinal cords sections from SALS sections were co-stained with **a** hnRNPU and TDP-43, **b** SC-35 and TDP-43, or **c** Caprin-1 and p62. Nuclei were co-stained with DAPI and images were captured on a confocal microscope at ×63 magnification. Scale bars represent 10 μm

The nuclear speckle protein SRSF2/SC-35 displayed a nuclear punctate staining pattern in all subjects, with some ALS cases showing strong and larger speckles, while one SALS case also exhibited cytoplasmic SC-35 inclusions that did not co-localize with TDP-43 (Figs. 1b, 4b, white arrowheads). Occasional neuropil staining for SC-35 was also observed for ALS, as well as increased glial immunoreactivity. In addition, SC-35 positive cytoplasmic tangle-like inclusions and neuropil staining were detected in the frontal cortex of C9-ALS and frontotemporal lobar degeneration (FTLD) (FTLD-tau and FTLD-TDP) cases, but not SALS or controls (Suppl. Figure 3a). SC-35 IHC levels were reduced in the frontal cortex of three SALS compared to three controls, though further studies are required to validate these findings. In the cerebellum, strong SC-35 immunostaining was associated with large nuclear speckles in Purkinje cells of ALS versus controls (Suppl. Figure 2b).

No significant differences in hnRNPH2 localization or staining intensity were observed in either SALS or C9-ALS cerebellum and spinal cords when compared to controls (Suppl. Figures 1c and 2a).

**Fig. 5:**
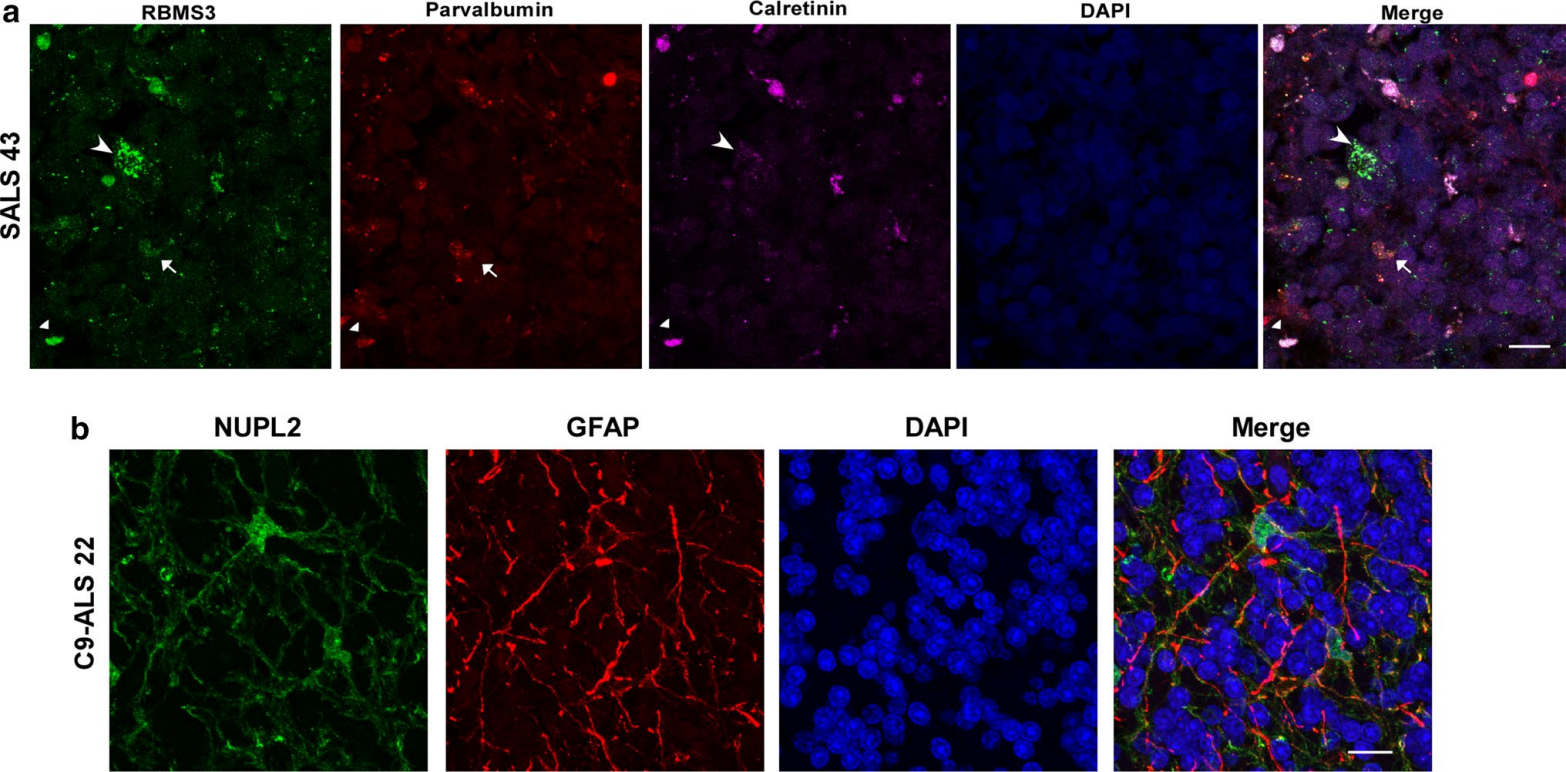
Cell type determination of RBMS3 and NUPL2 positive cells in the cerebellum by confocal microscopy. **a** Cerebellar sections from SALS were co-labeled with RBMS3, parvalbumin or calretinin to identify interneurons. **b** Double-label confocal microscopy of NUPL2 and GFAP to identify astrocytes. Large arrowheads in **a** depict calretinin-positive, parvalbumin-negative interneurons (possibly unipolar brush cells), small arrowheads point to calretinin-positive, parvalbumin-positive (potentially Golgi cells), while small arrows point to calretinin-negative, parvalbumin-positive interneurons (possible basket or stellate cells). Scale bars represent 10 μm

The nucleoporin-like protein NUPL2, ranked 7 by Watson, showed variable immunoreactivity in spinal cord motor neurons (Suppl. Figure 1d), with cytoplasmic puncta detected in most ALS cases and prominent nucleolar staining in two ALS cases. Strong astrocytic NUPL2 staining was also detected in four out of five ALS cases, and one out of two C9-ALS cases. However, no consistent neuronal IHC pattern was noted that differentiated ALS from controls. In the cerebellum, NUPL2 was also localized to astrocytes in SALS but not C9-ALS or control cases (Fig. 2d and Suppl. Figure 3b). In four out of five SALS cerebellum, but only one out of four C9-ALS cases, moderate-to-strong NUPL2 staining was observed in the granular layer and white matter, as well as in fiber tracts of the molecular layer (Suppl. Figure 3b). NUPL2 co-localized with GFAP in the cerebellum, indicating that NUPL2-positive cells were indeed cerebellar astrocytes (Fig. 5b). Purkinje, granule neurons and interneurons were typically negative for NUPL2.

Caprin-1 localizes to cytoplasmic granules in control motor neurons and Purkinje cells as previously described (Figs. 1c, 2b; [47]). ALS motor neurons and Purkinje cells exhibited larger and strong Caprin-1-positive cytoplasmic granules (Figs. 1c, 2b). These large cytoplasmic granules occasionally co-localized with p62 (Fig. 4c) indicating that some of these granules were cytoplasmic inclusions. In addition, most SALS cases displayed Caprin-1 redistribution to the nucleolus, while none of the control or C9-ALS cases had any nuclear or nucleolar Caprin-1 immunostaining.

We detected weak RBM6 immunoreactivity in spinal cord motor neurons, and none in the nucleus of control spinal motor neurons or Purkinje cells (Fig. 1d and Suppl. Figure 2d). ALS cases displayed increased RBM6 spinal cord motor neuron nucleolar immunoreactivity. Similar to Caprin-1, this phenotype was exclusive to the SALS group and not observed in C9-ALS. No differences across the subject groups were seen for RBM6 in the cerebellum (Suppl. Figure 2d).

We performed a similar IHC analysis in the spinal cord and cerebellum with three RBPs from the bottom of the IBM Watson-ranked candidate list: QTRT1 (ranked at position 1467), WARS (position 1463) and NARS (ranked at 1453), each chosen for commercial availability of specific antibodies. No differences were seen for these proteins in either the cerebellum or spinal cord (Suppl. Figure 4), indicating that RBPs near the bottom of the Watson ranking are not altered in ALS.

### Gene expression analysis of top-ranked IBM Watson RBPs also validates alterations in ALS

**Fig. 6:**
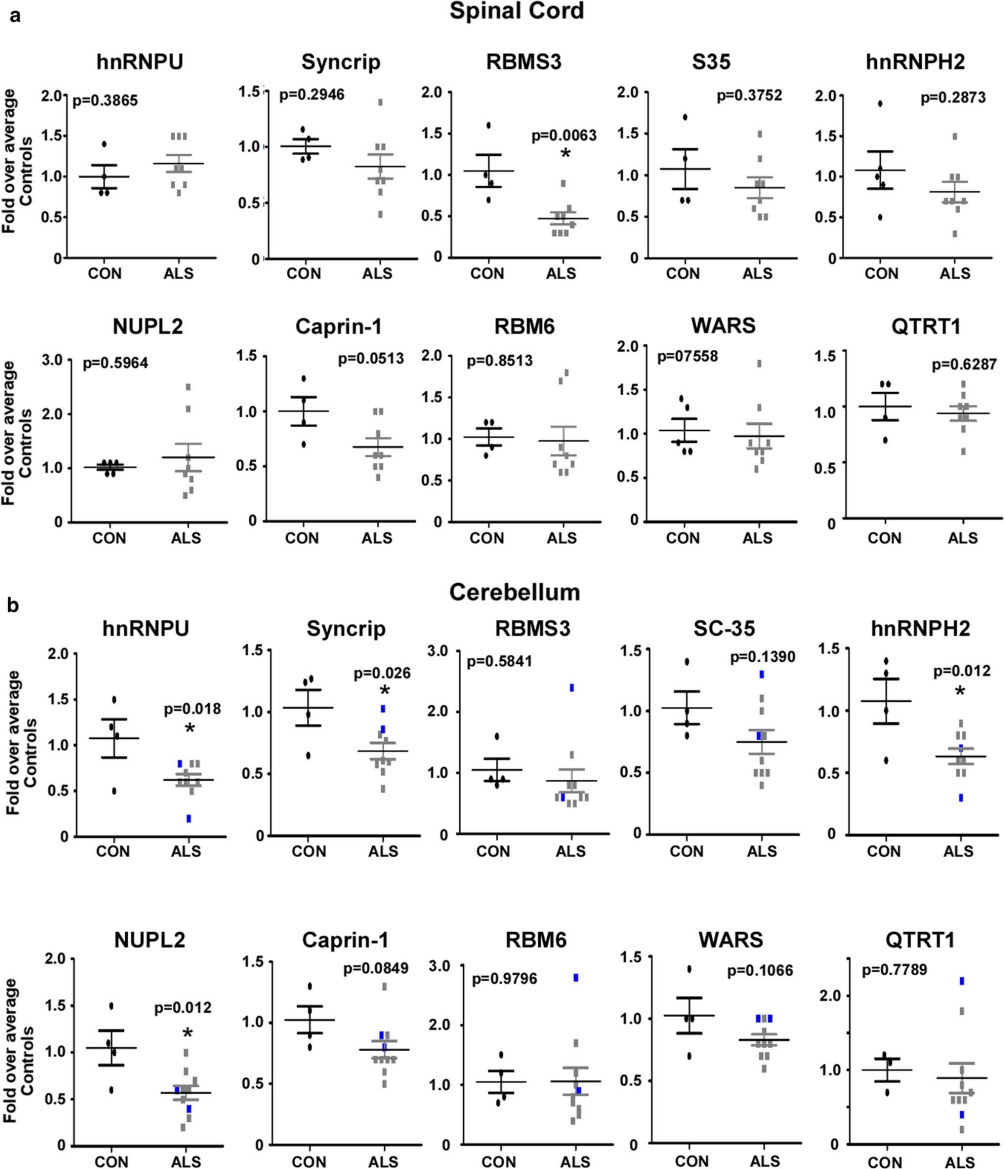
Gene expression of IBM Watson RBPs in spinal cord and cerebellum. **a** RNA from 4–5 control and 8 SALS spinal cords were extracted, cDNA was made and real-time PCR was performed for the IBM Watson top and bottom-ranked RBPs. **b** Cerebellum tissue from 4 controls, 7–8 SALS, and 2 C9-ALS (shown in blue) were used for real-time PCR. Individual values depicted are average of three experimental replicates, and mean ± SEM are shown. Significance is indicated by stars and *p* values are listed in each plot

To further confirm IBM Watson’s top-ranked RBPs, we measured their transcriptional levels in total RNA from spinal cord and cerebellum of ALS patients and non-neurologic disease controls (Fig. 6). Of the eight top-ten RBPs examined, only RBMS3 showed significant decreases in transcriptional levels in ALS spinal cord when compared to controls (Fig. 6a). When we performed a similar analysis in cerebellar tissues, four out of these eight RBPs, hnRNPU, Syncrip, hnRNPH2 and NUPL2 were significantly downregulated in ALS compared to controls (Fig. 6b). None of the bottom-ranked proteins showed significant changes in gene expression in spinal cord or cerebellum (Fig. 6a, b).

**Fig. 7:**
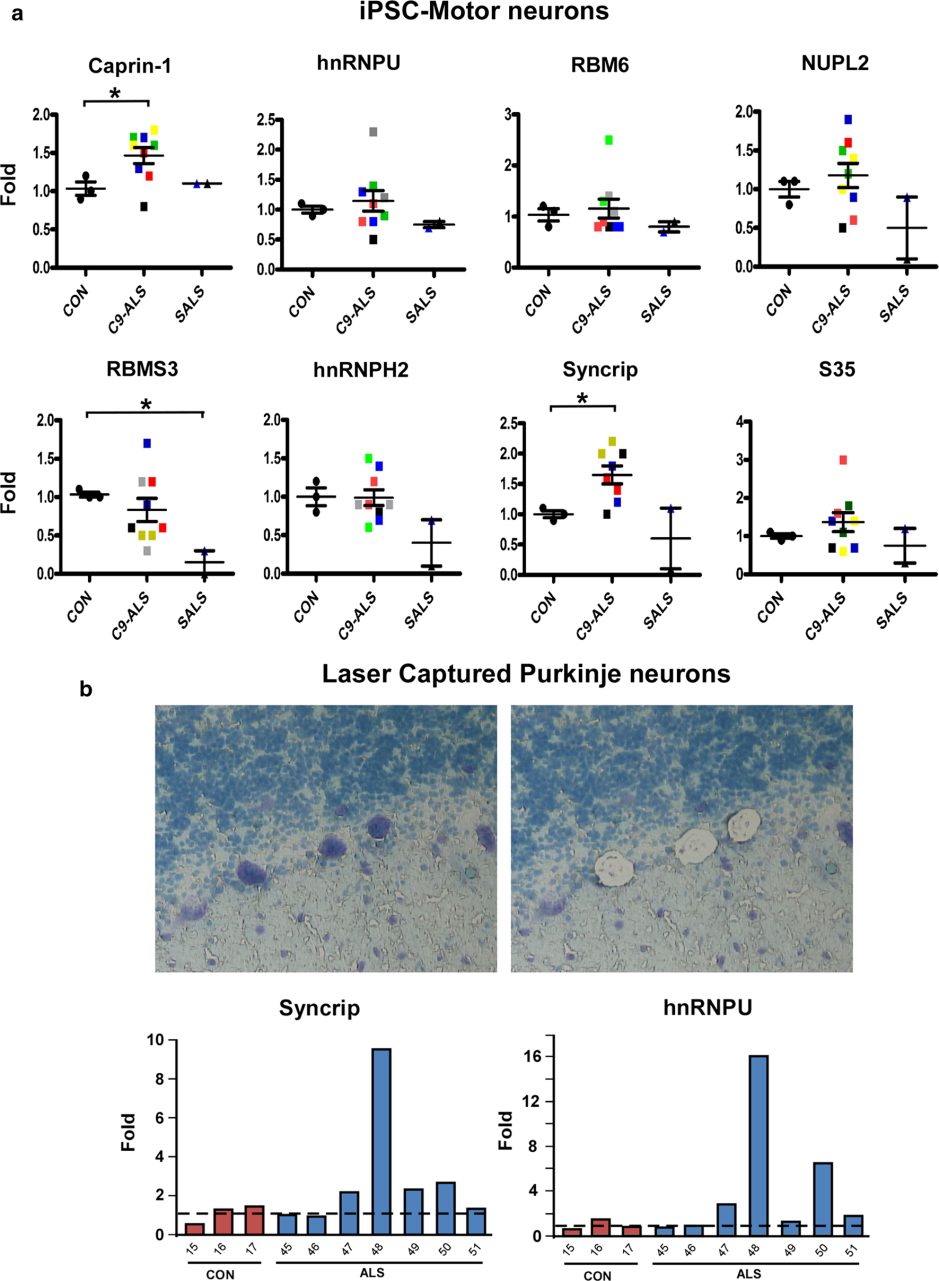
Gene expression of IBM Watson proteins in iPSC-MN and laser-captured Purkinje cells. **a** iPSC-derived motor neurons were differentiated for 45–60 days, RNA extracted and real-time PCR was performed for IBM Watson-ranked RBPs. 3 separate control iPSC lines, 5 different C9-ALS lines (2 independent differentiations of 4 lines C9-ALS 2–5, and one differentiation of line C9-ALS1), and two SALS lines were used. The different colors depict the various lines used, ran in experimental triplicates, and values shown are means ± SEM. Asterisks denote significance, with *p* values of 0.0495 for Caprin-1 (CON vs. C9-ALS), 0.0052 for RBMS3 (CON vs. SALS), and 0.0314 for Syncrip (CON vs. C9-ALS). **b** Frozen cerebellar sections were stained with methyl green pyronin, and at least 250 Purkinje cells were captured from each case, RNA extracted and real-time PCR was performed on each sample. 3 controls and 7 SALS cases were used. Bars represent individual data points calculated from experimental replicates

Since RNA extracted from total spinal cord or cerebellum tissues includes various cell types, we also investigated transcriptional levels of these same RBPs in patient-derived pluripotent stem cells (iPSC) differentiated into motor neurons. We isolated RNA from motor neurons generated from five C9-ALS iPSC lines, two SALS iPSC cell lines, and three control iPSC lines (Fig. 7a). Both Caprin-1 and Syncrip showed significant upregulation in C9-ALS iPSC-MN compared to controls, while RBMS3 showed decreased expression in the two SALS lines compared to controls, recapitulating results obtained in total spinal cord, although a larger sample size is needed to confirm these findings.

To quantify gene expression alterations in cerebellar Purkinje cells, we used laser-capture microscopy (LCM) to isolate individual Purkinje cells from frozen cerebellum sections of ALS and neurologic disease controls (Fig. 7b). Approximately 250 Purkinje cells were isolated per sample to examine transcriptional levels for each candidate RBP. Syncrip was upregulated in four out of seven SALS cases compared to three controls, while hnRNPU was increased in three out of seven SALS cases. None of the other RBPs examined exhibited statistically significant transcriptional changes in ALS-Purkinje cells when compared to controls (data not shown).

### Protein levels of top-ranked IBM Watson-predicted RBPs

**Fig. 8:**
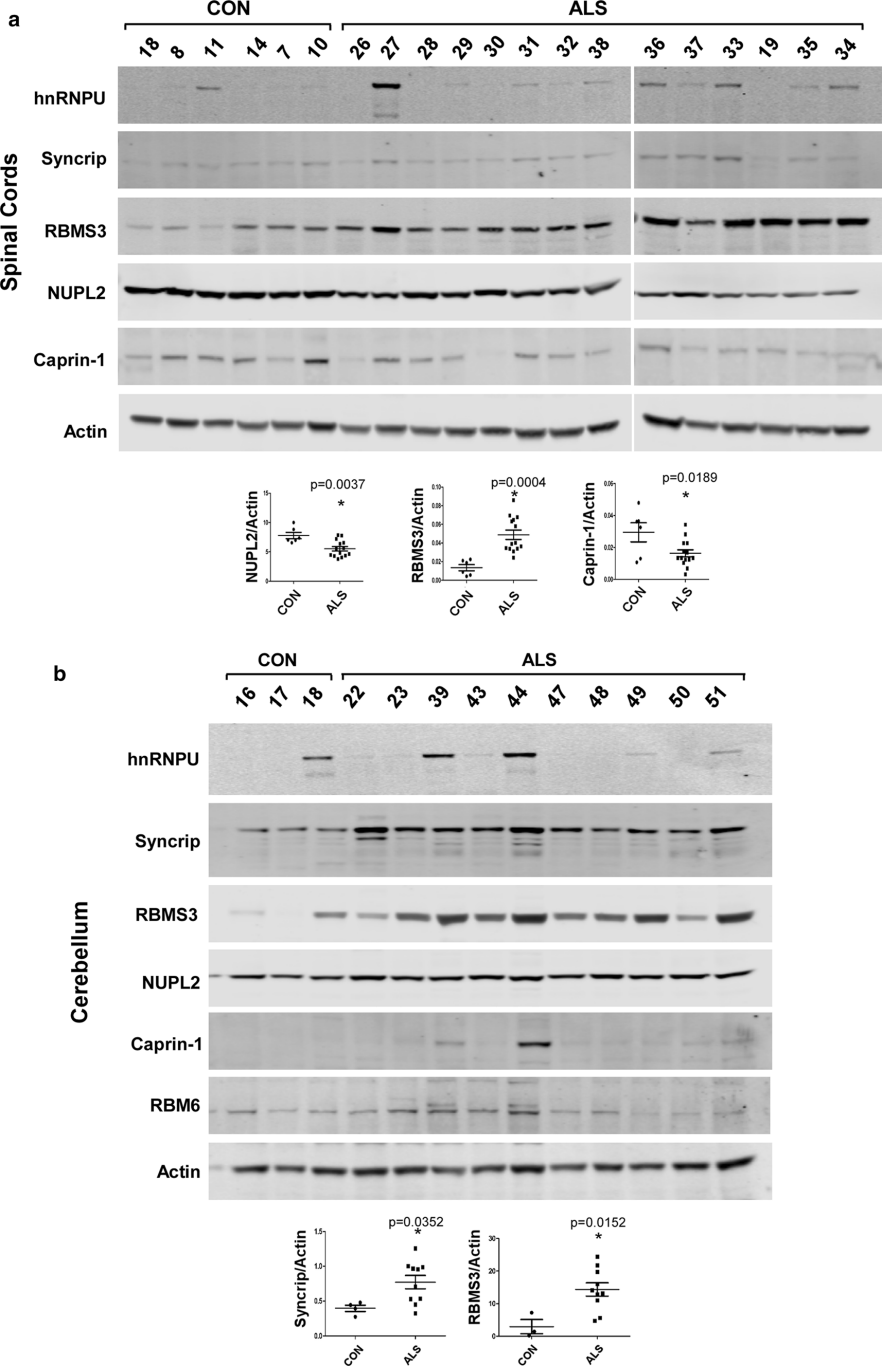
Immunoblot analysis of IBM Watson-ranked proteins in spinal cord and cerebellum. **a** Immunoblot of protein lysates prepared from 6 control and 14 SALS spinal cord sample. **b** Immunoblot of cerebellum protein lysates from 4 control, 8 SALS and 2 C9-ALS (#22 and 23) cases. Graphs and statistical analysis are shown for proteins that are significantly altered in ALS when compared to controls

We next investigated protein levels of IBM Watson-ranked RBPs by western blot analysis in cerebellum and spinal cord tissues. Protein levels of hnRNPU were increased in many ALS samples compared to non-neurologic disease controls in both cerebellum and spinal cord, mirroring increases observed by IHC in spinal cord (Fig. 8a, b and Table 5). However, results failed to reach statistical significance due to the large sample-to-sample variability within the ALS group. Syncrip levels were significantly increased in ALS cerebellum, but not spinal cord, again reflecting changes seen by IHC (Fig. 8a, b and Table 5). RBMS3 had significantly increased protein expression levels in ALS cerebellum and spinal cords compared to non-neurological disease controls, recapitulating observed increased cerebellar interneuronal staining (Fig. 8a, b and Table 5). NUPL2 was significantly decreased in ALS spinal cords, in apparent contradiction to its increased IHC in ALS spinal cord and cerebellum. No significant changes were seen for negative controls NARS or WARS (data not shown).

## Discussion

The use of machine learning algorithms and other artificial intelligence technologies is impacting medical care and research, and offers new approaches to analyze complex biological datasets to provide new insight into human disease. We used IBM Watson to screen and rank order RBPs to identify additional RBPs involved in ALS. Using a set of 11 RBPs with known mutations that cause ALS and a candidate set comprising 1467 RBPs with at least one published abstract up to the end of 2015, IBM Watson text mined published abstracts in the literature, and ranked all candidate RBPs by their semantic similarity to the known RBPs with ALS-causing mutations. We then validated the top-ten candidates for potential alterations in ALS using a combination of immunohistochemistry, RNA and protein analysis in tissues from ALS and non-neurologic disease controls, and RNA analysis of iPSC-derived motor neurons. These results are summarized in Table 5. The top-three ranked RBPs (hnRNPU, Syncrip and RBMS3) exhibited alterations in ALS by multiple methods, including protein distribution, RNA and protein levels in ALS compared to controls. Two other RBPs ranked in the top-ten by Watson, NUPL2 and Caprin-1, also exhibited alterations by multiple validation methods (Table 5). As noted above, Caprin-1, subsequent to our Watson analysis, was shown to localize to TDP-43 and FUS positive inclusions in ALS patients with TDP-43 or FUS mutations [4]. Our criteria for successful validation were significant RBP alterations in more than one assay. Therefore, both hnRNPH2 and RBM6 did not pass our validation criteria; whereas the five other top-ten Watson-ranked RBPs did pass our validation criteria. This top-ten list also included three other RBPs that were previously associated with ALS (RBM45, SC-35 and MTHFSD) but have no known mutations linked to familial forms of ALS. Overall, eight of the top ten ranked RBPs were altered in ALS. All RBPs tested from the bottom of the IBM Watson list showed no alterations in ALS.

One question is whether Watson could have randomly rank ordered all RBPs to generate a top-ten list that would fulfill our validation criteria. The actual number of RBPs altered in ALS is not known, so we cannot precisely determine the accuracy of Watson predictions at ranking RBPs linked to ALS. Instead, we used Fisher’s exact test to calculate the probability of Watson correctly identifying eight of the top ten RBPs as altered in ALS. Using results from the LOO analysis, we could assume that 5% of the total RBPs used in this study (73 out of 1467 RBPs) are altered in ALS. Using this assumption, the Fisher’s exact test generates *p* = 1.07 × 10^−9^ for Watson correctly predicting eight of the top ten to be altered in ALS. If we make a very conservative estimate and assume that 20% of all RBPs (293 out of 1467) are altered, then the significance of the Watson predictions is *p* = 7.21 × 10^−5^. Therefore, the probability that Watson randomly selected RBPs and correctly predicted eight of the top ten by chance is quite low. While we could not perform extensive validation of all Watson RBP predictions due to time and cost, we focused validation efforts on the top ten and selected RBPs at the bottom of the list for which there were commercially available antibodies. These negative controls are all involved in tRNA metabolism, which Watson semantically ranked as most dissimilar to the known ALS-RBPs that function predominately in mRNA metabolism. Other RBPs that function in tRNA metabolism were also ranked near the bottom of the list, suggesting that this pathway does not significantly contribute to ALS.

Even though hnRNPU, Caprin-1, SRSF2 and Syncrip can be found within supplemental tables of unbiased proteomic screens for potential interacting proteins of TDP-43, FUS and Ataxin2 (Table 4), these supplemental data were not available to Watson’s analysis that focused on published abstracts. Such global proteomic analyses typically generate hundreds of potential hits, though without further validation studies these remain putative protein interactions and it is difficult to rank order which candidate proteins should be further explored. The use of computer-based approaches such as IBM Watson to mine text and/or data can focus subsequent experimental validation efforts to those putative interacting proteins highly ranked by Watson.

The top-ranked RBP, hnRNPU co-localized to cytoplasmic TDP-43 positive inclusions and showed significant protein increases in motor neurons, as well as in cerebellum and spinal cord protein lysates from ALS compared to non-neurologic disease controls. Yet, hnRNPU transcript was significantly downregulated in ALS cerebellum. Similarly, Syncrip also showed altered subcellular distribution and increased protein expression in the cerebellum, along with modest increases in protein levels in ALS spinal cord, yet its RNA transcript was downregulated in ALS cerebellum. However, Syncrip mRNA expression was increased in C9-iPSC-derived motor neurons, suggesting the analysis of total tissue extracts may mask changes within individual cell types. Nevertheless, we did note discordance between protein and RNA expression levels of multiple RBPs within the same tissue, similar to prior results described in aging human brain [55], and perhaps attributable to pathological changes in mRNA translation or microRNA regulation that occur in ALS.

While the use of IBM Watson in ALS and the neurosciences was novel and we successfully identified new RBPs that exhibit alterations in ALS, there remain limitations to our approach. Watson relies on gene annotations of the published literature for its text-based analysis. In our study, hnRNPH2, ranked number 6 by Watson, exhibited few alterations in ALS (Table 5), but was found to have a similar annotation nomenclature within the published literature as hnRNPH/hnRNPH1, which has been linked to ALS [11]. This example of common annotations likely led Watson to infer that hnRNPH2 was equivalent to hnRNPH and hnRNPH1, generating a false positive in our analysis. While we used a rigorous disambiguation of gene annotations for our study (see Supplemental Methods), continued work on gene annotations will aid future gene-based studies using IBM Watson. Another limitation of Watson’s analysis is the fact that it is based on semantic similarity to a known set of proteins. For example, DDX58 was identified in 2016 as an RBP altered in ALS tissue [37]. However, in our study Watson ranked DDX58 number 769, making it a false negative result. Since the most common function of DDX58, a cytoplasmic sensor of viral infection, is vastly dissimilar to the function of RBPs used in our known training set, Watson assigned DDX58 a low score in its model. The addition of neuroscience-specific knowledge and complex biologic datasets generated by neuroscience laboratories into the IBM Watson system will benefit future Watson-based neuroscience studies.

It is noteworthy that from the transcriptional analysis of RBP changes in ALS tissues, more changes were observed in cerebellum when compared to spinal cord; four genes were significantly altered in ALS vs control in cerebellum, while only one gene (RBMS3) was altered in ALS spinal cord. Such a trend towards more robust transcriptomic changes in cerebellum compared to other brain regions was recently reported by Prudencio et al. [44], when comparing cerebellum to frontal cortex of C9-ALS and SALS by RNA-sequencing analysis.

Cerebellar involvement in ALS has recently gained acceptance by the field. Cerebellar atrophy, namely loss of Purkinje cells in the cerebellar vermis region, was reported in ALS cases with ATXN2 gene expansions, but not C9-ALS or SALS cases [51]. C9-ALS cases are associated with p62-positive, phospho-TDP43 negative cytoplasmic inclusions in the granular and molecular layers, as well as in Purkinje cells of the cerebellum [2]. Structural changes in ALS cerebellar integrity have been demonstrated as white and grey matter alterations by 3D-MRI [29]. More recently, similar imaging analyses have shown ALS cerebellar atrophy in the inferior cerebellum and vermis, areas typically associated with motor tasks, while the cerebellum of ALS-bvFTD subjects show atrophy both in the superior and inferior cerebellum [50]. One RBP identified by Watson and validated as significantly altered in ALS cerebellum was NUPL2. NUPL2 specifically marked ALS astrocytes in the cerebellum and spinal cord. A prior study in transgenic SOD1-G93A mice identified phospho-ERK in cerebellar astrocytes, highlighting ALS-specific changes within astrocytes in the cerebellum [9]. NUPL2 is a nucleoporin-like protein that regulates nuclear export of protein and mRNA, yet can localize to both the nucleus and the cytoplasm. NUPL2 was also contained in the cytoplasm of control spinal motor neurons, but in many ALS cases, NUPL2 was redistributed to the nucleolus of motor neurons, although the significance of this redistribution is unknown.

A novel ALS phenotype is the increased expression of RBMS3 and RBM6 in cerebellar interneurons. Spinal cord and cortical interneuron alterations in GABA-A receptor and parvalbumin levels have been reported in ALS patients and animal models of ALS [38, 42, 43]. In addition, reduced GABAergic transmission, hyperexcitability and excitotoxicity of layer 5 pyramidal neurons was observed in TDP43-A315T mice, while a low copy-number model of SOD1-G93A mice showed reduced GABAergic and glycinergic spinal interneurons, along with interneuron ubiquitinated inclusions prior to disease onset [26, 57]. Our results thus highlight alterations of interneurons in ALS.

Whole exome sequencing recently identified NEK1 as a risk factor for ALS [31], though we were unable to identify any genetic alterations linked to ALS for our Watson top-ten RBPs using publically available exome sequencing data. Additional genetic analyses of RBPs ranked in the top 5–10% of the Watson list is necessary to determine if Watson can use its algorithms to identify new gene mutations linked to ALS using only comparisons to the known RBPs with mutations that cause ALS. Although Syncrip did show a trend for a distinct phenotype in the cerebellum of C9-ALS compared to SALS patients, further studies are needed to expand the group size and include additional familial forms of ALS to confirm these findings.

In conclusion, we used IBM Watson to leverage published literature and semantic similarity to known ALS-RBPs find additional RBPs altered in ALS. This approach is a great addition to the usual candidate screening approaches, and can be used to sieve through hundreds of potential hits generated from -omics based experimental approaches and make literature-based rank-ordering of targets worthy of further validation studies. IBM Watson identified and we validated alterations in five RBPs out of seven RBPs previously unlinked to ALS, including novel alterations of RBMS3 within cerebellar interneurons. The top-ten list included three other RBPs that were previously associated with ALS (RBM45, SC-35 and MTHFSD), while RBPs ranked near the bottom of the list failed to exhibit changes in ALS. Further studies are required to determine if RBPs ranked high by Watson contain any genetic alterations that can be linked to ALS. The continued and future use of IBM Watson and other machine learning computing tools will likely accelerate scientific discovery in ALS and other complex neurological disorders.

## Electronic supplementary material

Below is the link to the electronic supplementary material.

Supplementary material 1 (DOCX 110 kb)

Supplementary material 2 (TIFF 14290 kb)

Supplementary material 3 (TIFF 14283 kb)

Supplementary material 4 (TIFF 11712 kb)

Supplementary material 5 (TIFF 13606 kb)

Supplementary material 6 (XLSX 1361 kb)

Supplementary material 7 (XLSX 407 kb)

Supplementary material 8 (XLSX 23618 kb)

Supplementary material 9 (PDF 616 kb) This file includes a pseudo-code for the algorithm published in reference 48, as well as a detailed explanation of how this code was used to generate the results presented in this paper
