# Supplementary material for: Artificial intelligence in neurodegenerative disease research: use of IBM Watson to identify additional RNA-binding proteins altered in amyotrophic lateral sclerosis
Source: Acta Neuropathol. 2017 Nov 13;135(2):227–47. doi: 10.1007/s00401-017-1785-8 (PMC5773659; doi:10.1007/s00401-017-1785-8)
Supplement: Supplementary file 1 — Supplementary material 1 (DOCX 110 kb) [file 401_2017_1785_MOESM1_ESM.docx]

Artificial Intelligence in neurodegenerative disease research: Use of IBM Watson to identify additional RNA binding proteins altered in amyotrophic lateral sclerosis

**Authors:** Nadine Bakkar^1^, Tina Kovalik^1^, Ileana Lorenzini^1^, Scott Spangler^2^, Alix Lacoste^3^, Kyle Sponaugle^1^, Philip Ferrante^1^, Elenee Argentinis^3^, Rita Sattler^1^, Robert Bowser^1^*

**Journal Name:** Acta Neuropathologica

**Affiliations:**

^1^Department of Neurobiology, Barrow Neurological Institute, Phoenix, AZ USA.

^2^IBM Research – Almaden, San Jose, CA USA.

^3^IBM Watson Health, New York, NY USA.

*To whom correspondence should be addressed: Robert Bowser, Department of Neurobiology, Barrow Neurological Institute, 350 W Thomas Road, Phoenix, AZ 85213. Email: [Robert.bowser@dignityhealth.org](mailto:Robert.bowser@dignityhealth.org).

**Table S1. IBM Watson Rank of candidate RBPs in Prospective Analysis**

| **Protein** | **# Abstracts** | **Score (GD)** | **Rank (GD)** |
| --- | --- | --- | --- |
| **HNRNPU** | 318 | 0.00291413 | 1 |
| **SYNCRIP** | 79 | 0.0027466 | 2 |
| RBM45 | 9 | 0.00267976 | 3 |
| **RBMS3** | 30 | 0.00249365 | 4 |
| **SRSF2** | 620 | 0.0024586 | 5 |
| **HNRNPH2** | 21 | 0.00225497 | 6 |
| **NUPL2** | 74 | 0.00215157 | 7 |
| **CAPRIN1** | 41 | 0.00210949 | 8 |
| **RBM6** | 117 | 0.00191485 | 9 |
| MTHFSD | 3 | 0.00191011 | 10 |
| RBMS1 | 158 | 0.00186319 | 11 |
| HNRNPAB | 79 | 0.00184504 | 12 |
| HNRNPH1 | 116 | 0.00184193 | 13 |
| HNRNPM | 66 | 0.00184166 | 14 |
| EEF1D | 23 | 0.00183648 | 15 |
| POLDIP3 | 31 | 0.00154007 | 16 |
| RBM14 | 13 | 0.00153667 | 17 |
| HNRNPA3 | 47 | 0.00153418 | 18 |
| IGHMBP2 | 144 | 0.00153138 | 19 |
| HNRNPK | 375 | 0.00149436 | 20 |
| RBMX | 65 | 0.00148401 | 21 |
| SRSF4 | 49 | 0.00148067 | 22 |
| DDX39A | 51 | 0.00147497 | 23 |
| GRSF1 | 17 | 0.00144837 | 24 |
| SAFB | 436 | 0.0014132 | 25 |
| API5 | 120 | 0.00135777 | 26 |
| PRDX1 | 762 | 0.001357 | 27 |
| ATXN1 | 774 | 0.00115847 | 28 |
| HNRNPDL | 2 | 0.00115317 | 29 |
| TNPO2 | 22 | 0.00115096 | 30 |
| DYNC1H1 | 80 | 0.00115018 | 31 |
| NOP56 | 61 | 0.00114419 | 32 |
| NXF5 | 13 | 0.00113782 | 33 |
| HNRNPF | 78 | 0.00113674 | 34 |
| RNASEH2A | 43 | 0.00113498 | 35 |
| SRSF9 | 49 | 0.0011286 | 36 |
| TRA2B | 61 | 0.00111584 | 37 |
| SRSF10 | 63 | 0.00111377 | 38 |
| SRSF7 | 79 | 0.00111054 | 39 |
| SRRM2 | 22 | 0.00110588 | 40 |
| PTBP3 | 36 | 0.00110377 | 41 |
| IPO5 | 90 | 0.00109834 | 42 |
| BICC1 | 50 | 0.00109507 | 43 |
| DDX46 | 72 | 0.00109197 | 44 |
| YTHDC1 | 22 | 0.00109032 | 45 |
| ZFP36L2 | 93 | 0.00107897 | 46 |
| DDX27 | 33 | 0.00107283 | 47 |
| SF3B4 | 142 | 0.0010705 | 48 |
| KIN | 60 | 0.00106519 | 49 |
| SKIV2L | 71 | 0.00106179 | 50 |
| TSR1 | 56 | 0.00105451 | 51 |
| CTU1 | 84 | 0.00105268 | 52 |
| LRRFIP1 | 50 | 0.00102366 | 53 |
| HNRNPC | 18 | 7.78E-04 | 54 |
| RAVER2 | 6 | 7.76E-04 | 55 |
| HNRNPR | 27 | 7.76E-04 | 56 |
| RBM42 | 3 | 7.75E-04 | 57 |
| HNRNPA0 | 17 | 7.75E-04 | 58 |
| ATXN1L | 9 | 7.73E-04 | 59 |
| APTX | 145 | 7.73E-04 | 60 |
| PCBP1 | 136 | 7.73E-04 | 61 |
| RTCB | 64 | 7.72E-04 | 62 |
| SMN2 | 567 | 7.72E-04 | 63 |
| RNASEH2B | 29 | 7.72E-04 | 64 |
| RBMXL2 | 9 | 7.71E-04 | 65 |
| EWSR1 | 1000 | 7.71E-04 | 66 |
| HNRNPH3 | 85 | 7.71E-04 | 67 |
| ZNF638 | 11 | 7.71E-04 | 68 |
| TSEN2 | 8 | 7.71E-04 | 69 |
| HNRNPL | 124 | 7.70E-04 | 70 |
| TSEN54 | 28 | 7.70E-04 | 71 |
| CWF19L1 | 3 | 7.69E-04 | 72 |
| EIF2B3 | 24 | 7.69E-04 | 73 |
| TEX13A | 2 | 7.69E-04 | 74 |
| THOC5 | 34 | 7.68E-04 | 75 |
| NUP153 | 188 | 7.68E-04 | 76 |
| RBM15B | 5 | 7.68E-04 | 77 |
| FYTTD1 | 7 | 7.67E-04 | 78 |
| ZRSR2 | 44 | 7.66E-04 | 79 |
| XPO1 | 1000 | 7.66E-04 | 80 |
| NSUN3 | 2 | 7.65E-04 | 81 |
| ZC3H14 | 26 | 7.64E-04 | 82 |
| SON | 85 | 7.64E-04 | 83 |
| KPNB1 | 38 | 7.64E-04 | 84 |
| DDX19B | 42 | 7.63E-04 | 85 |
| RAE1 | 217 | 7.63E-04 | 86 |
| C12orf65 | 14 | 7.62E-04 | 87 |
| ENDOU | 406 | 7.62E-04 | 88 |
| THOC2 | 79 | 7.61E-04 | 89 |
| RNPS1 | 605 | 7.61E-04 | 90 |
| THRAP3 | 22 | 7.51E-04 | 91 |
| AFF2 | 224 | 7.50E-04 | 92 |
| DDX52 | 26 | 7.42E-04 | 93 |
| TDRKH | 29 | 7.38E-04 | 94 |
| SPEN | 54 | 7.31E-04 | 95 |
| ZFR | 43 | 7.29E-04 | 96 |
| RPL10A | 59 | 6.41E-04 | 97 |
| NONO | 239 | 3.98E-04 | 98 |
| PCBP3 | 9 | 3.97E-04 | 99 |
| PTBP1 | 825 | 3.96E-04 | 100 |
| DAZAP1 | 30 | 3.96E-04 | 101 |
| RAVER1 | 18 | 3.95E-04 | 102 |
| HNRNPD | 341 | 3.95E-04 | 103 |
| THOC1 | 278 | 3.94E-04 | 104 |
| AKAP8L | 19 | 3.94E-04 | 105 |
| G3BP1 | 179 | 3.94E-04 | 106 |
| SARNP | 87 | 3.93E-04 | 107 |
| CAPRIN2 | 16 | 3.93E-04 | 108 |
| RBM11 | 3 | 3.93E-04 | 109 |
| AGFG1 | 1000 | 3.93E-04 | 110 |
| NSUN5 | 112 | 3.92E-04 | 111 |
| NOP2 | 436 | 3.92E-04 | 112 |
| HNRNPUL1 | 24 | 3.92E-04 | 113 |
| AKAP8 | 50 | 3.92E-04 | 114 |
| PCBP2 | 133 | 3.92E-04 | 115 |
| ALYREF | 360 | 3.91E-04 | 116 |
| PQBP1 | 261 | 3.91E-04 | 117 |
| SMN1 | 1000 | 3.91E-04 | 118 |
| ZC3HAV1L | 1 | 3.91E-04 | 119 |
| ZC3H7B | 7 | 3.91E-04 | 120 |
| RBM4B | 2 | 3.91E-04 | 121 |
| PRR3 | 27 | 3.91E-04 | 122 |
| DUS4L | 19 | 3.91E-04 | 123 |
| WDR83 | 15 | 3.91E-04 | 124 |
| PRKRA | 53 | 3.91E-04 | 125 |
| KHSRP | 325 | 3.91E-04 | 126 |
| SCAF1 | 53 | 3.90E-04 | 127 |
| RNASEH2C | 20 | 3.90E-04 | 128 |
| SRSF1 | 401 | 3.90E-04 | 129 |
| ADARB1 | 369 | 3.90E-04 | 130 |
| SAFB2 | 18 | 3.90E-04 | 131 |
| ASH1L | 185 | 3.90E-04 | 132 |
| GFM1 | 480 | 3.90E-04 | 133 |
| XPO6 | 21 | 3.90E-04 | 134 |
| CHTOP | 19 | 3.90E-04 | 135 |
| THOC3 | 18 | 3.90E-04 | 136 |
| TPR | 1000 | 3.90E-04 | 137 |
| RBM23 | 54 | 3.89E-04 | 138 |
| DDX39B | 92 | 3.89E-04 | 139 |
| TSR2 | 21 | 3.89E-04 | 140 |
| IPO13 | 47 | 3.89E-04 | 141 |
| EIF2B4 | 18 | 3.89E-04 | 142 |
| RANBP2 | 271 | 3.89E-04 | 143 |
| NANOS1 | 158 | 3.89E-04 | 144 |
| ATXN2L | 35 | 3.89E-04 | 145 |
| EXOSC6 | 554 | 3.89E-04 | 146 |
| SWT1 | 8 | 3.89E-04 | 147 |
| DBR1 | 46 | 3.89E-04 | 148 |
| THOC6 | 2 | 3.88E-04 | 149 |
| NUTF2 | 466 | 3.88E-04 | 150 |
| NKRF | 404 | 3.88E-04 | 151 |
| NXF1 | 572 | 3.88E-04 | 152 |
| PABPN1 | 253 | 3.88E-04 | 153 |
| THOC7 | 8 | 3.88E-04 | 154 |
| RBFOX1 | 179 | 3.88E-04 | 155 |
| GNL3L | 70 | 3.88E-04 | 156 |
| INTS2 | 3 | 3.88E-04 | 157 |
| FRG1 | 43 | 3.88E-04 | 158 |
| ZCCHC9 | 2 | 3.88E-04 | 159 |
| RALY | 27 | 3.88E-04 | 160 |
| RAN | 513 | 3.87E-04 | 161 |
| EIF2B2 | 25 | 3.87E-04 | 162 |
| PATL2 | 10 | 3.87E-04 | 163 |
| SRA1 | 771 | 3.87E-04 | 164 |
| ESRP1 | 47 | 3.87E-04 | 165 |
| IPO7 | 88 | 3.87E-04 | 166 |
| INTS3 | 24 | 3.87E-04 | 167 |
| RBM17 | 19 | 3.87E-04 | 168 |
| ZC3H18 | 5 | 3.87E-04 | 169 |
| MTG1 | 257 | 3.87E-04 | 170 |
| RPL26L1 | 1 | 3.87E-04 | 171 |
| LSM12 | 7 | 3.87E-04 | 172 |
| UBAP2 | 3 | 3.87E-04 | 173 |
| NANOS2 | 428 | 3.87E-04 | 174 |
| ZNF239 | 8 | 3.87E-04 | 175 |
| BYSL | 40 | 3.86E-04 | 176 |
| INTS1 | 34 | 3.86E-04 | 177 |
| ZC3HC1 | 9 | 3.86E-04 | 178 |
| TTF2 | 173 | 3.86E-04 | 179 |
| NXF3 | 18 | 3.86E-04 | 180 |
| SNRNP25 | 4 | 3.86E-04 | 181 |
| PHAX | 17 | 3.86E-04 | 182 |
| MRPL45 | 1 | 3.86E-04 | 183 |
| SUGP2 | 2 | 3.86E-04 | 184 |
| SRSF3 | 145 | 3.85E-04 | 185 |
| RBM5 | 416 | 3.85E-04 | 186 |
| RNASE4 | 46 | 3.85E-04 | 187 |
| SNRNP70 | 463 | 3.85E-04 | 188 |
| RNH1 | 955 | 3.85E-04 | 189 |
| RPL26 | 528 | 3.85E-04 | 190 |
| EXOSC3 | 1000 | 3.85E-04 | 191 |
| HARS | 1000 | 3.85E-04 | 192 |
| RNASE6 | 18 | 3.85E-04 | 193 |
| TDRD3 | 8 | 3.84E-04 | 194 |
| ZNF106 | 4 | 3.84E-04 | 195 |
| NMD3 | 34 | 3.84E-04 | 196 |
| PRPF8 | 129 | 3.84E-04 | 197 |
| BRIX1 | 1 | 3.84E-04 | 198 |
| TRNT1 | 16 | 3.84E-04 | 199 |
| RNASE1 | 1000 | 3.84E-04 | 200 |
| GNL1 | 35 | 3.83E-04 | 201 |
| ZCCHC24 | 2 | 3.83E-04 | 202 |
| ZMAT5 | 1 | 3.83E-04 | 203 |
| DHX57 | 1 | 3.83E-04 | 204 |
| RPS3A | 152 | 3.83E-04 | 205 |
| SNUPN | 53 | 3.83E-04 | 206 |
| ZC3H7A | 5 | 3.83E-04 | 207 |
| GARS | 425 | 3.82E-04 | 208 |
| EIF2B1 | 407 | 3.82E-04 | 209 |
| DDX53 | 823 | 3.82E-04 | 210 |
| NOL10 | 1 | 3.82E-04 | 211 |
| DDX19A | 18 | 3.82E-04 | 212 |
| PABPC4 | 45 | 3.82E-04 | 213 |
| XPO5 | 134 | 3.82E-04 | 214 |
| EIF5AL1 | 1 | 3.82E-04 | 215 |
| U2AF2 | 212 | 3.81E-04 | 216 |
| GNL2 | 26 | 3.81E-04 | 217 |
| XPOT | 51 | 3.81E-04 | 218 |
| FAM46A | 12 | 3.80E-04 | 219 |
| EIF2S3 | 525 | 3.80E-04 | 220 |
| DDX56 | 273 | 3.80E-04 | 221 |
| FBL | 934 | 3.80E-04 | 222 |
| AFF3 | 66 | 3.79E-04 | 223 |
| LSG1 | 15 | 3.79E-04 | 224 |
| NOP14 | 12 | 3.79E-04 | 225 |
| IFIT3 | 240 | 3.78E-04 | 226 |
| PPARGC1A | 565 | 3.78E-04 | 227 |
| NXF2 | 34 | 3.77E-04 | 228 |
| ZRANB2 | 41 | 3.77E-04 | 229 |
| RPS4Y1 | 45 | 3.76E-04 | 230 |
| PPARGC1B | 340 | 3.75E-04 | 231 |
| TRMU | 57 | 3.75E-04 | 232 |
| PSMA6 | 71 | 3.75E-04 | 233 |
| RPS27A | 239 | 3.74E-04 | 234 |
| INTS6 | 205 | 3.74E-04 | 235 |
| TAF9 | 63 | 3.72E-04 | 236 |
| SAP18 | 37 | 3.71E-04 | 237 |
| RRP8 | 74 | 3.71E-04 | 238 |
| GTPBP4 | 43 | 3.70E-04 | 239 |
| AEN | 36 | 3.69E-04 | 240 |
| SUB1 | 438 | 3.69E-04 | 241 |
| DRG1 | 56 | 3.67E-04 | 242 |
| RPLP1 | 583 | 3.64E-04 | 243 |
| RPL38 | 51 | 3.57E-04 | 244 |
| RPL23A | 54 | 3.53E-04 | 245 |
| KHDRBS1 | 397 | 1.81E-05 | 246 |
| MEX3D | 11 | 1.71E-05 | 247 |
| TIAL1 | 162 | 1.67E-05 | 248 |
| NOVA1 | 62 | 1.64E-05 | 249 |
| RBFOX2 | 472 | 1.64E-05 | 250 |
| ILF2 | 71 | 1.64E-05 | 251 |
| KHDRBS2 | 53 | 1.62E-05 | 252 |
| ILF3 | 207 | 1.62E-05 | 253 |
| PSPC1 | 78 | 1.62E-05 | 254 |
| ZCRB1 | 6 | 1.61E-05 | 255 |
| SERBP1 | 40 | 1.59E-05 | 256 |
| RBPMS | 22 | 1.58E-05 | 257 |
| RBM4 | 43 | 1.53E-05 | 258 |
| CELF2 | 100 | 1.53E-05 | 259 |
| FUBP1 | 125 | 1.52E-05 | 260 |
| RBBP6 | 54 | 1.50E-05 | 261 |
| QKI | 110 | 1.48E-05 | 262 |
| HDLBP | 151 | 1.47E-05 | 263 |
| PTBP2 | 149 | 1.47E-05 | 264 |
| RBM3 | 80 | 1.44E-05 | 265 |
| LRRFIP2 | 12 | 1.44E-05 | 266 |
| CLK2 | 38 | 1.43E-05 | 267 |
| TSNAX | 104 | 1.42E-05 | 268 |
| HABP4 | 20 | 1.42E-05 | 269 |
| CSDE1 | 62 | 1.42E-05 | 270 |
| RBM24 | 15 | 1.40E-05 | 271 |
| RBMS2 | 37 | 1.40E-05 | 272 |
| CELF1 | 249 | 1.39E-05 | 273 |
| RALYL | 6 | 1.38E-05 | 274 |
| R3HDM2 | 5 | 1.37E-05 | 275 |
| PUF60 | 49 | 1.37E-05 | 276 |
| CIRBP | 133 | 1.37E-05 | 277 |
| DDX17 | 327 | 1.37E-05 | 278 |
| TSN | 238 | 1.36E-05 | 279 |
| FUBP3 | 11 | 1.36E-05 | 280 |
| SYF2 | 65 | 1.36E-05 | 281 |
| YBX1 | 793 | 1.35E-05 | 282 |
| IGF2BP1 | 392 | 1.35E-05 | 283 |
| RRP36 | 32 | 1.35E-05 | 284 |
| ELAVL4 | 357 | 1.34E-05 | 285 |
| NCL | 1000 | 1.34E-05 | 286 |
| DDX5 | 576 | 1.33E-05 | 287 |
| PA2G4 | 126 | 1.33E-05 | 288 |
| SF1 | 755 | 1.32E-05 | 289 |
| ZNF326 | 4 | 1.32E-05 | 290 |
| XPO4 | 25 | 1.32E-05 | 291 |
| YBX3 | 41 | 1.32E-05 | 292 |
| JAKMIP1 | 15 | 1.32E-05 | 293 |
| PLRG1 | 32 | 1.31E-05 | 294 |
| RNPC3 | 1000 | 1.31E-05 | 295 |
| FMR1 | 1000 | 1.31E-05 | 296 |
| HNRNPLL | 4 | 1.30E-05 | 297 |
| DHX9 | 827 | 1.29E-05 | 298 |
| BCLAF1 | 119 | 1.29E-05 | 299 |
| SLC4A1AP | 12 | 1.28E-05 | 300 |
| PTRF | 164 | 1.28E-05 | 301 |
| FXR2 | 71 | 1.28E-05 | 302 |
| ELAVL1 | 1000 | 1.28E-05 | 303 |
| SFPQ | 913 | 1.28E-05 | 304 |
| SRSF6 | 271 | 1.28E-05 | 305 |
| ELAVL2 | 65 | 1.27E-05 | 306 |
| LARP1 | 29 | 1.26E-05 | 307 |
| TROVE2 | 50 | 1.25E-05 | 308 |
| SRSF11 | 17 | 1.24E-05 | 309 |
| TFIP11 | 146 | 1.23E-05 | 310 |
| CD2BP2 | 49 | 1.23E-05 | 311 |
| CLK3 | 21 | 1.22E-05 | 312 |
| C1D | 133 | 1.22E-05 | 313 |
| HELZ | 12 | 1.22E-05 | 314 |
| DRG2 | 24 | 1.21E-05 | 315 |
| NOVA2 | 20 | 1.21E-05 | 316 |
| XPO7 | 20 | 1.20E-05 | 317 |
| NR0B1 | 1000 | 1.18E-05 | 318 |
| EIF3I | 59 | 1.18E-05 | 319 |
| U2SURP | 8 | 1.17E-05 | 320 |
| RBM39 | 48 | 1.17E-05 | 321 |
| CSTF2 | 90 | 1.17E-05 | 322 |
| WBP4 | 14 | 1.17E-05 | 323 |
| ZFP36 | 572 | 1.16E-05 | 324 |
| CLK1 | 205 | 1.16E-05 | 325 |
| SAMD4A | 371 | 1.16E-05 | 326 |
| APOBEC2 | 67 | 1.16E-05 | 327 |
| RBM10 | 180 | 1.16E-05 | 328 |
| CELF6 | 3 | 1.16E-05 | 329 |
| RBPMS2 | 7 | 1.16E-05 | 330 |
| TCERG1 | 56 | 1.15E-05 | 331 |
| TIA1 | 777 | 1.15E-05 | 332 |
| U2AF1L4 | 9 | 1.15E-05 | 333 |
| DNTTIP2 | 27 | 1.15E-05 | 334 |
| MBNL2 | 45 | 1.15E-05 | 335 |
| USB1 | 79 | 1.15E-05 | 336 |
| CHERP | 8 | 1.15E-05 | 337 |
| IGF2BP3 | 133 | 1.15E-05 | 338 |
| FAM98B | 3 | 1.14E-05 | 339 |
| SIDT2 | 4 | 1.14E-05 | 340 |
| AKAP17A | 53 | 1.14E-05 | 341 |
| NFX1 | 21 | 1.14E-05 | 342 |
| MECP2 | 1000 | 1.13E-05 | 343 |
| NSRP1 | 4 | 1.13E-05 | 344 |
| RIOK3 | 26 | 1.13E-05 | 345 |
| SRSF8 | 18 | 1.13E-05 | 346 |
| SART3 | 150 | 1.13E-05 | 347 |
| ZC3H12D | 263 | 1.13E-05 | 348 |
| SNRPA | 385 | 1.13E-05 | 349 |
| RC3H2 | 7 | 1.12E-05 | 350 |
| CPEB4 | 36 | 1.12E-05 | 351 |
| ZC3H13 | 7 | 1.12E-05 | 352 |
| ANKRD17 | 16 | 1.12E-05 | 353 |
| RIOK1 | 9 | 1.12E-05 | 354 |
| SNW1 | 71 | 1.12E-05 | 355 |
| LSM4 | 238 | 1.11E-05 | 356 |
| UBAP2L | 15 | 1.11E-05 | 357 |
| AZGP1 | 222 | 1.11E-05 | 358 |
| ESRP2 | 19 | 1.11E-05 | 359 |
| SRSF12 | 2 | 1.11E-05 | 360 |
| RBM8A | 186 | 1.11E-05 | 361 |
| G3BP2 | 24 | 1.11E-05 | 362 |
| RBM20 | 23 | 1.11E-05 | 363 |
| CARHSP1 | 12 | 1.10E-05 | 364 |
| MBNL3 | 23 | 1.10E-05 | 365 |
| CLK4 | 22 | 1.10E-05 | 366 |
| RPSA | 377 | 1.08E-05 | 367 |
| XAB2 | 39 | 1.08E-05 | 368 |
| MBNL1 | 226 | 1.08E-05 | 369 |
| ZCCHC17 | 7 | 1.08E-05 | 370 |
| NXT2 | 15 | 1.08E-05 | 371 |
| CASC3 | 90 | 1.07E-05 | 372 |
| SECISBP2 | 134 | 1.07E-05 | 373 |
| REXO1 | 3 | 1.07E-05 | 374 |
| SREK1 | 9 | 1.07E-05 | 375 |
| RARS | 695 | 1.06E-05 | 376 |
| YTHDC2 | 6 | 1.06E-05 | 377 |
| RBM38 | 31 | 1.06E-05 | 378 |
| SRRM4 | 12 | 1.05E-05 | 379 |
| PNN | 653 | 1.05E-05 | 380 |
| FXR1 | 118 | 1.05E-05 | 381 |
| CELF4 | 37 | 1.05E-05 | 382 |
| HINT3 | 6 | 1.05E-05 | 383 |
| C1QBP | 1000 | 1.05E-05 | 384 |
| USP10 | 48 | 1.05E-05 | 385 |
| NXT1 | 1000 | 1.05E-05 | 386 |
| ARL6IP4 | 38 | 1.04E-05 | 387 |
| SART1 | 69 | 1.04E-05 | 388 |
| GLTSCR2 | 264 | 1.04E-05 | 389 |
| CACTIN | 13 | 1.04E-05 | 390 |
| RTCA | 294 | 1.03E-05 | 391 |
| EIF4G2 | 677 | 1.03E-05 | 392 |
| RBM33 | 4 | 1.03E-05 | 393 |
| SRP68 | 29 | 1.03E-05 | 394 |
| TCOF1 | 173 | 1.02E-05 | 395 |
| USP39 | 71 | 1.02E-05 | 396 |
| RNGTT | 258 | 1.02E-05 | 397 |
| HEXIM1 | 168 | 1.02E-05 | 398 |
| IFIT1 | 687 | 1.02E-05 | 399 |
| FIP1L1 | 419 | 1.02E-05 | 400 |
| FAM103A1 | 8 | 1.02E-05 | 401 |
| SRP72 | 37 | 1.02E-05 | 402 |
| ADARB2 | 66 | 1.02E-05 | 403 |
| SRFBP1 | 35 | 1.02E-05 | 404 |
| PARK7 | 1000 | 1.01E-05 | 405 |
| ADAR | 672 | 1.01E-05 | 406 |
| DYNLL1 | 181 | 1.01E-05 | 407 |
| FASTK | 36 | 1.01E-05 | 408 |
| NOM1 | 24 | 1.01E-05 | 409 |
| GCFC2 | 87 | 1.00E-05 | 410 |
| ALKBH1 | 1000 | 1.00E-05 | 411 |
| ZCCHC8 | 4 | 1.00E-05 | 412 |
| IFIT5 | 62 | 9.99E-06 | 413 |
| CCDC59 | 5 | 9.99E-06 | 414 |
| CEBPZ | 392 | 9.99E-06 | 415 |
| STAU2 | 28 | 9.98E-06 | 416 |
| PPIE | 20 | 9.97E-06 | 417 |
| EXOSC2 | 784 | 9.96E-06 | 418 |
| DEK | 229 | 9.96E-06 | 419 |
| CALR | 1000 | 9.93E-06 | 420 |
| SRRM1 | 38 | 9.92E-06 | 421 |
| ZFP36L1 | 173 | 9.91E-06 | 422 |
| MSI1 | 397 | 9.91E-06 | 423 |
| DNAAF2 | 34 | 9.84E-06 | 424 |
| RUVBL1 | 189 | 9.83E-06 | 425 |
| PTGES3 | 1000 | 9.81E-06 | 426 |
| DDX6 | 247 | 9.71E-06 | 427 |
| SNRPE | 1000 | 9.71E-06 | 428 |
| PRPF40A | 143 | 9.71E-06 | 429 |
| PHF5A | 130 | 9.71E-06 | 430 |
| SAMD4B | 16 | 9.71E-06 | 431 |
| SRRT | 81 | 9.70E-06 | 432 |
| METTL5 | 2 | 9.69E-06 | 433 |
| ENOX1 | 43 | 9.68E-06 | 434 |
| SCAF11 | 30 | 9.68E-06 | 435 |
| SRP9 | 52 | 9.63E-06 | 436 |
| U2AF1 | 134 | 9.62E-06 | 437 |
| NUFIP2 | 620 | 9.57E-06 | 438 |
| RBM46 | 6 | 9.57E-06 | 439 |
| SNRNP27 | 16 | 9.56E-06 | 440 |
| PCBP4 | 146 | 9.51E-06 | 441 |
| DDX50 | 11 | 9.49E-06 | 442 |
| MAGOH | 57 | 9.48E-06 | 443 |
| TGS1 | 91 | 9.46E-06 | 444 |
| YBX2 | 76 | 9.44E-06 | 445 |
| LUZP4 | 41 | 9.44E-06 | 446 |
| C17orf85 | 37 | 9.42E-06 | 447 |
| TIPARP | 348 | 9.39E-06 | 448 |
| MAZ | 228 | 9.39E-06 | 449 |
| YTHDF2 | 12 | 9.38E-06 | 450 |
| RQCD1 | 23 | 9.37E-06 | 451 |
| ZC3H15 | 6 | 9.36E-06 | 452 |
| RBM15 | 240 | 9.36E-06 | 453 |
| CCRN4L | 114 | 9.35E-06 | 454 |
| RBM7 | 14 | 9.35E-06 | 455 |
| MAGOHB | 54 | 9.31E-06 | 456 |
| CNP | 1000 | 9.31E-06 | 457 |
| LUC7L | 15 | 9.31E-06 | 458 |
| ELAVL3 | 477 | 9.30E-06 | 459 |
| EXOSC9 | 485 | 9.28E-06 | 460 |
| DDX43 | 71 | 9.28E-06 | 461 |
| STRBP | 148 | 9.28E-06 | 462 |
| AIMP1 | 410 | 9.27E-06 | 463 |
| SRSF5 | 1000 | 9.27E-06 | 464 |
| LARP6 | 352 | 9.27E-06 | 465 |
| PSIP1 | 1000 | 9.26E-06 | 466 |
| REXO4 | 39 | 9.26E-06 | 467 |
| MVP | 336 | 9.26E-06 | 468 |
| HELZ2 | 22 | 9.22E-06 | 469 |
| RPS19BP1 | 23 | 9.22E-06 | 470 |
| SPATS2L | 17 | 9.20E-06 | 471 |
| KIAA0020 | 54 | 9.18E-06 | 472 |
| RBM34 | 4 | 9.17E-06 | 473 |
| DDX1 | 91 | 9.16E-06 | 474 |
| CPEB3 | 36 | 9.15E-06 | 475 |
| TEP1 | 314 | 9.15E-06 | 476 |
| WIBG | 69 | 9.14E-06 | 477 |
| RUVBL2 | 166 | 9.13E-06 | 478 |
| SRRM3 | 2 | 9.13E-06 | 479 |
| DDX3X | 277 | 9.13E-06 | 480 |
| CRYZ | 1000 | 9.13E-06 | 481 |
| N4BP1 | 8 | 9.12E-06 | 482 |
| RP9 | 341 | 9.12E-06 | 483 |
| PRIM1 | 229 | 9.10E-06 | 484 |
| GTF3A | 1000 | 9.10E-06 | 485 |
| MRPS7 | 469 | 9.10E-06 | 486 |
| CD3EAP | 68 | 9.10E-06 | 487 |
| KRR1 | 73 | 9.09E-06 | 488 |
| NPM1 | 1000 | 9.09E-06 | 489 |
| PTRH1 | 308 | 9.09E-06 | 490 |
| RNMT | 262 | 9.07E-06 | 491 |
| SRPK2 | 33 | 9.07E-06 | 492 |
| ACIN1 | 1000 | 9.06E-06 | 493 |
| EIF3M | 1000 | 9.05E-06 | 494 |
| DUSP11 | 37 | 9.04E-06 | 495 |
| NOL7 | 8 | 9.03E-06 | 496 |
| UBA1 | 248 | 9.03E-06 | 497 |
| INTS7 | 12 | 9.02E-06 | 498 |
| MEX3B | 3 | 9.02E-06 | 499 |
| SNIP1 | 29 | 9.01E-06 | 500 |
| SRP19 | 84 | 8.99E-06 | 501 |
| RBFOX3 | 5 | 8.96E-06 | 502 |
| SF3B3 | 19 | 8.95E-06 | 503 |
| IFIT2 | 133 | 8.94E-06 | 504 |
| TRA2A | 11 | 8.94E-06 | 505 |
| INTS8 | 15 | 8.92E-06 | 506 |
| NOL3 | 1000 | 8.91E-06 | 507 |
| EXOSC8 | 588 | 8.88E-06 | 508 |
| DDX31 | 2 | 8.87E-06 | 509 |
| LUC7L2 | 5 | 8.87E-06 | 510 |
| SMAD9 | 139 | 8.87E-06 | 511 |
| UHMK1 | 522 | 8.85E-06 | 512 |
| EEF1E1 | 147 | 8.85E-06 | 513 |
| CCDC86 | 9 | 8.84E-06 | 514 |
| HRSP12 | 104 | 8.84E-06 | 515 |
| SF3A1 | 43 | 8.82E-06 | 516 |
| NUDT16 | 36 | 8.82E-06 | 517 |
| PELO | 25 | 8.81E-06 | 518 |
| TRIM25 | 214 | 8.81E-06 | 519 |
| IPO9 | 21 | 8.81E-06 | 520 |
| GSPT1 | 103 | 8.80E-06 | 521 |
| RPS15A | 49 | 8.80E-06 | 522 |
| ANKHD1 | 10 | 8.80E-06 | 523 |
| MEX3A | 2 | 8.78E-06 | 524 |
| AQR | 39 | 8.77E-06 | 525 |
| APEX1 | 1000 | 8.76E-06 | 526 |
| ACO1 | 533 | 8.76E-06 | 527 |
| EBNA1BP2 | 508 | 8.76E-06 | 528 |
| RRP1B | 19 | 8.74E-06 | 529 |
| TOP3B | 13 | 8.73E-06 | 530 |
| LRRC47 | 2 | 8.73E-06 | 531 |
| DGCR14 | 178 | 8.73E-06 | 532 |
| GEMIN4 | 94 | 8.72E-06 | 533 |
| EED | 356 | 8.72E-06 | 534 |
| AKAP1 | 468 | 8.72E-06 | 535 |
| TBRG4 | 27 | 8.71E-06 | 536 |
| ERI1 | 178 | 8.70E-06 | 537 |
| A1CF | 75 | 8.70E-06 | 538 |
| CCAR1 | 58 | 8.69E-06 | 539 |
| ENOX2 | 67 | 8.69E-06 | 540 |
| GSPT2 | 35 | 8.68E-06 | 541 |
| MCTS1 | 113 | 8.68E-06 | 542 |
| GTPBP2 | 8 | 8.67E-06 | 543 |
| NAT10 | 62 | 8.66E-06 | 544 |
| NOC3L | 16 | 8.66E-06 | 545 |
| ZCCHC6 | 283 | 8.63E-06 | 546 |
| PRPF38A | 8 | 8.62E-06 | 547 |
| REPIN1 | 66 | 8.61E-06 | 548 |
| SUGP1 | 99 | 8.61E-06 | 549 |
| PABPC3 | 4 | 8.61E-06 | 550 |
| RBM25 | 13 | 8.61E-06 | 551 |
| R3HCC1L | 73 | 8.59E-06 | 552 |
| EIF2AK3 | 342 | 8.57E-06 | 553 |
| EEF1G | 24 | 8.56E-06 | 554 |
| ZC3H3 | 3 | 8.55E-06 | 555 |
| EMG1 | 151 | 8.54E-06 | 556 |
| MSI2 | 76 | 8.53E-06 | 557 |
| SRPR | 82 | 8.50E-06 | 558 |
| NAF1 | 28 | 8.50E-06 | 559 |
| CPSF4 | 473 | 8.47E-06 | 560 |
| INTS9 | 8 | 8.46E-06 | 561 |
| EXOSC1 | 260 | 8.46E-06 | 562 |
| DDX25 | 78 | 8.45E-06 | 563 |
| SF3B2 | 35 | 8.44E-06 | 564 |
| SMAD6 | 351 | 8.43E-06 | 565 |
| MRPL49 | 126 | 8.40E-06 | 566 |
| RC3H1 | 54 | 8.40E-06 | 567 |
| EDC4 | 108 | 8.38E-06 | 568 |
| PPIL3 | 14 | 8.37E-06 | 569 |
| NHP2L1 | 218 | 8.34E-06 | 570 |
| CPEB2 | 14 | 8.34E-06 | 571 |
| IARS | 1000 | 8.33E-06 | 572 |
| SYMPK | 364 | 8.32E-06 | 573 |
| ABCF1 | 34 | 8.32E-06 | 574 |
| TNPO1 | 943 | 8.32E-06 | 575 |
| OAS3 | 183 | 8.31E-06 | 576 |
| FAU | 230 | 8.30E-06 | 577 |
| SCAF4 | 6 | 8.28E-06 | 578 |
| CLP1 | 71 | 8.27E-06 | 579 |
| PARP12 | 10 | 8.27E-06 | 580 |
| KHDRBS3 | 231 | 8.24E-06 | 581 |
| EEF1A2 | 1000 | 8.21E-06 | 582 |
| SND1 | 1000 | 8.21E-06 | 583 |
| DHX32 | 12 | 8.21E-06 | 584 |
| SUZ12 | 293 | 8.20E-06 | 585 |
| DHX40 | 8 | 8.20E-06 | 586 |
| FBXO17 | 5 | 8.20E-06 | 587 |
| GEMIN6 | 59 | 8.19E-06 | 588 |
| TDRD7 | 38 | 8.19E-06 | 589 |
| GTPBP3 | 47 | 8.19E-06 | 590 |
| SF3B1 | 308 | 8.19E-06 | 591 |
| DDX41 | 1000 | 8.18E-06 | 592 |
| EIF2B5 | 272 | 8.18E-06 | 593 |
| MRPL17 | 38 | 8.18E-06 | 594 |
| CDK9 | 1000 | 8.17E-06 | 595 |
| DHX36 | 38 | 8.16E-06 | 596 |
| TRMT1L | 1 | 8.13E-06 | 597 |
| PEG10 | 285 | 8.13E-06 | 598 |
| RRS1 | 720 | 8.10E-06 | 599 |
| NUDT21 | 12 | 8.08E-06 | 600 |
| SRP54 | 196 | 8.05E-06 | 601 |
| SMAD1 | 1000 | 8.04E-06 | 602 |
| RPP14 | 130 | 8.03E-06 | 603 |
| SIDT1 | 102 | 8.02E-06 | 604 |
| SNRNP35 | 225 | 8.02E-06 | 605 |
| TERT | 1000 | 8.02E-06 | 606 |
| GTPBP1 | 475 | 8.01E-06 | 607 |
| EXOSC10 | 1000 | 8.00E-06 | 608 |
| TRIM21 | 1000 | 7.99E-06 | 609 |
| SLTM | 376 | 7.98E-06 | 610 |
| SMG1 | 212 | 7.97E-06 | 611 |
| RBMXL1 | 14 | 7.96E-06 | 612 |
| FAM120C | 28 | 7.96E-06 | 613 |
| MYEF2 | 159 | 7.95E-06 | 614 |
| POLR2D | 18 | 7.94E-06 | 615 |
| IPO4 | 10 | 7.92E-06 | 616 |
| BMS1 | 443 | 7.92E-06 | 617 |
| SMAD5 | 395 | 7.89E-06 | 618 |
| DDX21 | 429 | 7.86E-06 | 619 |
| YRDC | 43 | 7.85E-06 | 620 |
| GAPDH | 1000 | 7.82E-06 | 621 |
| UNK | 67 | 7.76E-06 | 622 |
| AUH | 82 | 7.75E-06 | 623 |
| ERAL1 | 57 | 7.75E-06 | 624 |
| LARP4B | 6 | 7.75E-06 | 625 |
| RRP7A | 1 | 7.74E-06 | 626 |
| RBM47 | 6 | 7.74E-06 | 627 |
| APOBEC1 | 266 | 7.74E-06 | 628 |
| DNMT3B | 1000 | 7.73E-06 | 629 |
| NYNRIN | 3 | 7.72E-06 | 630 |
| ZC3H12B | 4 | 7.71E-06 | 631 |
| YTHDF1 | 4 | 7.71E-06 | 632 |
| MEX3C | 10 | 7.71E-06 | 633 |
| ASCC1 | 999 | 7.67E-06 | 634 |
| NUDT16L1 | 7 | 7.67E-06 | 635 |
| LUC7L3 | 296 | 7.66E-06 | 636 |
| SLIRP | 29 | 7.66E-06 | 637 |
| PPP1R10 | 298 | 7.66E-06 | 638 |
| DHX37 | 3 | 7.65E-06 | 639 |
| SARS | 1000 | 7.64E-06 | 640 |
| RPS27 | 338 | 7.64E-06 | 641 |
| SMAD2 | 1000 | 7.64E-06 | 642 |
| NGDN | 174 | 7.63E-06 | 643 |
| RNASE2 | 503 | 7.63E-06 | 644 |
| DDX42 | 14 | 7.61E-06 | 645 |
| MRPS31 | 4 | 7.60E-06 | 646 |
| RPLP2 | 1000 | 7.59E-06 | 647 |
| SF3B14 | 3 | 7.57E-06 | 648 |
| LARP7 | 29 | 7.55E-06 | 649 |
| DHX16 | 110 | 7.55E-06 | 650 |
| PURA | 290 | 7.54E-06 | 651 |
| CNBP | 1000 | 7.54E-06 | 652 |
| LARP4 | 4 | 7.53E-06 | 653 |
| QRSL1 | 1000 | 7.52E-06 | 654 |
| MKRN2 | 8 | 7.51E-06 | 655 |
| SRP14 | 40 | 7.50E-06 | 656 |
| DDX54 | 6 | 7.50E-06 | 657 |
| TDRD9 | 24 | 7.50E-06 | 658 |
| ERI3 | 4 | 7.49E-06 | 659 |
| IMP3 | 183 | 7.49E-06 | 660 |
| ZNF385A | 8 | 7.48E-06 | 661 |
| SMAD4 | 1000 | 7.48E-06 | 662 |
| EIF2AK1 | 336 | 7.45E-06 | 663 |
| EXOG | 510 | 7.41E-06 | 664 |
| AFF1 | 1000 | 7.39E-06 | 665 |
| ERN2 | 26 | 7.39E-06 | 666 |
| HBS1L | 288 | 7.38E-06 | 667 |
| DDX18 | 11 | 7.38E-06 | 668 |
| SBDS | 209 | 7.38E-06 | 669 |
| ALKBH5 | 19 | 7.37E-06 | 670 |
| SMAD7 | 1000 | 7.37E-06 | 671 |
| DZIP1 | 29 | 7.37E-06 | 672 |
| DDX3Y | 128 | 7.35E-06 | 673 |
| PUM1 | 56 | 7.35E-06 | 674 |
| EIF4A3 | 86 | 7.34E-06 | 675 |
| OAS1 | 212 | 7.34E-06 | 676 |
| DZIP3 | 6 | 7.32E-06 | 677 |
| PTRH2 | 31 | 7.31E-06 | 678 |
| TSEN15 | 7 | 7.31E-06 | 679 |
| ISY1 | 6 | 7.30E-06 | 680 |
| STAU1 | 70 | 7.29E-06 | 681 |
| DHX8 | 63 | 7.26E-06 | 682 |
| DHX15 | 71 | 7.26E-06 | 683 |
| SLBP | 210 | 7.26E-06 | 684 |
| RANBP6 | 4 | 7.24E-06 | 685 |
| RANBP17 | 16 | 7.23E-06 | 686 |
| PAPOLB | 33 | 7.23E-06 | 687 |
| EIF3A | 1000 | 7.21E-06 | 688 |
| OAS2 | 67 | 7.20E-06 | 689 |
| PPIL4 | 262 | 7.20E-06 | 690 |
| CANX | 1000 | 7.20E-06 | 691 |
| RBM22 | 19 | 7.20E-06 | 692 |
| ZMAT3 | 44 | 7.18E-06 | 693 |
| INTS4 | 10 | 7.17E-06 | 694 |
| RRBP1 | 1000 | 7.15E-06 | 695 |
| TST | 1000 | 7.15E-06 | 696 |
| FAM120A | 112 | 7.14E-06 | 697 |
| IFIH1 | 899 | 7.14E-06 | 698 |
| TOP1 | 1000 | 7.13E-06 | 699 |
| CDK5RAP1 | 9 | 7.12E-06 | 700 |
| GEMIN2 | 105 | 7.12E-06 | 701 |
| UTP3 | 33 | 7.10E-06 | 702 |
| TLR3 | 1000 | 7.07E-06 | 703 |
| RBM26 | 4 | 7.07E-06 | 704 |
| NPM3 | 21 | 7.05E-06 | 705 |
| PINX1 | 102 | 7.04E-06 | 706 |
| RPLP0 | 1000 | 7.04E-06 | 707 |
| XRCC6 | 1000 | 7.01E-06 | 708 |
| LONP1 | 777 | 7.01E-06 | 709 |
| PARN | 218 | 6.99E-06 | 710 |
| HTATSF1 | 24 | 6.98E-06 | 711 |
| GEMIN7 | 23 | 6.98E-06 | 712 |
| NOP16 | 10 | 6.98E-06 | 713 |
| METTL3 | 40 | 6.97E-06 | 714 |
| MKRN1 | 24 | 6.96E-06 | 715 |
| KHDC1 | 3 | 6.96E-06 | 716 |
| PIN4 | 67 | 6.96E-06 | 717 |
| SMAD3 | 1000 | 6.96E-06 | 718 |
| RBMY1A1 | 126 | 6.95E-06 | 719 |
| TNPO3 | 67 | 6.94E-06 | 720 |
| ZNF622 | 4 | 6.92E-06 | 721 |
| GNL3 | 224 | 6.90E-06 | 722 |
| TLR8 | 607 | 6.89E-06 | 723 |
| EIF1AD | 3 | 6.89E-06 | 724 |
| WDR36 | 68 | 6.89E-06 | 725 |
| PRPF19 | 133 | 6.87E-06 | 726 |
| DDX20 | 83 | 6.86E-06 | 727 |
| SSB | 1000 | 6.85E-06 | 728 |
| EIF2AK2 | 1000 | 6.85E-06 | 729 |
| DHX58 | 245 | 6.84E-06 | 730 |
| PSMA1 | 624 | 6.84E-06 | 731 |
| CNOT3 | 33 | 6.83E-06 | 732 |
| DNAJC17 | 6 | 6.83E-06 | 733 |
| EXOSC7 | 76 | 6.82E-06 | 734 |
| APOBEC4 | 8 | 6.81E-06 | 735 |
| ZC3H12A | 93 | 6.79E-06 | 736 |
| BZW1 | 62 | 6.78E-06 | 737 |
| SLU7 | 11 | 6.78E-06 | 738 |
| MTFMT | 162 | 6.77E-06 | 739 |
| CWC27 | 2 | 6.75E-06 | 740 |
| EIF3D | 19 | 6.75E-06 | 741 |
| GPATCH4 | 2 | 6.75E-06 | 742 |
| SMG8 | 9 | 6.72E-06 | 743 |
| FARSA | 465 | 6.72E-06 | 744 |
| PUM2 | 68 | 6.72E-06 | 745 |
| RNASE3 | 1000 | 6.71E-06 | 746 |
| SAMHD1 | 367 | 6.70E-06 | 747 |
| UTP20 | 30 | 6.69E-06 | 748 |
| PDE12 | 39 | 6.69E-06 | 749 |
| DQX1 | 2 | 6.69E-06 | 750 |
| FCF1 | 60 | 6.67E-06 | 751 |
| NOLC1 | 83 | 6.67E-06 | 752 |
| WDR61 | 39 | 6.65E-06 | 753 |
| EIF3K | 8 | 6.64E-06 | 754 |
| PAPOLA | 444 | 6.64E-06 | 755 |
| CALR3 | 36 | 6.63E-06 | 756 |
| CCNT2 | 41 | 6.63E-06 | 757 |
| SFSWAP | 4 | 6.60E-06 | 758 |
| EIF1B | 3 | 6.60E-06 | 759 |
| MOV10 | 46 | 6.59E-06 | 760 |
| ADAD2 | 1 | 6.59E-06 | 761 |
| RSRC1 | 6 | 6.58E-06 | 762 |
| AARSD1 | 2 | 6.58E-06 | 763 |
| CNOT7 | 74 | 6.57E-06 | 764 |
| SNRPC | 65 | 6.57E-06 | 765 |
| DHX38 | 72 | 6.57E-06 | 766 |
| SNRPF | 120 | 6.56E-06 | 767 |
| PPAN | 355 | 6.56E-06 | 768 |
| ABT1 | 9 | 6.55E-06 | 769 |
| IREB2 | 393 | 6.54E-06 | 770 |
| PABPC1 | 617 | 6.50E-06 | 771 |
| DNAJC21 | 77 | 6.50E-06 | 772 |
| RPL27A | 43 | 6.50E-06 | 773 |
| CPSF6 | 40 | 6.47E-06 | 774 |
| CSTF3 | 24 | 6.46E-06 | 775 |
| DDX58 | 1000 | 6.45E-06 | 776 |
| PATL1 | 15 | 6.44E-06 | 777 |
| LARS | 877 | 6.43E-06 | 778 |
| TXNL4B | 322 | 6.43E-06 | 779 |
| URB2 | 5 | 6.40E-06 | 780 |
| TBL3 | 20 | 6.38E-06 | 781 |
| PARP4 | 273 | 6.38E-06 | 782 |
| NANOS3 | 172 | 6.37E-06 | 783 |
| EFTUD1 | 19 | 6.37E-06 | 784 |
| UTP15 | 4 | 6.36E-06 | 785 |
| TARBP2 | 623 | 6.36E-06 | 786 |
| SEPSECS | 327 | 6.36E-06 | 787 |
| RBM12 | 3 | 6.35E-06 | 788 |
| OASL | 55 | 6.35E-06 | 789 |
| LRPPRC | 180 | 6.34E-06 | 790 |
| DCPS | 199 | 6.33E-06 | 791 |
| PRPF6 | 111 | 6.31E-06 | 792 |
| CRNKL1 | 15 | 6.30E-06 | 793 |
| EXOSC5 | 32 | 6.30E-06 | 794 |
| ZFR2 | 3 | 6.29E-06 | 795 |
| BAZ2B | 8 | 6.29E-06 | 796 |
| EIF2AK4 | 425 | 6.29E-06 | 797 |
| PURB | 109 | 6.29E-06 | 798 |
| DDX28 | 5 | 6.27E-06 | 799 |
| SNRNP40 | 9 | 6.26E-06 | 800 |
| AIMP2 | 1000 | 6.24E-06 | 801 |
| CTU2 | 47 | 6.24E-06 | 802 |
| APOBEC3H | 68 | 6.22E-06 | 803 |
| CSDC2 | 3 | 6.20E-06 | 804 |
| HEXIM2 | 12 | 6.19E-06 | 805 |
| APOBEC3F | 238 | 6.13E-06 | 806 |
| PABPN1L | 4 | 6.11E-06 | 807 |
| CDC5L | 270 | 6.11E-06 | 808 |
| FTSJ1 | 420 | 6.10E-06 | 809 |
| STRAP | 48 | 6.09E-06 | 810 |
| SECISBP2L | 40 | 6.08E-06 | 811 |
| BZW2 | 4 | 6.06E-06 | 812 |
| ABCE1 | 173 | 6.06E-06 | 813 |
| NCBP2 | 82 | 6.05E-06 | 814 |
| ERN1 | 1000 | 6.03E-06 | 815 |
| LIN28B | 171 | 6.02E-06 | 816 |
| ELAC1 | 181 | 6.02E-06 | 817 |
| PRPF31 | 114 | 6.01E-06 | 818 |
| TRIT1 | 610 | 6.00E-06 | 819 |
| DDX23 | 24 | 5.98E-06 | 820 |
| MKRN3 | 95 | 5.97E-06 | 821 |
| EXO1 | 688 | 5.94E-06 | 822 |
| CWC15 | 482 | 5.93E-06 | 823 |
| TNRC6B | 25 | 5.93E-06 | 824 |
| OBFC1 | 124 | 5.92E-06 | 825 |
| RPL34 | 161 | 5.92E-06 | 826 |
| CPSF1 | 147 | 5.91E-06 | 827 |
| EFTUD2 | 46 | 5.89E-06 | 828 |
| GPATCH8 | 3 | 5.89E-06 | 829 |
| EZH2 | 1000 | 5.88E-06 | 830 |
| PRPF4 | 60 | 5.87E-06 | 831 |
| CLASRP | 2 | 5.87E-06 | 832 |
| PRPF3 | 347 | 5.84E-06 | 833 |
| RRP1 | 50 | 5.83E-06 | 834 |
| ZCCHC5 | 2 | 5.80E-06 | 835 |
| MRPL52 | 1 | 5.80E-06 | 836 |
| RNASET2 | 88 | 5.80E-06 | 837 |
| NCBP1 | 117 | 5.79E-06 | 838 |
| SNRPB2 | 32 | 5.78E-06 | 839 |
| BAZ2A | 41 | 5.77E-06 | 840 |
| AGO1 | 110 | 5.76E-06 | 841 |
| CPSF7 | 4 | 5.76E-06 | 842 |
| LSM3 | 12 | 5.76E-06 | 843 |
| TLR7 | 1000 | 5.76E-06 | 844 |
| UTP11L | 3 | 5.74E-06 | 845 |
| PWP2 | 23 | 5.72E-06 | 846 |
| TSEN34 | 10 | 5.72E-06 | 847 |
| IPO11 | 17 | 5.68E-06 | 848 |
| SNRPA1 | 17 | 5.67E-06 | 849 |
| MSL3 | 44 | 5.67E-06 | 850 |
| C11orf68 | 3 | 5.67E-06 | 851 |
| ZFC3H1 | 3 | 5.65E-06 | 852 |
| RBM28 | 8 | 5.64E-06 | 853 |
| PPWD1 | 4 | 5.63E-06 | 854 |
| MRPL21 | 4 | 5.63E-06 | 855 |
| EIF4H | 44 | 5.62E-06 | 856 |
| DICER1 | 454 | 5.61E-06 | 857 |
| PARP1 | 1000 | 5.60E-06 | 858 |
| TDRD6 | 14 | 5.60E-06 | 859 |
| DZIP1L | 2 | 5.60E-06 | 860 |
| PDCD4 | 474 | 5.59E-06 | 861 |
| POLR2J2 | 3 | 5.57E-06 | 862 |
| RBMX2 | 4 | 5.56E-06 | 863 |
| ZCCHC13 | 3 | 5.56E-06 | 864 |
| CPSF2 | 19 | 5.55E-06 | 865 |
| PRKDC | 1000 | 5.54E-06 | 866 |
| DDX10 | 21 | 5.52E-06 | 867 |
| SNRPD2 | 21 | 5.52E-06 | 868 |
| MRPL19 | 27 | 5.51E-06 | 869 |
| CDC40 | 38 | 5.51E-06 | 870 |
| EXOSC4 | 113 | 5.51E-06 | 871 |
| UTP6 | 19 | 5.50E-06 | 872 |
| KAT8 | 1000 | 5.48E-06 | 873 |
| HNRNPUL2 | 3 | 5.48E-06 | 874 |
| WRAP53 | 49 | 5.47E-06 | 875 |
| PIWIL2 | 244 | 5.46E-06 | 876 |
| NPM2 | 32 | 5.46E-06 | 877 |
| CPEB1 | 274 | 5.46E-06 | 878 |
| TRIM71 | 67 | 5.45E-06 | 879 |
| DNMT1 | 1000 | 5.45E-06 | 880 |
| EIF5A2 | 59 | 5.44E-06 | 881 |
| AGO2 | 225 | 5.43E-06 | 882 |
| NUFIP1 | 19 | 5.43E-06 | 883 |
| SCAF8 | 4 | 5.42E-06 | 884 |
| RNASE10 | 7 | 5.41E-06 | 885 |
| CWC22 | 514 | 5.40E-06 | 886 |
| GPKOW | 73 | 5.39E-06 | 887 |
| DIS3L2 | 25 | 5.37E-06 | 888 |
| DHX33 | 8 | 5.35E-06 | 889 |
| PES1 | 1000 | 5.33E-06 | 890 |
| INTS5 | 16 | 5.33E-06 | 891 |
| EIF4G1 | 999 | 5.32E-06 | 892 |
| DDX49 | 1 | 5.30E-06 | 893 |
| MOV10L1 | 14 | 5.29E-06 | 894 |
| PRPF4B | 47 | 5.28E-06 | 895 |
| PDCD7 | 28 | 5.27E-06 | 896 |
| DND1 | 58 | 5.26E-06 | 897 |
| URB1 | 6 | 5.24E-06 | 898 |
| TOE1 | 14 | 5.23E-06 | 899 |
| ENDOV | 382 | 5.23E-06 | 900 |
| FAM120B | 10 | 5.22E-06 | 901 |
| SNRPB | 87 | 5.22E-06 | 902 |
| CNOT1 | 120 | 5.22E-06 | 903 |
| ZNHIT6 | 3 | 5.20E-06 | 904 |
| SMNDC1 | 22 | 5.19E-06 | 905 |
| EEF2K | 186 | 5.17E-06 | 906 |
| NSUN7 | 6 | 5.17E-06 | 907 |
| ZC3H11A | 6 | 5.17E-06 | 908 |
| TRMT2A | 7 | 5.16E-06 | 909 |
| MRPS6 | 6 | 5.16E-06 | 910 |
| ZC3H6 | 1 | 5.16E-06 | 911 |
| GFM2 | 34 | 5.15E-06 | 912 |
| EEF2 | 1000 | 5.15E-06 | 913 |
| ICT1 | 339 | 5.15E-06 | 914 |
| NOC2L | 1000 | 5.14E-06 | 915 |
| LSM5 | 10 | 5.13E-06 | 916 |
| RBM12B | 2 | 5.11E-06 | 917 |
| RPF1 | 14 | 5.10E-06 | 918 |
| AFF4 | 46 | 5.09E-06 | 919 |
| DAP3 | 67 | 5.08E-06 | 920 |
| MAK16 | 13 | 5.08E-06 | 921 |
| EIF5A | 467 | 5.07E-06 | 922 |
| PRPF18 | 34 | 5.05E-06 | 923 |
| LIN28A | 546 | 5.05E-06 | 924 |
| CELF3 | 353 | 5.04E-06 | 925 |
| EIF4A1 | 575 | 5.02E-06 | 926 |
| ZCCHC11 | 35 | 5.01E-06 | 927 |
| CCNT1 | 365 | 5.00E-06 | 928 |
| DAZ1 | 313 | 4.98E-06 | 929 |
| RNASE9 | 9 | 4.97E-06 | 930 |
| REXO2 | 8 | 4.96E-06 | 931 |
| DUS3L | 1 | 4.96E-06 | 932 |
| TXNL4A | 28 | 4.96E-06 | 933 |
| RPL18A | 31 | 4.96E-06 | 934 |
| ADAD1 | 18 | 4.95E-06 | 935 |
| CNOT8 | 765 | 4.93E-06 | 936 |
| XRN1 | 177 | 4.93E-06 | 937 |
| RPL3 | 1000 | 4.92E-06 | 938 |
| HNRNPCL1 | 2 | 4.92E-06 | 939 |
| NIP7 | 17 | 4.90E-06 | 940 |
| CSTF1 | 17 | 4.89E-06 | 941 |
| DDX47 | 9 | 4.89E-06 | 942 |
| MAEL | 41 | 4.88E-06 | 943 |
| RNASE7 | 143 | 4.88E-06 | 944 |
| GUF1 | 58 | 4.87E-06 | 945 |
| ZC3HAV1 | 48 | 4.87E-06 | 946 |
| POP1 | 68 | 4.86E-06 | 947 |
| SPATS2 | 1 | 4.86E-06 | 948 |
| CWC25 | 11 | 4.86E-06 | 949 |
| DAZ2 | 17 | 4.84E-06 | 950 |
| ZNF346 | 139 | 4.84E-06 | 951 |
| RBM19 | 6 | 4.83E-06 | 952 |
| SETD1B | 33 | 4.83E-06 | 953 |
| KIAA0430 | 6 | 4.83E-06 | 954 |
| ESF1 | 7 | 4.83E-06 | 955 |
| SNRNP200 | 72 | 4.82E-06 | 956 |
| SHQ1 | 16 | 4.82E-06 | 957 |
| DDX60 | 16 | 4.82E-06 | 958 |
| ANGEL1 | 4 | 4.82E-06 | 959 |
| DDX60L | 4 | 4.82E-06 | 960 |
| NIFK | 125 | 4.81E-06 | 961 |
| ZNFX1 | 5 | 4.79E-06 | 962 |
| LSM14B | 7 | 4.79E-06 | 963 |
| RNF113B | 2 | 4.79E-06 | 964 |
| PRPF40B | 59 | 4.76E-06 | 965 |
| LSM7 | 16 | 4.76E-06 | 966 |
| RNF113A | 4 | 4.75E-06 | 967 |
| SF3A2 | 40 | 4.74E-06 | 968 |
| METTL14 | 8 | 4.74E-06 | 969 |
| PHRF1 | 37 | 4.72E-06 | 970 |
| CNOT2 | 50 | 4.72E-06 | 971 |
| DAZ4 | 10 | 4.70E-06 | 972 |
| DAZ3 | 15 | 4.69E-06 | 973 |
| BUD13 | 19 | 4.69E-06 | 974 |
| RPS23 | 39 | 4.69E-06 | 975 |
| EIF4A2 | 225 | 4.68E-06 | 976 |
| MRPL47 | 4 | 4.67E-06 | 977 |
| NOP9 | 3 | 4.67E-06 | 978 |
| LCMT2 | 21 | 4.66E-06 | 979 |
| BARD1 | 281 | 4.65E-06 | 980 |
| FRG1B | 2 | 4.64E-06 | 981 |
| MEPCE | 15 | 4.61E-06 | 982 |
| LSM2 | 1000 | 4.61E-06 | 983 |
| DDX59 | 2 | 4.61E-06 | 984 |
| SKIV2L2 | 75 | 4.59E-06 | 985 |
| PAPD7 | 342 | 4.59E-06 | 986 |
| SF3A3 | 39 | 4.56E-06 | 987 |
| DDX24 | 5 | 4.48E-06 | 988 |
| CWF19L2 | 1 | 4.43E-06 | 989 |
| SF3B5 | 1 | 4.43E-06 | 990 |
| ZC3H10 | 1 | 4.43E-06 | 991 |
| LSMD1 | 5 | 4.43E-06 | 992 |
| RNF17 | 20 | 4.42E-06 | 993 |
| ISG20 | 1000 | 4.42E-06 | 994 |
| DHX30 | 6 | 4.41E-06 | 995 |
| NOP10 | 69 | 4.40E-06 | 996 |
| LSM14A | 31 | 4.39E-06 | 997 |
| BRCA1 | 1000 | 4.37E-06 | 998 |
| TUT1 | 48 | 4.37E-06 | 999 |
| CCAR2 | 46 | 4.36E-06 | 1000 |
| WDR46 | 8 | 4.34E-06 | 1001 |
| TNRC6C | 13 | 4.34E-06 | 1002 |
| RPS6 | 998 | 4.32E-06 | 1003 |
| ZC3H8 | 3 | 4.32E-06 | 1004 |
| PRPF39 | 2 | 4.31E-06 | 1005 |
| WDR12 | 29 | 4.31E-06 | 1006 |
| BOLL | 95 | 4.31E-06 | 1007 |
| PAPOLG | 240 | 4.31E-06 | 1008 |
| PABPC1L | 20 | 4.27E-06 | 1009 |
| ZCCHC7 | 21 | 4.27E-06 | 1010 |
| DDX26B | 2 | 4.27E-06 | 1011 |
| POLR2L | 33 | 4.27E-06 | 1012 |
| DXO | 15 | 4.27E-06 | 1013 |
| IGF2BP2 | 230 | 4.26E-06 | 1014 |
| CMTR1 | 2 | 4.26E-06 | 1015 |
| FTO | 1000 | 4.25E-06 | 1016 |
| SNRPN | 536 | 4.23E-06 | 1017 |
| LARP1B | 4 | 4.22E-06 | 1018 |
| EIF4ENIF1 | 36 | 4.22E-06 | 1019 |
| IPO8 | 37 | 4.22E-06 | 1020 |
| TSR3 | 8 | 4.21E-06 | 1021 |
| L1TD1 | 7 | 4.21E-06 | 1022 |
| DGCR8 | 284 | 4.20E-06 | 1023 |
| RNASE11 | 2 | 4.19E-06 | 1024 |
| RNASEH1 | 132 | 4.19E-06 | 1025 |
| DHX35 | 4 | 4.17E-06 | 1026 |
| PAIP2 | 54 | 4.14E-06 | 1027 |
| PPP1R8 | 650 | 4.13E-06 | 1028 |
| WDR5 | 167 | 4.12E-06 | 1029 |
| PPIH | 22 | 4.12E-06 | 1030 |
| EIF2A | 78 | 4.11E-06 | 1031 |
| ZRSR1 | 40 | 4.09E-06 | 1032 |
| DCP1A | 61 | 4.09E-06 | 1033 |
| LSM6 | 12 | 4.07E-06 | 1034 |
| EIF1AY | 25 | 4.07E-06 | 1035 |
| RPL4 | 1000 | 4.06E-06 | 1036 |
| RPL5 | 1000 | 4.06E-06 | 1037 |
| PDCD11 | 35 | 4.06E-06 | 1038 |
| TDRD5 | 10 | 4.05E-06 | 1039 |
| MRTO4 | 3 | 4.05E-06 | 1040 |
| IFIT1B | 2 | 4.04E-06 | 1041 |
| EIF4B | 299 | 4.03E-06 | 1042 |
| KHNYN | 1 | 4.02E-06 | 1043 |
| PAPD4 | 33 | 4.02E-06 | 1044 |
| POLR2K | 24 | 3.99E-06 | 1045 |
| POLR2F | 39 | 3.99E-06 | 1046 |
| GEMIN8 | 7 | 3.99E-06 | 1047 |
| MTRF1 | 181 | 3.98E-06 | 1048 |
| ERI2 | 6 | 3.96E-06 | 1049 |
| APOBEC3G | 969 | 3.95E-06 | 1050 |
| UPF1 | 390 | 3.94E-06 | 1051 |
| NOB1 | 73 | 3.92E-06 | 1052 |
| EIF4E3 | 21 | 3.91E-06 | 1053 |
| RPL8 | 194 | 3.90E-06 | 1054 |
| NOL8 | 4 | 3.90E-06 | 1055 |
| ZC3H12C | 8 | 3.89E-06 | 1056 |
| SNRPD1 | 80 | 3.88E-06 | 1057 |
| DKC1 | 288 | 3.86E-06 | 1058 |
| PIH1D1 | 17 | 3.85E-06 | 1059 |
| GEMIN5 | 29 | 3.85E-06 | 1060 |
| PAIP2B | 5 | 3.85E-06 | 1061 |
| DROSHA | 1000 | 3.85E-06 | 1062 |
| C9orf114 | 1 | 3.84E-06 | 1063 |
| PIH1D2 | 1 | 3.83E-06 | 1064 |
| POLR2H | 39 | 3.83E-06 | 1065 |
| PNRC2 | 14 | 3.83E-06 | 1066 |
| SURF6 | 24 | 3.82E-06 | 1067 |
| MPHOSPH6 | 2 | 3.81E-06 | 1068 |
| CNOT6L | 14 | 3.81E-06 | 1069 |
| FASTKD2 | 10 | 3.78E-06 | 1070 |
| LSM1 | 130 | 3.77E-06 | 1071 |
| TNRC6A | 193 | 3.77E-06 | 1072 |
| RIOK2 | 26 | 3.74E-06 | 1073 |
| RDM1 | 14 | 3.72E-06 | 1074 |
| UBTF | 346 | 3.72E-06 | 1075 |
| PIWIL3 | 11 | 3.72E-06 | 1076 |
| RPS29 | 31 | 3.71E-06 | 1077 |
| AAR2 | 13 | 3.71E-06 | 1078 |
| EIF3L | 11 | 3.68E-06 | 1079 |
| EEF1B2 | 50 | 3.67E-06 | 1080 |
| CNOT6 | 260 | 3.67E-06 | 1081 |
| BOP1 | 54 | 3.67E-06 | 1082 |
| DALRD3 | 1 | 3.65E-06 | 1083 |
| ELAC2 | 105 | 3.65E-06 | 1084 |
| RPS4Y2 | 5 | 3.61E-06 | 1085 |
| MRPL32 | 1 | 3.61E-06 | 1086 |
| RNASEK | 12 | 3.58E-06 | 1087 |
| WDR43 | 5 | 3.58E-06 | 1088 |
| PAPD5 | 10 | 3.57E-06 | 1089 |
| FTSJ3 | 18 | 3.57E-06 | 1090 |
| POLR2E | 75 | 3.56E-06 | 1091 |
| POLR2J | 39 | 3.55E-06 | 1092 |
| PAIP1 | 33 | 3.55E-06 | 1093 |
| RPL32 | 369 | 3.54E-06 | 1094 |
| DAZL | 320 | 3.54E-06 | 1095 |
| NSA2 | 11 | 3.54E-06 | 1096 |
| ETF1 | 552 | 3.54E-06 | 1097 |
| METTL2B | 16 | 3.53E-06 | 1098 |
| NSUN6 | 3 | 3.53E-06 | 1099 |
| UPF3B | 53 | 3.53E-06 | 1100 |
| FASTKD3 | 3 | 3.52E-06 | 1101 |
| FAM98A | 1 | 3.51E-06 | 1102 |
| TDRD10 | 3 | 3.50E-06 | 1103 |
| ZCCHC2 | 2 | 3.49E-06 | 1104 |
| NOL12 | 6 | 3.46E-06 | 1105 |
| RPP25 | 18 | 3.46E-06 | 1106 |
| RPL37A | 53 | 3.46E-06 | 1107 |
| TDRD12 | 6 | 3.46E-06 | 1108 |
| RCL1 | 20 | 3.45E-06 | 1109 |
| TARBP1 | 11 | 3.43E-06 | 1110 |
| DCAF13 | 3 | 3.42E-06 | 1111 |
| TDRD1 | 35 | 3.42E-06 | 1112 |
| PIWIL1 | 787 | 3.42E-06 | 1113 |
| CPSF3 | 48 | 3.42E-06 | 1114 |
| DHX34 | 6 | 3.41E-06 | 1115 |
| MIF4GD | 19 | 3.40E-06 | 1116 |
| NXF2B | 1 | 3.39E-06 | 1117 |
| EIF3B | 107 | 3.39E-06 | 1118 |
| DHX29 | 13 | 3.37E-06 | 1119 |
| PABPC5 | 6 | 3.31E-06 | 1120 |
| CPSF3L | 6 | 3.31E-06 | 1121 |
| ZNF473 | 7 | 3.31E-06 | 1122 |
| SMG9 | 3 | 3.30E-06 | 1123 |
| MRPL23 | 13 | 3.29E-06 | 1124 |
| RPF2 | 15 | 3.29E-06 | 1125 |
| THUMPD1 | 3 | 3.29E-06 | 1126 |
| ZGPAT | 8 | 3.28E-06 | 1127 |
| POLR2B | 410 | 3.27E-06 | 1128 |
| SUPV3L1 | 61 | 3.26E-06 | 1129 |
| ENDOG | 412 | 3.24E-06 | 1130 |
| MRPS30 | 20 | 3.21E-06 | 1131 |
| GTPBP10 | 1 | 3.21E-06 | 1132 |
| MRPS26 | 1 | 3.20E-06 | 1133 |
| NOP58 | 47 | 3.20E-06 | 1134 |
| EIF2S1 | 1000 | 3.19E-06 | 1135 |
| UTP14C | 12 | 3.17E-06 | 1136 |
| MRPL43 | 5 | 3.17E-06 | 1137 |
| RPL7L1 | 2 | 3.15E-06 | 1138 |
| EIF3H | 55 | 3.15E-06 | 1139 |
| DIS3 | 120 | 3.15E-06 | 1140 |
| RNASEL | 690 | 3.14E-06 | 1141 |
| AGO3 | 15 | 3.14E-06 | 1142 |
| PNO1 | 8 | 3.14E-06 | 1143 |
| RSL1D1 | 103 | 3.13E-06 | 1144 |
| EIF3E | 92 | 3.12E-06 | 1145 |
| NOL6 | 11 | 3.11E-06 | 1146 |
| XRN2 | 69 | 3.11E-06 | 1147 |
| EIF4E2 | 31 | 3.10E-06 | 1148 |
| ZNF768 | 1 | 3.10E-06 | 1149 |
| EIF2D | 12 | 3.10E-06 | 1150 |
| MRPL39 | 12 | 3.09E-06 | 1151 |
| EIF4G3 | 61 | 3.09E-06 | 1152 |
| DCP2 | 183 | 3.06E-06 | 1153 |
| GATC | 448 | 3.06E-06 | 1154 |
| RPUSD2 | 1 | 3.05E-06 | 1155 |
| RPL36A | 31 | 3.04E-06 | 1156 |
| NHP2 | 79 | 3.03E-06 | 1157 |
| UTP14A | 5 | 3.02E-06 | 1158 |
| RPL17 | 480 | 3.02E-06 | 1159 |
| RPS2 | 368 | 3.00E-06 | 1160 |
| MRPS21 | 1 | 3.00E-06 | 1161 |
| CNOT4 | 52 | 3.00E-06 | 1162 |
| WDR3 | 6 | 2.99E-06 | 1163 |
| MRPL33 | 3 | 2.98E-06 | 1164 |
| NOA1 | 39 | 2.98E-06 | 1165 |
| EDC3 | 38 | 2.96E-06 | 1166 |
| MRPL55 | 1 | 2.94E-06 | 1167 |
| SNRPD3 | 60 | 2.93E-06 | 1168 |
| POP7 | 16 | 2.90E-06 | 1169 |
| PNPT1 | 369 | 2.88E-06 | 1170 |
| POLR2A | 438 | 2.87E-06 | 1171 |
| TRIM56 | 7 | 2.87E-06 | 1172 |
| UBA52 | 113 | 2.86E-06 | 1173 |
| DIMT1 | 37 | 2.84E-06 | 1174 |
| LSM11 | 20 | 2.84E-06 | 1175 |
| SMG6 | 60 | 2.83E-06 | 1176 |
| MRPS24 | 1 | 2.83E-06 | 1177 |
| SMG7 | 41 | 2.83E-06 | 1178 |
| RPS9 | 88 | 2.83E-06 | 1179 |
| MRPS23 | 3 | 2.81E-06 | 1180 |
| RPS4X | 155 | 2.81E-06 | 1181 |
| GRWD1 | 11 | 2.81E-06 | 1182 |
| PURG | 16 | 2.73E-06 | 1183 |
| RNMTL1 | 14 | 2.72E-06 | 1184 |
| SETD7 | 178 | 2.72E-06 | 1185 |
| MRPL34 | 1 | 2.70E-06 | 1186 |
| RPS27L | 9 | 2.70E-06 | 1187 |
| MRPS18B | 8 | 2.69E-06 | 1188 |
| POLR2G | 100 | 2.69E-06 | 1189 |
| ZCCHC14 | 3 | 2.69E-06 | 1190 |
| UPF2 | 156 | 2.69E-06 | 1191 |
| SMG5 | 39 | 2.68E-06 | 1192 |
| UPF3A | 102 | 2.68E-06 | 1193 |
| CTIF | 12 | 2.68E-06 | 1194 |
| DDX4 | 139 | 2.66E-06 | 1195 |
| INTS10 | 7 | 2.65E-06 | 1196 |
| RNASE8 | 6 | 2.65E-06 | 1197 |
| MRPS18A | 1 | 2.64E-06 | 1198 |
| CMSS1 | 4 | 2.62E-06 | 1199 |
| RBM44 | 3 | 2.62E-06 | 1200 |
| RPL13 | 226 | 2.61E-06 | 1201 |
| DIS3L | 5 | 2.61E-06 | 1202 |
| GAR1 | 65 | 2.60E-06 | 1203 |
| INTS12 | 6 | 2.55E-06 | 1204 |
| PAN2 | 57 | 2.54E-06 | 1205 |
| DDX55 | 1 | 2.54E-06 | 1206 |
| FASTKD1 | 4 | 2.53E-06 | 1207 |
| RPS16 | 254 | 2.51E-06 | 1208 |
| RPP38 | 24 | 2.49E-06 | 1209 |
| IMP4 | 16 | 2.49E-06 | 1210 |
| RPS25 | 32 | 2.49E-06 | 1211 |
| EIF1AX | 57 | 2.47E-06 | 1212 |
| PIWIL4 | 511 | 2.47E-06 | 1213 |
| RPL15 | 329 | 2.45E-06 | 1214 |
| THUMPD2 | 31 | 2.44E-06 | 1215 |
| LENG9 | 1 | 2.41E-06 | 1216 |
| ZNF598 | 1 | 2.40E-06 | 1217 |
| PAN3 | 36 | 2.40E-06 | 1218 |
| EIF2S2 | 374 | 2.39E-06 | 1219 |
| LAS1L | 9 | 2.39E-06 | 1220 |
| RSL24D1 | 45 | 2.37E-06 | 1221 |
| POP4 | 63 | 2.35E-06 | 1222 |
| POP5 | 55 | 2.35E-06 | 1223 |
| RRP12 | 8 | 2.33E-06 | 1224 |
| PUS1 | 39 | 2.32E-06 | 1225 |
| EEF1A1 | 1000 | 2.31E-06 | 1226 |
| DCP1B | 12 | 2.25E-06 | 1227 |
| EIF1 | 281 | 2.24E-06 | 1228 |
| PIH1D3 | 1 | 2.23E-06 | 1229 |
| RPP40 | 7 | 2.20E-06 | 1230 |
| RPP21 | 32 | 2.20E-06 | 1231 |
| RPP30 | 42 | 2.20E-06 | 1232 |
| RRP9 | 9 | 2.18E-06 | 1233 |
| RPL9 | 224 | 2.16E-06 | 1234 |
| TSFM | 266 | 2.16E-06 | 1235 |
| CMTR2 | 1 | 2.16E-06 | 1236 |
| AGO4 | 82 | 2.15E-06 | 1237 |
| POLR2I | 55 | 2.15E-06 | 1238 |
| HENMT1 | 73 | 2.15E-06 | 1239 |
| TRUB2 | 1 | 2.13E-06 | 1240 |
| R3HDM1 | 4 | 2.13E-06 | 1241 |
| EIF6 | 131 | 2.13E-06 | 1242 |
| RPL19 | 267 | 2.11E-06 | 1243 |
| PABPC4L | 1 | 2.08E-06 | 1244 |
| MTPAP | 35 | 2.08E-06 | 1245 |
| NOL9 | 12 | 2.06E-06 | 1246 |
| SRBD1 | 9 | 2.06E-06 | 1247 |
| EIF4E1B | 8 | 2.05E-06 | 1248 |
| SUPT6H | 128 | 2.04E-06 | 1249 |
| LSM10 | 13 | 2.03E-06 | 1250 |
| RRP15 | 1 | 2.03E-06 | 1251 |
| RPS28 | 20 | 2.03E-06 | 1252 |
| METTL10 | 3 | 2.03E-06 | 1253 |
| PUS7 | 7 | 2.03E-06 | 1254 |
| THG1L | 8 | 2.02E-06 | 1255 |
| URM1 | 42 | 2.02E-06 | 1256 |
| SSU72 | 49 | 2.01E-06 | 1257 |
| RPL23 | 261 | 2.01E-06 | 1258 |
| RPL13A | 142 | 2.01E-06 | 1259 |
| RPL37 | 73 | 1.97E-06 | 1260 |
| RPL10 | 744 | 1.97E-06 | 1261 |
| MPHOSPH10 | 26 | 1.93E-06 | 1262 |
| CSTF2T | 13 | 1.92E-06 | 1263 |
| VARS | 83 | 1.92E-06 | 1264 |
| DDX51 | 2 | 1.92E-06 | 1265 |
| SETD1A | 318 | 1.91E-06 | 1266 |
| ADAT2 | 35 | 1.89E-06 | 1267 |
| DENR | 210 | 1.88E-06 | 1268 |
| PPRC1 | 22 | 1.88E-06 | 1269 |
| TRDMT1 | 126 | 1.88E-06 | 1270 |
| RPL36 | 114 | 1.86E-06 | 1271 |
| RPL41 | 102 | 1.82E-06 | 1272 |
| MRPL13 | 36 | 1.82E-06 | 1273 |
| MRRF | 223 | 1.82E-06 | 1274 |
| MRPL48 | 4 | 1.81E-06 | 1275 |
| RNASE13 | 2 | 1.80E-06 | 1276 |
| POLR1E | 24 | 1.77E-06 | 1277 |
| PCF11 | 54 | 1.76E-06 | 1278 |
| ISG20L2 | 3 | 1.76E-06 | 1279 |
| MRPL4 | 3 | 1.75E-06 | 1280 |
| SUPT4H1 | 97 | 1.74E-06 | 1281 |
| PUS3 | 17 | 1.74E-06 | 1282 |
| NSUN2 | 51 | 1.73E-06 | 1283 |
| MTO1 | 51 | 1.73E-06 | 1284 |
| MRPL41 | 16 | 1.71E-06 | 1285 |
| RPS18 | 114 | 1.68E-06 | 1286 |
| MRPL38 | 6 | 1.67E-06 | 1287 |
| RPS19 | 364 | 1.67E-06 | 1288 |
| RPL7 | 622 | 1.67E-06 | 1289 |
| RPL6 | 39 | 1.67E-06 | 1290 |
| RPL18 | 353 | 1.67E-06 | 1291 |
| SNRPG | 1 | 1.65E-06 | 1292 |
| MRPL40 | 12 | 1.64E-06 | 1293 |
| EIF4E | 1000 | 1.63E-06 | 1294 |
| EIF3G | 30 | 1.61E-06 | 1295 |
| RPL7A | 81 | 1.61E-06 | 1296 |
| UTP18 | 8 | 1.61E-06 | 1297 |
| PLD6 | 98 | 1.58E-06 | 1298 |
| POLR2J3 | 1 | 1.53E-06 | 1299 |
| MRPS11 | 6 | 1.53E-06 | 1300 |
| KIAA0391 | 16 | 1.52E-06 | 1301 |
| MRPL30 | 1 | 1.48E-06 | 1302 |
| HEATR1 | 4 | 1.48E-06 | 1303 |
| GTF2F1 | 269 | 1.48E-06 | 1304 |
| SUPT5H | 165 | 1.47E-06 | 1305 |
| RTF1 | 57 | 1.47E-06 | 1306 |
| RPL14 | 280 | 1.47E-06 | 1307 |
| EIF3C | 38 | 1.46E-06 | 1308 |
| EIF5B | 348 | 1.45E-06 | 1309 |
| MRPL20 | 4 | 1.45E-06 | 1310 |
| EPRS | 334 | 1.45E-06 | 1311 |
| TEFM | 6 | 1.44E-06 | 1312 |
| YTHDF3 | 1 | 1.38E-06 | 1313 |
| FTSJ2 | 12 | 1.34E-06 | 1314 |
| RPL39L | 8 | 1.32E-06 | 1315 |
| UTP23 | 6 | 1.32E-06 | 1316 |
| PTCD2 | 3 | 1.29E-06 | 1317 |
| TRNAU1AP | 9 | 1.28E-06 | 1318 |
| YARS | 229 | 1.28E-06 | 1319 |
| EIF5 | 178 | 1.27E-06 | 1320 |
| BCDIN3D | 6 | 1.24E-06 | 1321 |
| METTL1 | 19 | 1.22E-06 | 1322 |
| TRMT1 | 50 | 1.22E-06 | 1323 |
| MRPL16 | 2 | 1.19E-06 | 1324 |
| TRPT1 | 2 | 1.17E-06 | 1325 |
| RPS14 | 218 | 1.16E-06 | 1326 |
| EEFSEC | 96 | 1.15E-06 | 1327 |
| TFAM | 1000 | 1.09E-06 | 1328 |
| POLRMT | 46 | 1.09E-06 | 1329 |
| TFB1M | 67 | 1.08E-06 | 1330 |
| MRPL12 | 18 | 1.08E-06 | 1331 |
| TRMT10A | 12 | 1.07E-06 | 1332 |
| TRMT10C | 7 | 1.07E-06 | 1333 |
| GPATCH1 | 1 | 1.07E-06 | 1334 |
| MRPL3 | 12 | 9.91E-07 | 1335 |
| ZC3H4 | 2 | 9.30E-07 | 1336 |
| TRMT6 | 19 | 9.27E-07 | 1337 |
| TRMT61A | 20 | 9.26E-07 | 1338 |
| TRMT5 | 21 | 9.26E-07 | 1339 |
| TFB2M | 71 | 9.21E-07 | 1340 |
| RPL22 | 406 | 9.05E-07 | 1341 |
| RARS2 | 161 | 9.03E-07 | 1342 |
| RPL24 | 232 | 8.94E-07 | 1343 |
| MRPS15 | 2 | 8.71E-07 | 1344 |
| RPL29 | 256 | 8.70E-07 | 1345 |
| MRM1 | 6 | 8.68E-07 | 1346 |
| RPL11 | 703 | 8.67E-07 | 1347 |
| RPL21 | 139 | 8.66E-07 | 1348 |
| RPL27 | 261 | 8.65E-07 | 1349 |
| RPL31 | 153 | 8.59E-07 | 1350 |
| RPL10L | 3 | 8.43E-07 | 1351 |
| CNOT10 | 3 | 7.81E-07 | 1352 |
| RBM43 | 2 | 7.80E-07 | 1353 |
| MRPS34 | 8 | 7.77E-07 | 1354 |
| NOL11 | 2 | 7.74E-07 | 1355 |
| WDR4 | 14 | 7.74E-07 | 1356 |
| NSUN4 | 6 | 7.67E-07 | 1357 |
| RPS11 | 53 | 7.34E-07 | 1358 |
| RPL35 | 139 | 7.26E-07 | 1359 |
| RPL28 | 107 | 7.21E-07 | 1360 |
| RPL30 | 221 | 7.17E-07 | 1361 |
| RPL39 | 60 | 7.11E-07 | 1362 |
| RPL36AL | 12 | 7.09E-07 | 1363 |
| MRPS33 | 3 | 7.03E-07 | 1364 |
| TRMT112 | 17 | 6.73E-07 | 1365 |
| EIF3J | 23 | 6.40E-07 | 1366 |
| MRPL10 | 7 | 6.36E-07 | 1367 |
| MRPS12 | 14 | 6.32E-07 | 1368 |
| MRPL11 | 10 | 6.24E-07 | 1369 |
| MTIF3 | 23 | 6.22E-07 | 1370 |
| PTCD1 | 5 | 6.22E-07 | 1371 |
| MRPL44 | 4 | 6.16E-07 | 1372 |
| DUS1L | 2 | 6.13E-07 | 1373 |
| TARSL2 | 2 | 6.08E-07 | 1374 |
| DARS2 | 275 | 6.08E-07 | 1375 |
| TYW5 | 3 | 5.99E-07 | 1376 |
| RPS12 | 310 | 5.98E-07 | 1377 |
| MRPL28 | 5 | 5.97E-07 | 1378 |
| MRPL14 | 4 | 5.96E-07 | 1379 |
| RPS5 | 138 | 5.72E-07 | 1380 |
| RPS15 | 143 | 5.68E-07 | 1381 |
| RPL12 | 448 | 5.67E-07 | 1382 |
| RPS10 | 116 | 5.59E-07 | 1383 |
| RPS24 | 55 | 5.56E-07 | 1384 |
| RPS26 | 47 | 5.56E-07 | 1385 |
| RPS21 | 44 | 5.55E-07 | 1386 |
| RPL3L | 3 | 5.55E-07 | 1387 |
| RPL35A | 61 | 5.54E-07 | 1388 |
| RPS17 | 70 | 5.53E-07 | 1389 |
| RPL22L1 | 4 | 5.53E-07 | 1390 |
| RPS20 | 83 | 5.52E-07 | 1391 |
| PTRHD1 | 1 | 4.89E-07 | 1392 |
| PTCD3 | 5 | 4.80E-07 | 1393 |
| MRPL53 | 2 | 4.76E-07 | 1394 |
| MRPL2 | 7 | 4.73E-07 | 1395 |
| MTIF2 | 25 | 4.72E-07 | 1396 |
| MTRF1L | 12 | 4.71E-07 | 1397 |
| MRPS16 | 4 | 4.70E-07 | 1398 |
| MRPS22 | 17 | 4.66E-07 | 1399 |
| PARS2 | 64 | 4.65E-07 | 1400 |
| MRPS9 | 4 | 4.45E-07 | 1401 |
| MRPS35 | 10 | 4.26E-07 | 1402 |
| MRPS10 | 5 | 4.22E-07 | 1403 |
| RPS13 | 92 | 4.21E-07 | 1404 |
| RPS7 | 172 | 4.20E-07 | 1405 |
| RPS3 | 252 | 4.20E-07 | 1406 |
| RPS8 | 108 | 4.17E-07 | 1407 |
| MRPS2 | 3 | 4.10E-07 | 1408 |
| MRPL15 | 2 | 4.07E-07 | 1409 |
| ADAT3 | 18 | 3.38E-07 | 1410 |
| ALKBH8 | 11 | 3.22E-07 | 1411 |
| MRPL37 | 5 | 3.21E-07 | 1412 |
| EARS2 | 310 | 3.20E-07 | 1413 |
| HARS2 | 236 | 3.17E-07 | 1414 |
| DUS2 | 7 | 3.16E-07 | 1415 |
| KARS | 508 | 3.15E-07 | 1416 |
| AARS | 270 | 3.15E-07 | 1417 |
| TRMT61B | 2 | 3.14E-07 | 1418 |
| TYW3 | 4 | 3.13E-07 | 1419 |
| QARS | 232 | 3.11E-07 | 1420 |
| AARS2 | 180 | 3.11E-07 | 1421 |
| YARS2 | 379 | 3.09E-07 | 1422 |
| CARS | 91 | 3.08E-07 | 1423 |
| LARS2 | 283 | 3.07E-07 | 1424 |
| FARSB | 13 | 3.06E-07 | 1425 |
| MARS | 366 | 3.05E-07 | 1426 |
| TRMT44 | 3 | 3.05E-07 | 1427 |
| MRPS17 | 4 | 3.05E-07 | 1428 |
| MRPL24 | 3 | 3.01E-07 | 1429 |
| MRPL18 | 2 | 2.95E-07 | 1430 |
| MRPS14 | 2 | 2.94E-07 | 1431 |
| MRPL1 | 2 | 2.90E-07 | 1432 |
| MRPL51 | 4 | 2.68E-07 | 1433 |
| ADAT1 | 11 | 1.99E-07 | 1434 |
| MRPL36 | 11 | 1.91E-07 | 1435 |
| TUFM | 1000 | 1.85E-07 | 1436 |
| PUS10 | 21 | 1.81E-07 | 1437 |
| TRUB1 | 15 | 1.78E-07 | 1438 |
| TYW1 | 12 | 1.73E-07 | 1439 |
| MRPS27 | 4 | 1.71E-07 | 1440 |
| MRPS36 | 5 | 1.70E-07 | 1441 |
| PET112 | 7 | 1.69E-07 | 1442 |
| SARS2 | 716 | 1.69E-07 | 1443 |
| MRPL42 | 4 | 1.66E-07 | 1444 |
| PSTK | 34 | 1.66E-07 | 1445 |
| MRPS5 | 2 | 1.65E-07 | 1446 |
| TARS | 414 | 1.64E-07 | 1447 |
| MRPL22 | 3 | 1.64E-07 | 1448 |
| MRPL50 | 2 | 1.64E-07 | 1449 |
| MRPL9 | 3 | 1.64E-07 | 1450 |
| NARS2 | 24 | 1.63E-07 | 1451 |
| MARS2 | 110 | 1.62E-07 | 1452 |
| **NARS** | 121 | 1.61E-07 | 1453 |
| MRPL27 | 3 | 1.61E-07 | 1454 |
| DARS | 95 | 1.61E-07 | 1455 |
| VARS2 | 261 | 1.60E-07 | 1456 |
| QTRTD1 | 4 | 1.59E-07 | 1457 |
| CARS2 | 20 | 1.58E-07 | 1458 |
| IARS2 | 265 | 1.58E-07 | 1459 |
| FARS2 | 414 | 1.58E-07 | 1460 |
| WARS2 | 120 | 1.58E-07 | 1461 |
| TARS2 | 31 | 1.57E-07 | 1462 |
| **WARS** | 368 | 1.57E-07 | 1463 |
| MRPL46 | 1 | 1.55E-07 | 1464 |
| TRMT11 | 9 | 5.12E-08 | 1465 |
| TRMT12 | 8 | 2.01E-08 | 1466 |
| **QTRT1** | 113 | 1.32E-08 | 1467 |

All candidate proteins are listed using the HGNC database of human gene names and protein coding genes. Abstracts used by Watson for analysis are listed for each protein. Proteins selected for validation studies are noted in bold. GD = Graph diffusion score assigned by Watson to each protein based on semantic similarity of the candidate to the whole positive training set.

**Table S5**- **Subject demographics.**

| **Case ID** | **Group** | **Gender** | **Age** | **Site of onset** | **PMI (hrs)** |
| --- | --- | --- | --- | --- | --- |
| 1 | CON | M | 75 | - | 10 |
| 2 | CON | M | 82 | - | 5 |
| 3 | CON | M | 74 | - | 4.5 |
| 4 | CON | F | 72 | - | 9 |
| 5 | CON | M | 53 | - | 5 |
| 6 | CON | F | 59 | - | 6 |
| 7 | CON | F | 51 | - | 5 |
| 8 | CON | F | 57 | - | 11 |
| 9 | CON | M | 57 | - | 2 |
| 10 | CON | M | 54 | - | 6 |
| 11 | CON | F | 53 | - | 4 |
| 12 | CON | M | 48 | - | 2 |
| 13 | CON | F | 58 | - | 5 |
| 14 | CON | M | 76 | - | 13 |
| 15 | CON | M | 86 | - | N/A |
| 16 | CON | M | 78 | - | N/A |
| 17 | CON | F | 85 | - | N/A |
| 18 | CON | M | 82 | - | 17 |
| 19 | C9-ALS | M | 43 |  | 6 |
| 20 | C9-ALS | M | 68 |  | 4 |
| 21 | C9-ALS | F | 63 | Limb | 4 |
| 22 | C9-ALS | M | 67 | Limb | 4 |
| 23 | C9-ALS | F | 61 |  | 5.5 |
| 24 | ALS | M | 69 |  | 5 |
| 25 | ALS | F | 72 |  | 9 |
| 26 | ALS | M | 53 |  | 5 |
| 27 | ALS | M | 53 |  | N/A |
| 28 | ALS | F | 73 |  | 11 |
| 29 | ALS | F | 62 |  | 4 |
| 30 | ALS | M | 69 |  | 10 |
| 31 | ALS | F | 76 |  | 6 |
| 32 | ALS | F | 63 | Limb | 5 |
| 33 | ALS | M | 45 |  |  |
| 34 | ALS | M | 55 | Limb | 14 |
| 35 | ALS | M | 45 |  | 3 |
| 36 | ALS | M | 51 |  | 4 |
| 37 | ALS | M | 45 | Bulbar | 7 |
| 38 | ALS | F | 68 |  | 1 |
| 39 | ALS | F | 76 | Limb | 8 |
| 40 | ALS | F | 69 |  | 7.5 |
| 41 | ALS | F | 66 | Bulbar | 9 |
| 42 | ALS | F | 81 | Limb | N/A |
| 43 | ALS | M | 85 | Limb | 7.5 |
| 44 | ALS | F | 61 | Limb | 3.5 |
| 45 | ALS | F | 63 | Limb | 37.5 |
| 46 | ALS | F | 68 |  | 6 |
| 47 | ALS | M | 74 |  | 7 |
| 48 | ALS | M | 67 |  | 7.5 |
| 49 | ALS | M | 74 | Limb | 3.5 |
| 50 | ALS | F | 60 |  | 4 |
| 51 | ALS | M | 39 |  | 5.5 |
| 52 | FTLD-Tau | M | 69 |  | 6 |
| 53 | FTLD-TDP | M | 85 |  | 3 |

PMI = post-mortem interval; CON = Non-neurologic disease control; C9-ALS = ALS with C9ORF72 repeat expansion; FTLD-Tau = frontotemporal lobar degeneration with tau pathology; FTLD-TDP = frontotemporal lobar degeneration with TDP-43 pathology; N/A: Not available. All diagnoses were performed by licensed neuropathologists.

| **Antibody** | **Company** | **Catalog Number** | **Lot number** |
| --- | --- | --- | --- |
| SRSF2/SC-35 | Abcam | ab11826 | GR15177-3 |
| SRSF2/SC-35 | Abcam | ab204916 | GR154517-18 |
| Caprin-1 | Proteintech | 15112-1-AP | 26073 |
| NUPL2 | Abcam | ab192609 | GR182138-2 |
| RBMS3 | Abcam | ab198248 | GR208427-6 |
| RBMS3 | Abcam | ab51504 | GR121957-8 |
| RBM6 | Proteintech | 14360-1-AP | 5405 |
| Syncrip | Sigma-Prestige | HPA041275 | R37386 |
| hnRNPU | Abcam | ab10297 | GW32083-35 |
| hnRNPH2 | Abcam | ab181171 | GR164588-1 |
| QTRT1 | Sigma-Prestige | HPA048651 | R58620 |
| NARS | Sigma-Prestige | HPA040017 | R36651 |
| WARS | Bethyl Labs | A304-274A | None listed |
| Parvalbumin | Millipore | MAB1572 | 2788952 |
| Calretinin | R&D systems | AF5065-SP | CBJR011604A |
| GFAP | Sigma | G6171 | 049K4788 |
| p62 lck ligand /sqstm1 | BD Transduction Laboratories | 610833 | 6120781 |
| TDP-43 | Proteintech | 10782-2-AP | 21882 |

**Table S6**. **List of antibodies used in the study.**

**Table S7. List of primers used.**

| **Primer Name** | **Sequence (5'-3')** |
| --- | --- |
| h-hnRNPU-For | TCCATTGTAAGGGAAGAAGCA |
| h-hnRNPU-Rev | TTCGATGACACAGTGGTTTG |
| h-hnRNPH2-For | CTTCCGTGCTCAGCATCAT |
| h-hnRNPH2-Rev | GGAAAATTACCCGGTATCGTTAG |
| h-RBMS3-For | GTGGGGAGCTGGTGCATA |
| h-RBMS3-Rev | ATTCCAGCTACATGGGCAAA |
| h-Caprin1-For | TCCCCTTTGTTCATTCGTTC |
| h-Caprin1-Rev | AGGCCATGAAGCAGATTCTC |
| h-NUPL2-For | AGCCTCAATTCCTCTGGTGA |
| h-NUPL2-Rev | AGGGCAGTGGATGTTTTCTG |
| h-RBM6-For | TCTTGGGCGGCAAGACA |
| h-RBM6-Rev | GGCTTCCTGGCAGCCTATG |
| h-SRSF2-For | GTA AGT ACG TGC CTG AAA CT |
| h-SRSF2-Rev | TTA CAC TGC TTG CCG ATA C |
| h-hnRNPQ-For | TGCATTGGCAGTTCTTCAAC |
| h-hnRNPQ-Rev | TCTGCTACTTTGGTCCCTTG |

**Table S8.** iPSC lines demographics

| **Cell line ID** | **Gender** | **Age at Biopsy** | **Diagnosis** | **Estimated C9orf72 Repeat Length** | **Age at Death** |
| --- | --- | --- | --- | --- | --- |
| CON-1 | Female | 35 | Control | <30 | N/A |
| CON-2 | Male | 76 | Control | <30 | N/A |
| CON-3 | Male | N/A | Control; (MCT8 deficiency, clinically normal) | <30 | N/A |
| SALS1 | Male | 44 | Sporadic-ALS | <30 | 52 |
| SALS2 | Male | 68 | Sporadic-ALS | <30 | 68 |
| C9-ALS1 | Male | 58 | C9orf72-fALS | 1100 | 58 |
| C9-ALS2 | Male | 58 | C9orf72-sALS | >30 | 59 |
| C9-ALS3 | Male | 54 | C9orf72-fALS | 1150 | 58 |
| C9-ALS4 | Male | 65 | C9orf72-sALS | >620 | N/A |
| C9-ALS5 | Male | 51 | C9orf72-ALS | >30 | 53 |

N/A: Not available. CON = Non-neurologic disease control; C9-ALS = ALS with C9ORF72 repeat expansion. SALS=Sporadic ALS. All diagnoses were performed by licensed neuropathologists.

**Figure S1.** Immunohistochemical analysis of lumbar spinal cord sections, stained for **a)** Syncrip, **b)** RBMS3, **c)** hnRNPH2 and **d)** NUPL2. For each of these RBPs, 4 control, 2-4 C9-ALS and 4-6 SALS spinal cord tissue samples were analyzed. Variability in staining patterns within the control and ALS groups made it hard to obtain meaningful conclusions pertaining to staining intensities in ALS. **a)** Syncrip was localized to the nuclear compartment in controls, however some control motor neurons exhibited cytoplasmic staining. **b)** RBMS3 was strong in the cytoplasmic compartment of control motor neurons, while ALS samples showed strong sometimes darker cytoplasmic staining. Some punctate neuropil staining could be occasionally seen, with strong RBMS3 staining in the grey matter. **c)** No differences in hnRNPH2 staining was observed between control and ALS groups. **d)** NUPL2 was detected in astrocytes in all ALS samples (4 out of 4 SALS), while one SALS sample showed nucleolar localization. Scale bar depicts 50μm.

**Figure S2**. Cerebellum sections were stained for hnRNPH2 **a)**, SC-35 **b)**, hnRNPU **c)** and RBM6 **d).** **a)** No differences in hnRNPH2 staining were observed between control and ALS. **b)** SC-35 nuclear speckles were found in Purkinje neuron nuclei, with occasional darker and larger speckles in ALS samples. **c)** hnRNPU staining was negative to weak in control (4 out of 4 cases), some SALS samples (3 out of 7 SALS cases) and 1 C9-ALS case (out of 5 cases). hnRNPU staining was medium to strong in 4 out of 5 C9-ALS and 4 out of 7 SALS. **d)** RBM6 in control cerebellum was very faint, while in ALS samples we could occasionally detect RBM6 in interneurons. Scale bar: 50μm.

**Figure S3. a)** Frontal cortex samples from 3 controls, 2 SALS, 1 C9-ALS and 2 FTLD cases were stained for SC-35. Representative images with neuropil staining and inclusions are shown. Scale bar 50μm. **b)** Staining for NUPL2 in ALS cerebellum showing astrocytic staining in the granular, molecular and white matter cell layers. Scale bar: 100μm.

**Fig ure S4**. Immunohistochemical characterization of bottom-ranked IBM Watson RBP proteins NARS, WARS and QTRT1 in ALS and control cerebellum **a)** and lumbar spinal cords **b).** No differences were detected between control and ALS groups for any of these proteins. Scale bar: 50μm.

**Extended Materials and Methods**

**RBP Ranking Methodology**

The problem of rank-ordering a set of candidates based on a small set of positive training examples combines the challenges of semi-supervised learning [[66](#_ENREF_66)] and one-class classification [[42](#_ENREF_42)]. The solution must obtain a general pattern from a small set of specific examples, without becoming overly specific to those examples. In other words, the goal is not only to rank candidates highly when they are similar to individual inputs, but also when they are related to the general pattern of all inputs taken as a whole. This is not a problem that has been heavily studied to date, but it is an important one in hypothesis generation, because positive examples are often few, and published negative examples, even rarer.

To rank-order all RBPs for their predicted involvement in ALS, we took a literature mining approach that was already successfully applied to predict protein-protein interactions [[55](#_ENREF_55)]. This approach is based on the premise that proteins discussed in similar textual contexts may have similar functions. The approach is composed of two steps: First, we use text mining to compute the semantic similarity of every RBP to every other RBP. Secondly, we use a network analysis to rank all unknown RBPs for semantic similarity to the overall set of 11 RBPs known to be involved in ALS.

1. **IBM Watson Text Mining to Determine Semantic Similarity Between RBPs**

To determine semantic similarity between all RBPs, we analyze the language used around them in the literature. There are 1,542 RBPs in the human genome [[23](#_ENREF_23)]. Each of these proteins can have tens of synonyms, and in order to capture all the mentions of these genes/proteins, we rely on Watson’s custom thesaurus. The thesaurus lists all genes/proteins in the human genome along with their canonical name (or main name), and synonyms aggregated from genecards.org, UNIPROT and NCBI’s UniGene [[46](#_ENREF_46),[55](#_ENREF_55)].

Recognizing correct protein mentions and attributing them to the appropriate canonical class poses several challenges: First, one name can be a synonym for multiple types of proteins. Second, a protein synonym can be an abbreviation for something else depending on context. An illustrative example of the first challenge is the RBP RBMY1A1 that has 14 synonyms in Watson’s thesaurus. Two of those synonyms are also synonyms for different genes (RBM1 for RBMXL1, and YYRM2 for RBMY1F). In order to disambiguate these synonym mentions and assign them the proper canonical class, Watson uses both a context model for each protein, along with specific rules for certain proteins. The context model for each protein is constructed from the 100 most relevant abstracts that mention the synonym name (relevance is established by the TF-IDF score within the Solr system [[43](#_ENREF_43)]). Given an ambiguous protein mention (one that is a synonym of multiple canonical classes), Watson uses the context model to compute a score for each candidate canonical name, with the highest scoring canonical protein selected in a manner similar to query likelihood models in information retrieval [[43](#_ENREF_43)]. Since the meaning of mentions of a given phrase is usually consistent within a piece of text [[21](#_ENREF_21)], the context model is applied at the document level: when the same protein name occurs multiple times in a document, the highest scoring canonical protein is selected consistently throughout that document. In addition to this context model, Watson also uses curated lists of “ambiguous” terms that are given a lower probability of being annotated depending on context.

An illustrative example of the second challenge is the RBP EWSR1, which has EWS as one of its synonyms. EWS is also an acronym for Ewing’s Sarcoma. In order to distinguish between the mention of the protein and the mention of the disease, Watson uses both the context and a custom acronym annotator component.

Using Watson’s methodology, we queried all abstracts in PubMed^®^ for those that mention any of the RBPs used in this study. 1,478 RBPs had at least one mention up to the end of 2015. For each protein, we downloaded up to 1,000 abstracts mentioning it. We previously determined that including more than 1,000 abstracts per protein does not increase model performance [[55](#_ENREF_55)]. For proteins associated with more than 1,000 abstracts, abstracts were randomly selected except that preference was given for documents that mention the protein the most number of times. This resulted in a total of 247,078 abstracts for the 1,478 RBPs. PubMed IDs for all RBPs used for this analysis are listed in Supplemental Table 2.

Next, every abstract was turned into a mathematical representation of its vocabulary, using the bag-of-words and vector space models. A dictionary of terms (or feature space) was built from the most frequent words and phrases used in these abstracts. In the first phase of dictionary preparation, all abstracts were scanned and the 25,000 words appearing in the most number of documents were selected. These words do not include terms belonging to a standard stop-words list, which is used to eliminate irrelevant words such as “and” or “with”. In the second phase of dictionary preparation, we selected phrases that were comprised of words from the 25,000 list. A phrase is defined as a sequence of two words not separated by non-stop words. The dictionary (feature space) was then once again trimmed to include only the 25,000 most frequent words and phrases, listed in Supplemental Table 3. For each abstract, we counted the number of occurrences of every term included in the dictionary. To avoid penalizing rare terms, we normalized each term frequency by its occurrence in the overall document set, using a term frequency–inverse document frequency weighing scheme (TF-IDF) weighing scheme [[53](#_ENREF_53)].

Each vector was then normalized using Euclidian distance to give it a magnitude of 1. This led to a vector representation of every abstract, where the features are the terms in the dictionary and the values are the weighted frequencies in the document.

For each protein, we averaged the vectors of the abstracts associated with it to create a single vector representation, or centroid. Every protein centroid was then compared to every other centroid in a distance matrix. Values in this symmetrical distance matrix corresponded to the cosine distance between the centroid vectors of every protein pair (Supplemental Table 4). This denotes the similarity of each protein pair, in terms of the words and phrases used around them. The advantage of this bag-of-words approach is that it is highly unbiased: proteins do not need to ever be mentioned together for them to be scored as highly similar. Instead, two proteins are similar to each other if the words and phrases used in documents that mention them are similar.

1. **Ranking for Similarity to a Known Set**

For the prospective analysis, in order to rank each of the 1,467 candidate RBP for semantic similarity to the set of 11 RBPs mutated and known to be implicated in ALS (the positive set), we use a network analysis algorithm, graph diffusion [[65](#_ENREF_65)]. In our case, the graph is a network of nodes (the RBPs, positives and candidates) with edges taken from the semantic distance matrix. Graph diffusion assigns a score to every RBP, by starting out with all known positive nodes having the initial temperature 1 and all candidates having the temperature 0. The algorithm then propagates heat along the connected network until a point of stability is reached. The final temperature of each candidate becomes its score. For more details about the algorithm, refer to the Supplementary code file and Spangler et al. [[55](#_ENREF_55)]. For every RBP, this results in a score from 0 to 1, corresponding to how closely related it is to the overall set of 11 positive RBPs. While the raw values of the candidate scores are not reflective of likelihood, we considered the relative ranks of candidate RBPs as indicative of relative similarity of candidates to the original positive set as a whole.

**Immunofluorescence**

Paraffin-embedded post-mortem tissue sections from spinal cords and cerebellum were used for this study. All sections were deparaffinized, rehydrated and antigen retrieval performed using Target Antigen Retrieval Solution, pH 9.0 (DAKO) or a citrate buffer (pH 6) for 20 min in a steamer. After cooling to room temperature, non-specific binding sites were blocked using Super Block (Scytek) for 1 h. Primary antibodies were incubated overnight in Antibody Diluent with background reducing component (Dako). Slides were subsequently washed and incubated for 1 h in the appropriate fluorescent secondary antibody (Alexafluor, Thermo Fisher Scientific). Slides were washed in PBS for 45 minutes, autofluorescence eliminator reagent (EMD Millipore) was applied for 5 min, slides were washed in 70% Ethanol and mounted using Prolong Diamond Antifade Mountant with DAPI (ThermoFisher). Slides were imaged on the Zeiss LSM 710 confocal microscope.

**Western blot analysis**

Lumbar spinal cord and cerebellum total tissue homogenates were prepared from frozen tissue from controls and ALS cases for western blot analysis. Total cell lysates were prepared by homogenizing samples in 25mM HEPES pH7.9, 50mM NaCl, 1% Triton X-100, supplemented with protease and phosphatase inhibitors as previously described [[32](#_ENREF_32)]. Lysates were subsequently spun at 14,000rpm for 10min, and supernatants were used for western blot analysis using Nupage Bis-Tris 4-12% protein gels from Invitrogen. 30ug of proteins were mixed with LDS Nupage sample buffer, boiled at 70^0^C for 10 min, loaded and ran at 100V for 1h30 min. Gels were transferred onto Immobilon FL (Millipore) PVDF membranes, blocked in Odyssey blocking buffer, and probed with primary and secondary antibodies. Signals were imaged using the Odyssey CLx Imager (LiCor), and densitometric analysis was performed using the ImageStudio 4.0 software from LiCor. All antibodies used generated bands at the appropriate size and are listed in Suppl. Table 6.

**Human iPSC culture**

Human induced pluripotent stem cells (iPSCs) were generated under institutional IRB consent from Johns Hopkins University, Baltimore, MD (Drs. Rothstein and Maragakis), Cedar Sinai, Los Angeles, CA (Drs. Sareen and Svendsen) and Research Programs Unit, Molecular Neurology and Stem Cell Center at the University of Helsinki, Helsinki, Finland (Drs. Tienari and Otonkoski) and generously provided to us following institutional material transfer agreement. The use of these human cell lines has been approved by the St. Joseph’s Hospital and Medical Center IRB. Human iPSC were cultured and maintained with MTSER (StemCell Technologies) and evaluated for pluripotency by quantitative PCR and immunofluorescence. Briefly, during the neuralization stage, cells are cultured in WiCell Medium (DMEM/F12, knockout serum replacement, 1% L-Glutamine, 1% NEAA, 110μM B-ME) supplemented with 0.5μM LDN (Stemgent) and 10μM SB (Sigma) for BMP and SMAD signaling inhibition. Once cells enter the caudalization stage, they are cultured in 50% WiCell: 50% Neural Induction Media (NIM: DMEM/F12, 1% L-Glutamine, 1% NEAA, 1% N2, 1% Pen/Strep and 2μg/ml heparin) and supplemented with 0.5μM LDN, 10μM SB and 0.5μM retinoic acid (RA, Sigma). During ventralization, cells are maintained in NIM supplemented with 0.5μM RA, 200ng/ml SHH-C (Peprotech), 10ng/ml BDNF (Invitrogen) and 0.4 μg/ml ASAC (Sigma). At the stage of neural progenitor cell differentiation, cells are switched to a 50% NIM: 50% Neural Differentiation Media (NDM: Neurobasal, 1% L-Glutamine, 1% NEAA, 1% N2, 1% Pen/Strep) supplemented with the following components: 0.5μM RA, 200ng/ml SHH-C, 0.4 μg/ml ASAC, 2% B27, 10ng/ml BDNF (Invitrogen), 10ng/ml GDNF, 10ng/ml IGF and 10ng/ml CNTF (all from R&D systems). Cells are then switched to 100% NDM. The culture is treated with 20 mM  AraC  (Sigma)  for  48  h  to  remove  glial progenitors  cells. Using the aforementioned protocol, we obtained iPSC motor neurons that are positive for Tuj-1 neuronal marker and 30-40% of the cells are positive for the motor neuron marker HB9 as previously described [[16](#_ENREF_16)]. Cells are considered mature at day 32-36 of the motor differentiation protocol, and for our experiments we differentiated the cells between 50-60 days. iPSC demographics are found in Suppl. Table 8.
