# Supplementary figures and images for: Artificial intelligence in neurodegenerative disease research: use of IBM Watson to identify additional RNA-binding proteins altered in amyotrophic lateral sclerosis
Source: Acta Neuropathol. 2017 Nov 13;135(2):227–47. doi: 10.1007/s00401-017-1785-8 (PMC5773659; doi:10.1007/s00401-017-1785-8)

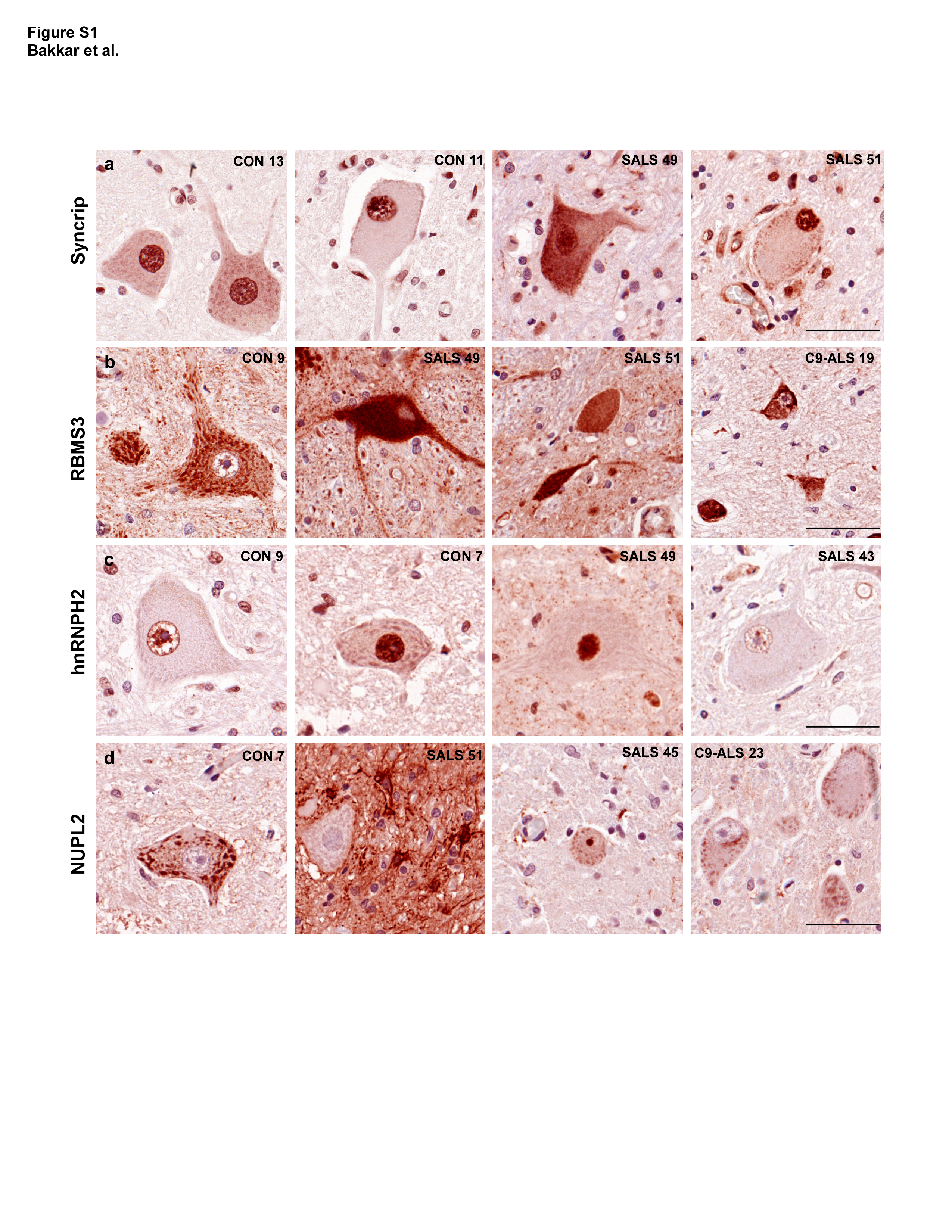

Supplement: Supplementary file 2 — Supplementary material 2 (TIFF 14290 kb) [file 401_2017_1785_MOESM2_ESM.tif]

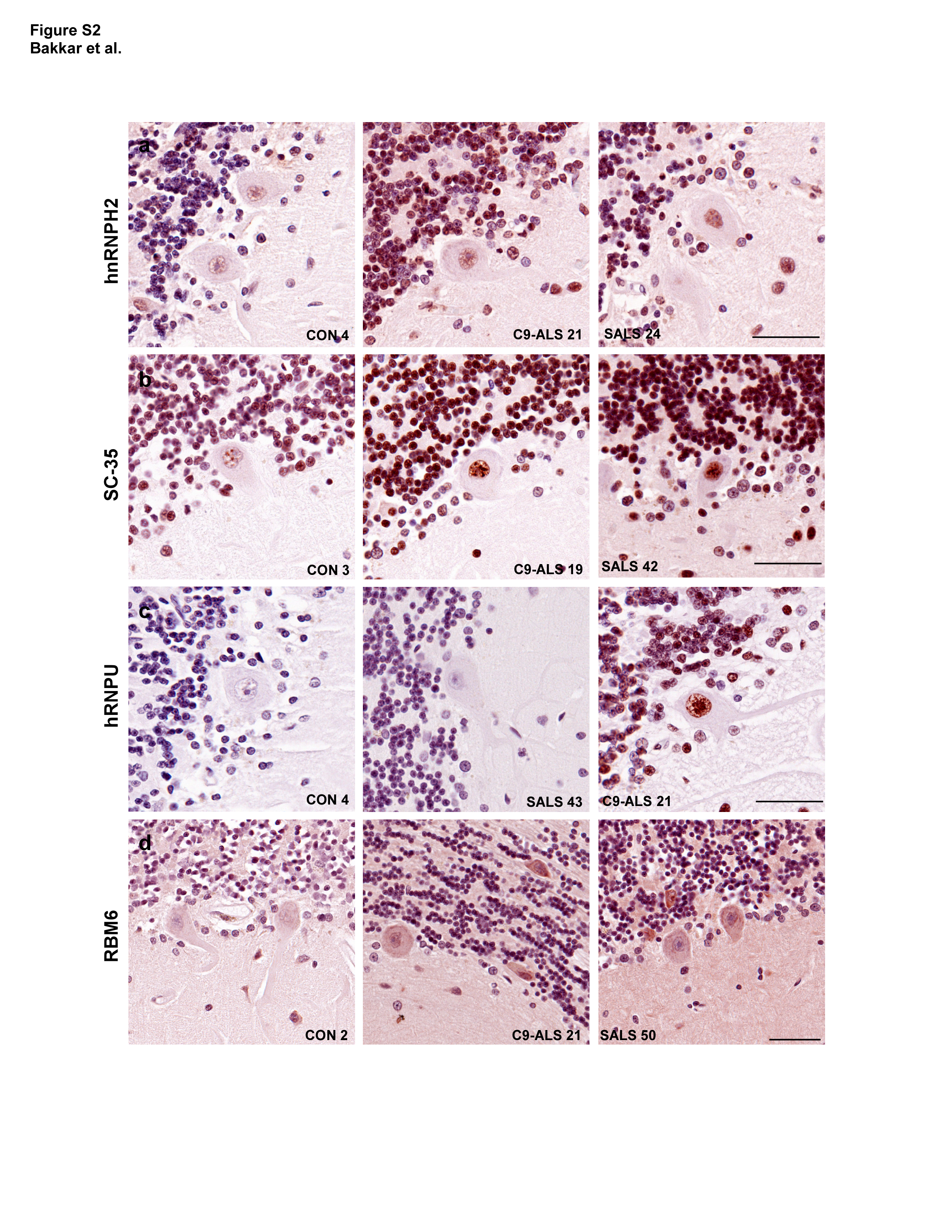

Supplement: Supplementary file 3 — Supplementary material 3 (TIFF 14283 kb) [file 401_2017_1785_MOESM3_ESM.tif]

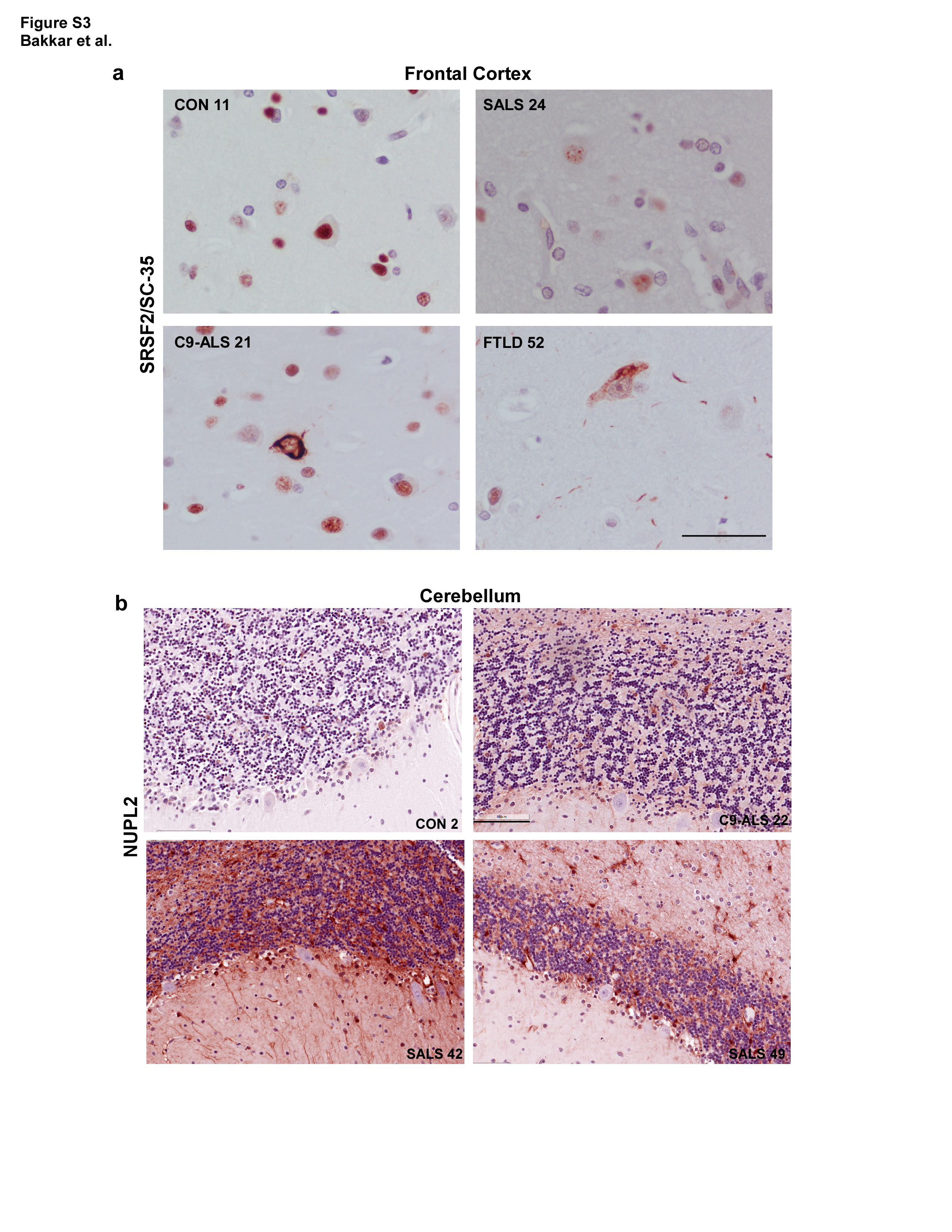

Supplement: Supplementary file 4 — Supplementary material 4 (TIFF 11712 kb) [file 401_2017_1785_MOESM4_ESM.tif]

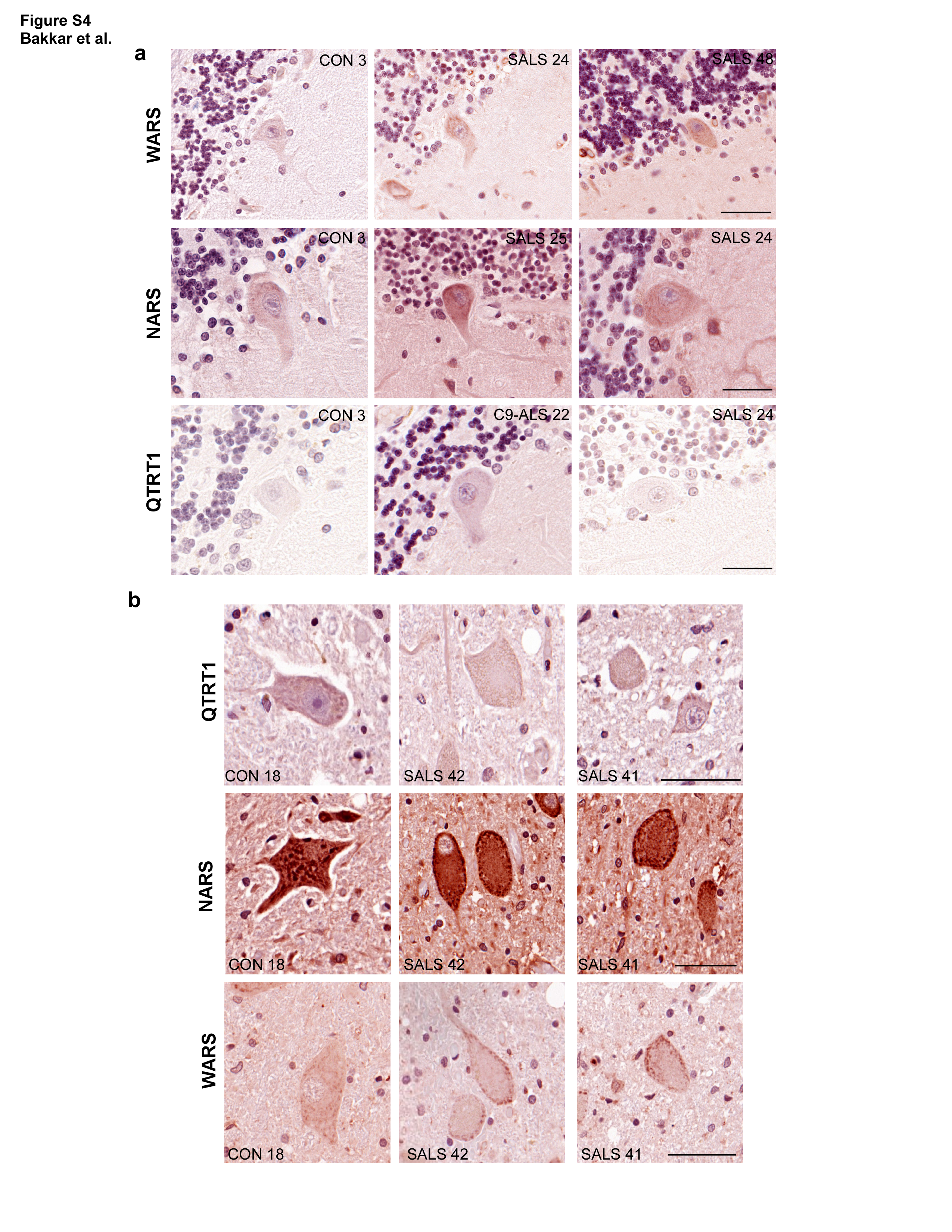

Supplement: Supplementary file 5 — Supplementary material 5 (TIFF 13606 kb) [file 401_2017_1785_MOESM5_ESM.tif]
