# Supplementary material for: Artificial intelligence in neurodegenerative disease research: use of IBM Watson to identify additional RNA-binding proteins altered in amyotrophic lateral sclerosis
Source: Acta Neuropathol. 2017 Nov 13;135(2):227–47. doi: 10.1007/s00401-017-1785-8 (PMC5773659; doi:10.1007/s00401-017-1785-8)
Supplement: Supplementary file 9 — Supplementary material 9 (PDF 616 kb) This file includes a pseudo-code for the algorithm published in reference 48, as well as a detailed explanation of how this code was used to generate the results presented in this paper [file 401_2017_1785_MOESM9_ESM.pdf]

Artificial Intelligence in neurodegenerative disease research: Use of IBM Watson to identify additional RNA binding proteins altered in amyotrophic lateral sclerosis

Authors: Nadine Bakkar[1], Tina Kovalik[1], Ileana Lorenzini[1], Scott Spangler[2], Alix Lacoste[3], Kyle Sponaugle[1], Philip Ferrante[1], Elenee Argentinis[3], Rita Sattler[1], Robert Bowser[1]*

Journal Name: Acta Neuropathologica

Affiliations:

[1]Department of Neurobiology, Barrow Neurological Institute, Phoenix, AZ USA.

[2]IBM Research – Almaden, San Jose, CA USA.

[3]IBM Watson Health, New York, NY USA.

*To whom correspondence should be addressed: Robert Bowser, Department of Neurobiology, Barrow Neurological Institute, 350 W Thomas Road, Phoenix, AZ 85213. Email: Robert.bowser@dignityhealth.org.

Supplementary Pseudocode

```
  public static void run(DocumentList dl, String membership[]) {

           // From the set of documents given as input, create a feature space

           // Parse each document as a set of white space delimited strings.  Remove
stopwords.
           //
           Dictionary dict = dictionary.process(dl,"stopWords.txt");

           // Find the most frequently occurring words and two word phrases
(Bigrams).  Let the most frequent 25,000 of these be the features.
           TextClustering tc = new TextClustering(dl,dict);

           //Create a matrix of feature occurrences over the document collection
           // Label each document based on the query that generated it.
           tc.computeMembership(m);


           // Calculate Centroids – TFIDF feature basis
           int doccounts[] = Taxonomy.getTermDocumentCountTotals(tc);
             for (int i=0; i<tc.nclusters; i++) {
                  int ccounts[] = Taxonomy.getTermClusterDocumentCounts(tc, i);
                  for (int j=0; j<tc.attribNames.length; j++) {
                     if (doccounts[j]==0 || tc.getClusterSizes()[i]==0)
                            tc.centroids[i][j] = 0;
                     else tc.centroids[i][j] =
1.0F*ccounts[j]*tc.ndata/(1.0F*doccounts[j]*tc.getClusterSizes()[i]);
                  }
             }

             // Calculate distances between centroids.
// The Cosine distance between document vectors (X,Y) is defined to be:
```

$$\cos(X, Y) = \frac{X \cdot Y}{||X|| \cdot ||Y||}.$$

```
                  float distances[][] = new float[tc.nclusters][tc.nclusters];
                  float ss[] = new float[tc.nclusters];
                  for (int i=0; i<ss.length; i++)
                  {
                         ss[i] =
(float)Math.sqrt(Util.dotProduct(tc.centroids[i],tc.centroids[i]));
```

```
        }
        for (int i=0; i<result.length; i++)
        {
            for (int j=i+1; j<result.length; j++)
            {
                float denom = ss[i]*ss[j];
                result[i][j] =
distance(tc.centroids[i],tc.centroids[j],denom);
            }
        }
```

///Nearest Neighbor calculate result. This finds the distance from each candidate to the nearest known positive.

```
    result = new double[x.tc.nclusters];
    for (int i=0; i<result.length; i++) {
        for (int j=0; j<x.hotNodes.length; j++) {
           if (x.hotNodes[j]) {
              if (i==j) result[i]++;
              else {
                 if (i==j) result[i]=1.0F;
                 else if (distances[i][j]>result[i]) result[i] = distances[i][j];


              }
           }
        }
    }
```

// Diffusion calculate result.

// The labelvector matrix contains a 1.0 if the entity is a known entity, and 0.0 otherwise.

```
    double degreematrix[] = new double[d.length];
    labelvector = new double[d.length];
    String cn[] = new String[tc.clusterNames.length];
    for (int i=0; i<cn.length; i++) cn[i] = tc.clusterNames[i].replaceAll("\"","");
    for (int i=0; i<labels.length; i++) {
       int pos = Util.findPosition(labels[i],cn);
       if (pos==-1) System.out.println("label not found: " + labels[i]);
       else labelvector[pos] = 1.0;
    }
```

```
// k is set to be the square root of the number of entities in the analysis.  We create a
new distance matrix that has a 1.0 if the entity of the column is the kth closest to the
entity in the row, and a 0.0 otherwise.  This is the “adjacency matrix”.
        int k = (int)Math.sqrt(distances.length);

        for (int i=0; i<distances.length; i++) {
          int order[] = Index.run(distances[i]);
          order = Util.reverse(order);
          for (int j=0; j<order.length; j++) {
                if (j<k) {
                  distances[i][order[j]] = 1.0;
                }
                else distances[i][order[j]] = 0.0;
          }
        }

        for (int i=0; i<distances.length; i++) {
          for (int j=0; j<distances[i].length; j++) {
                if (distances[i][j] == 0.0)
                  distances[i][j] = distances[j][i];
                if (i==j) distances[i][j] = 0.0;
          }
        }

// the degree matrix contains the number of  ones for each entity.
        for (int i=0; i<distances.length; i++) {
          for (int j=0; j<distances[i].length; j++) {
                if (distances[i][j] > 0)
                  degreematrix[i]++;
          }
        }


// the laplacian is the degreematrix minus the distance matrix.
          double laplacian[][] = new double[distances.length][distances.length];
          for (int i=0; i<distances.length; i++) {
                for (int j=0; j<distances.length; j++) {
                  if (i==j) laplacian[i][j] = degreematrix[i] - distances[i][j];
                  else laplacian[i][j] = -distances[i][j];
                }
          }

//alpha is 1/Max(laplacian)
          double alpha = 0.0;
          for (int i=0; i<distances.length; i++) {
                double sumrow = 0.0;
```

```
            for (int j=0; j<distances.length; j++) {
              sumrow = sumrow + Math.abs(laplacian[i][j]);
            }
            if (sumrow>alpha) alpha = sumrow;
        }
        alpha = 1.0/alpha;

// the matrix A is used to solve for Ax=labelvector.  It is alpha times the laplacian
//except on the diagonal, where it is 1+alpha*laplacian.
        double A[][] = new double[distances.length][distances.length];
         for (int i=0; i<distances.length; i++)
             for (int j=0; j<distances.length; j++) {
               if (i==j)
                    A[i][j] = 1.0+(alpha*laplacian[i][j]);
               else A[i][j] = alpha*laplacian[i][j];
             }

        result = SolveDiffusion.solve(A, labelvector );
```

//this subroutine, available in Matlab, will simulate exactly what we do in our code:
https://www.mathworks.com/help/matlab/ref/symmlq.html?s_tid=gn_loc_drop

DIFFUSION SUBROUTINE:

SET error tolerance to 1d-12;
SET number of iterations to 1000;
CALL symmlq(A, Label Vector, tolerance, iterations) to GET prediction vector f
RETURN f
Define *f* : (a set of continuous labels, *f* (i.e. how likely a RBP is to be linked to ALS))

“We solve for *f* by minimizing the sum of the loss and smoothing functions:

$$\left(f - y\right)^T \left(f - y\right) + \mu f^T L f$$

The first term, the loss function, represents the difference between initial *y* and final labels *f*. During diffusion, this function regulates and prevents the loss of the initial labels. The second term, the smoothing function, represents the smoothness of the new labels *f* in the context of the Laplacian matrix *L*. The Laplacian matrix is the matrix representation of the kinase network and defined $L = D - A$. The adjacency matrix, denoted *A*, specifies if kinase *i* is connected to kinase *j* where $A(i,j) = 1$ if the entities are connected and $A(i,j) = 0$ otherwise. The degree matrix, *D*, is a diagonal matrix given by $D_{ii} = \sum_j A(i,j)$. The diffusion coefficient *μ* balances the loss of the initial labels against the smoothness. The previous equation has a closed form solution [2]:

$$f = \left(I + \mu L\right)^{-1} y$$

where I is the Identity matrix. We set the diffusion coefficient μ to the inverse of the Laplacian's norm

$$\mu = \frac{1}{\max_i (\sum_k^n |L_{ik}|)}$$

This value insures that the Hessian is positive definite and the above function is convex. In order to identify new kinases, we then look at the new labels f where the labels with the largest increase will be our targets."

[Spangler, Scott, et al. "Automated hypothesis generation based on mining scientific literature." *Proceedings of the 20th ACM SIGKDD international conference on Knowledge discovery and data mining*. ACM, 2014.]

}

```
// subroutine for comparing two vectors - Cosine Distance
public static float distance(float x[], float y[], float denom)
	{
		if (Util.add(x)==0 || Util.add(y)==0) return(1.0F);

		float val = 0.0F;
		float sum = 0.0F;
		for (int i=0;i<x.length;i++)
		{
			val = x[i]*y[i];
			sum-= Math.abs(val);
		}

		if (denom>0) return(sum/denom);
		return(sum);
	}
```
